# Integrated chemical characterization, metabolite profiling, and pharmacokinetics analysis of Zhijun Tangshen Decoction by UPLC-Q/TOF-MS

**DOI:** 10.3389/fphar.2024.1363678

**Published:** 2024-03-08

**Authors:** Qingheng Tong, Yueyue Chang, Guanxiong Shang, Jiu Yin, Xiaoqi Zhou, Suwei Wang, Xiaofeng Yan, Fangfang Zhang, Suqin Wang, Weifeng Yao

**Affiliations:** ^1^ Jiangsu Collaborative Innovation Center of Chinese Medicinal Resources Industrialization, National and Local Collaborative Engineering Center of Chinese Medicinal Resources Industrialization and Formulae Innovative Medicine, School of Pharmacy, Nanjing University of Chinese Medicine, Nanjing, China; ^2^ Huai’an TCM Hospital Affiliated to Nanjing University of Chinese Medicine, Huai’an, China

**Keywords:** Zhijun Tangshen Decoction, traditional Chinese medicine, compounds absorbed into blood, pharmacokinetics, ultra-high performance liquid chromatography coupled with quadrupole time-of-flight mass spectrometry

## Abstract

Diabetic nephropathy (DN) is the main cause of end-stage renal disease worldwide and a major public issue affecting the health of people. Therefore, it is essential to explore effective drugs for the treatment of DN. In this study, the traditional Chinese medicine (TCM) formula, Zhijun Tangshen Decoction (ZJTSD), a prescription modified from the classical formula Didang Decoction, has been used in the clinical treatment of DN. However, the chemical basis underlying the therapeutic effects of ZJTSD in treating DN remains unknown. In this study, compounds of ZJTSD and serum after oral administration in rats were identified and analyzed using ultra-high-performance liquid chromatography coupled with quadrupole time-of-flight mass spectrometry (UPLC-Q/TOF-MS). Meanwhile, a semi-quantitative approach was used to analyze the dynamic changes in the compounds of ZJTSD *in vivo*. UPLC-Q/TOF-MS analysis identified 190 compounds from ZJTSD, including flavonoids, anthraquinones, terpenoids, phenylpropanoids, alkaloids, and other categories. A total of 156 xenobiotics and metabolites, i.e., 51 prototype compounds and 105 metabolites, were identified from the compounds absorbed into the blood of rats treated with ZJTSD. The results further showed that 23 substances with high relative content, long retention time, and favorable pharmacokinetic characteristics *in vivo* deserved further investigations and validations of bioactivities. In conclusion, this study revealed the chemical basis underlying the complexity of ZJTSD and investigated the metabolite profiling and pharmacokinetics of ZJTSD-related xenobiotics in rats, thus providing a foundation for further investigation into the pharmacodynamic substance basis and metabolic regulations of ZJTSD.

## 1 Introduction

Diabetic nephropathy (DN) is characterized by persistent proteinuria, increased blood pressure, and decreased renal function. Long-term persistent hyperglycemia has an impact on the microvascular system and results in DN, and it is one of the common chronic complications of diabetes mellitus (Bakris et al., 2020). Additionally, DN is a leading cause of end-stage renal disease (ESDR). The damage caused by diabetes can involve almost every structure of the kidney, causing the disease to progress more rapidly than in people with non-DN and leaving patients with a poor long-term prognosis (Selby and Taal, 2020). Nowadays, many clinical treatments focus on controlling blood glucose and blood pressure levels, improving kidney function, and reducing urine protein. However, clinical drugs in the treatment of DN may have significant side effects, such as SGLT-2 inhibitors, which may cause ketoacidosis, hyperphosphatemia, and fracture risk. Hence, it is essential to explore more effective drugs for treating DN (Garofalo et al., 2019; Del Vecchio et al., 2021).

Traditional Chinese medicine is beneficial in reducing the symptoms of diabetes and preventing and treating DN. Here, Zhijun Tangshen Decoction (ZJTSD), which is a prescription modified from the classical TCM Didang Decoction of the *Treatise on Febrile Diseases*, is used to treat DN. ZJTSD is composed of *Rheum palmatum* L. [Polygonaceae; Rhei Radix et Rhizoma], *Hirudo nipponica* Whitman [Hirudinidae; Hirudo], *Astragalus mongholicus* Bunge [Fabaceae; Astragali Radix], *Polygonatum sibiricum* Redouté [Asparagaceae; Polygonati Rhizoma], *Scleromitrion diffusum* (Willd.) R.J.Wang [Rubiaceae; Hedyotis diffusa], and *Salvia miltiorrhiza* Bunge [Lamiaceae; Salviae miltiorrhizae radix et rhizoma] at a ratio of 10:3:30:15:20:15, respectively. The classical Chinese formula includes four elements: the monarch drug, the minister drug, the assistant drug, and the servant drug. Based on the theory of Chinese medicine, the monarch drug plays the most important role in the formula; the minister drug assists in the treatment of diseases; the assistant drug assists the monarch and minister drugs to achieve a better therapeutic effect; and the servant drug can reduce the adverse effects or increase the potency of the entire formula. *R. palmatum* L. and *H. nipponica* Whitman are monarch drugs and have the effect of eliminating blood stasis and unblocking meridians. *S. miltiorrhiza* Bunge is the minister drug and has the function of activating circulation and dispersing stasis. *A. mongholicus* Bunge, *P. sibiricum* Redouté, and *S. diffusum* (Willd.) R.J.Wang are the assistant and servant drugs used for clearing heat and removing dampness, which have the effects of supplementing Qi and nourishing Yin. ZJTSD could reduce proteinuria and improve renal function in patients with early-to-mid-stage DN, and its mechanism may be related to improving the expression of TNF-α, IL-6, and hsCRP and reducing the renal microinflammatory state (Wu, 1999; Wang et al., 2019). Moreover, *H. nipponica* Whitman, *R. palmatum* L., and *S. miltiorrhiza* Bunge are commonly known for their anti-diabetic and anti-diabetic nephropathy properties (Guo et al., 2017; Zeng et al., 2021; Shen et al., 2023). However, the substance basis of ZJTSD in the treatment of DN remains unclear.

The active ingredients of TCM, which can play a more comprehensive role through multiple pathologies, are the essential foundation for the therapeutic efficacy of TCM. Despite reports of clinical benefits from several TCM formulations, the chemical composition and mechanism have not been determined. This requires the development of new methods for the rapid screening of active ingredients to find the exact mechanism, which develops more targeted new methods and new drugs to treat diabetic nephropathy (He et al., 2021; Chen et al., 2022). Unlike the treatment method of a single drug, TCM treats diseases through a multi-compound and multi-target holistic therapeutic approach (Tang et al., 2022). It is widely acknowledged that the desired therapeutic effect is achieved when substances enter the blood and are transported to target sites (Li et al., 2019; Wu et al., 2023). Therefore, it is necessary to characterize the dynamic changes in the content of these compounds within the organism. As a traditional quantitative method, the standard curve method needs to draw a standard curve with standard samples of the compounds to be tested (Du et al., 2019; Wei et al., 2019). However, the metabolites of the prototype compounds of TCM *in vivo* usually exert therapeutic effects as precursor drugs. The determination of the metabolites of the prototype compounds is necessary when assessing the metabolism level of TCM *in vivo*. Due to the varieties of prototype compounds in traditional Chinese medicine and the wide range of metabolites produced through phase I and II metabolism *in vivo*, it is difficult to obtain standard samples for some prototype compounds and metabolites. Furthermore, the hepatic portal blood contains the most comprehensive information about the compounds of drugs entering the blood. Prototype compounds and metabolites originating from the liver and intestines, which persist within the circulatory system, may serve as the fundamental substratum for the comprehensive effectiveness of Chinese medicine (Li et al., 2017). Studies have described the dynamic changes in the composition of hepatic portal blood, suggesting that the dynamic processes of drugs in the hepatic portal vein can reflect, to some extent, their dynamic processes in the systemic circulation (Zhong et al., 2019). Thus, it is vital to systematically analyze compounds in the hepatic portal blood of rats after the oral administration of ZJTSD, particularly the qualitative identification and dynamic changes in the effective compounds *in vivo*, to better understand the bioactive ingredients responsible for the pharmacological effect of ZJTSD and provide a scientific basis for elucidating the substance basis of the efficacy of ZJTSD.

With the application of mass spectrometers with high resolution, high sensitivity, and high mass accuracy, the level of identification of the various compounds of TCM has been greatly improved. By rapidly separating the complex compounds of Chinese medicine using ultra-high-performance liquid chromatography (UPLC) and obtaining the exact molecular weight and structure data using quadrupole time-of-flight mass spectrometry (Q/TOF-MS), the analysis of Chinese medicine or biological samples can be optimized (Wang et al., 2020; Huang et al., 2021). It is worth noticing that a previous study successfully identified and quantified 31 active ingredients in *Forsythia suspensa* (Thunb.) Vahl leaves using UPLC/Q-TOF-MS and high-performance liquid chromatography (Zhou et al., 2021). It has been reported that researchers have developed affinity ultrafiltration combined with UF-UPLC-Q/TOF-MS/MS to identify 11 potential α-glucosidase inhibitors in the leaves of *Cyclocarya paliurus* (Ning et al., 2019). Currently, there are few studies on screening potential bioactive ingredients based on the overall pharmacokinetics of traditional Chinese medicine. It has been reported that a semi-quantitative-based multi-compound pharmacokinetic approach was feasible for *in vivo* metabolism studies of potential active ingredients of TCM (Wang et al., 2011). A previous study reported that the prototype compounds and metabolites were analyzed in the plasma of rats treated with Qingre Xiaoyanning capsule using a semi-quantitative method, and the dynamics of the prototype compounds and metabolites in the plasma were characterized (Lin et al., 2019). Thus, the semi-quantitative method could be applied to the rapid and comprehensive identification of TCM by time-of-flight mass spectrometry (UPLC-QTOF-MS) for further pharmacokinetic analysis of potential active ingredients.

In this study, the chemical compounds of ZJTSD and the metabolic profile of the serum were analyzed via UPLC-Q/TOF MS *in vivo*. Moreover, the prototype compounds and potential metabolites identified in the serum were assessed using a semi-quantitative method to investigate the potential therapeutic compounds of ZJTSD. In conclusion, this study revealed the chemical basis underlying the complexity of ZJTSD and investigated the metabolite profiling and pharmacokinetics of ZJTSD-related xenobiotics in rats, providing insights into the therapeutic potential for DN and the chemical basis for further screening of candidate compounds for pharmacodynamic and mechanism studies.

## 2 Materials and methods

### 2.1 Materials and reagents

The reference standards of ferulic acid (Batch no. 110773-201012), uridine (Batch no. 110742-200517), aloe-emodin (Batch no. 110795-201308), tanshinone IIA (Batch no. PS0056-0025 MG), rutin (Batch no. 100080-201409), astragaloside IV (Batch no. 0781-200311), rhein (Batch no. 0757-200206), and ursolic acid (Batch no. 110742-200517) were purchased from the National Institutes for Food and Drug Control (Beijing, China). Protocatechualdehyde (Batch no. Y-032-180428), quercetin (Batch no. Y-032-180428), cryptotanshinone (Batch no. Y-032-180428), rosmarinic acid (Batch no. M-024-181210), and caffeic acid (Batch no. K-003-181216) were purchased from Chengdu Herbpurify Co., Ltd. (Chengdu, China). Emodin (Batch no. P19A7F13264) and salvianolic acid B (Batch no. P18J9F65871) were purchased from Shanghai Yuanye Bio-Technology Co., Ltd. (Shanghai, China). Oleanolic acid (Batch no. C1419076) was purchased from Shanghai Aladdin Biochemical Technology Co., Ltd. (Shanghai, China). Digoxin (Batch no. C1419076), the internal standard (IS) in the negative ion mode, was purchased from Chengdu Desite Biotechnology Co., Ltd. (Chengdu, China). Clarithromycin (Batch no. FY21B0945), the IS in the positive ion mode, was purchased from Nantong Feiyu Biological Technology Co., Ltd. (Nantong, China). LC-MS grade acetonitrile was purchased from Merck &Co., Inc. (Darmstadt, Germany). LC-MS grade formic acid was purchased from Thermo Fisher Scientific Co., Ltd. (Waltham, MA, United States). The purity of each standard compound was determined to be higher than 98%.

*R. palmatum* L. (Batch no. A220601) was purchased from Bozhou Yonggang Chinese Medicine Co., Ltd. *S. diffusum* (Willd.) R.J.Wang (Batch no. 202101) was purchased from Baicui Jinfang Chinese Medicine Co., Ltd. (Bozhou, China). *P. sibiricum* Redouté (Batch no. 20220301), *H. nipponica* Whitman (Batch no. 20211001), Astragali Radix (Batch no. 20220401), and *S. miltiorrhiza* Bunge (Batch no. 20220101) were provided by Guilin Zhongnan Chinese Traditional Medicine Co., Ltd. (Bozhou, China). All the above traditional Chinese medicinal materials were identified by Professor Qinan Wu from the Nanjing University of Chinese Medicine.

### 2.2 Preparation of ZJTSD

A measure of 3 g *H. nipponica* Whitman, 30 g *A. mongholicus* Bunge, 15 g *P. sibiricum* Redouté, 10 g of processed *R. palmatum* L., 15 g *S. miltiorrhiza* Bunge, and 20 g *S. diffusum* (Willd.) R.J.Wang were immersed in 930 mL (1:10, *w*/*v*) of water for 30 min and boiled for 30 min. The mixture was filtered using a 325-mesh sieve. Then, the boiling process was repeated (1:10, *w*/*v*). The filtrates from both decoction steps were combined and then concentrated to achieve a final density of 4.0 g of crude drug per milliliter for the oral administration in rats. Subsequently, the concentrate was diluted and centrifuged at 18,000 rpm for 10 min, and the resulting supernatant was further filtered using a 0.22-μm filter membrane to a final concentration of 0.18 g of crude drug per milliliter to use for MS analysis.

### 2.3 Animals and drug administration

Thirty male Sprague–Dawley rats (200 ± 20 g) were purchased from the Nanjing Qinglongshan Experimental Animal Center (Nanjing, China). They were maintained at 25°C ± 2°C, 55% ± 5% humidity, and a 12-h light/12-h dark cycle, and had free access to standard diet and water. The method was referred from previous literature (Zhong et al., 2019). All rats were divided randomly into two groups: ZJTSD-dosed group (n = 27) and control group (n = 3). The ZJTSD-dosed group was given the ZJTSD solution by gavage (i.g.) at a dosage of 1 mL/100 g body weight of rats, and the control group was given 0.9% normal saline at the same dose, twice a day for a week. After the last administration, the blood was collected from the hepatic portal vein at 15 min, 30 min, 1 h, 2 h, 4 h, 6 h, 8 h, 12 h, and 24 h, and 3 rats were taken at each time point. Animal welfare and experimental procedures were strictly followed by related ethics regulations of the Nanjing University of Chinese Medicine, and the ethical statement is 202203A015.

### 2.4 Preparation of serum samples

The method described in the published study was used to prepare the samples (Gao et al., 2022). The blood samples of rats were centrifuged at 4,000 rpm for 10 min to obtain the serum. The serum sample (1 mL) was processed with a 3-fold volume using methanol for protein precipitation, with epicoprostanol serving as the internal standard (digoxin and clarithromycin). After vortexing for 3 min, the mixture was centrifuged at 18,000 rpm for 10 min. Subsequently, the supernatant was evaporated at 45°C using a vacuum concentrator. After evaporating the solvent, 150 μL of methanol/water (1:1, *v*/*v*) was added, vortexed, sonicated, and then centrifuged at 18,000 rpm for 10 min at 4°C. The resulting supernatant was used for further analysis.

All the standards were weighed and prepared as stock solutions in methanol. Two IS (digoxin and clarithromycin) stock solutions were mixed and diluted to achieve a mixed IS solution. The first mixed standard working solution was prepared by mixing and diluting ferulic acid, astragaloside IV, protocatechualdehyde, uridine, quercetin, aloe-emodin, rhein, tanshinone IIA, cryptotanshinone, rosmarinic acid, oleanolic acid, caffeic acid, rutin, and the two IS stock solutions. The second mixed standard working solution was prepared by mixing and diluting salvianolic acid B, ursolic acid, emodin, and two IS stock solutions.

### 2.5 Conditions of UPLC-Q/TOF-MS

Liquid chromatographic separation was performed using an AB SCIEX LC™ 2.0 system (AB SCIEX, United States) fitted with an Acquity UPLC BEH C_18_ column (100 mm × 2.1 mm, 1.7 μm; Ireland). The mobile phases in negative and positive ion modes consisted of 0.1% FA aqueous solution (A) and acetonitrile containing 0.1% FA (B). The program of the elution gradient was set as follows: 0∼5 min, 2%∼35 %B; 5∼7 min, 35%∼50%; 7∼10 min, 50%∼70%; 10∼15 min, 70%∼90 %B; 15∼18 min, 90%∼90 %B; 18∼18.5 min, 90%∼2 %B; and 18.5∼20 min, 2%∼2 %B. The injection volume was 3 µL. The flow rate and the column temperature were set at 0.3 mL/min and 40°C, respectively.

The mass spectrometry analysis was performed on a SCIEX ZenoTOF™ 7600 system (AB SCIEX, United States), which was calibrated in the high-sensitivity mode for every 10 samples. Spectra were acquired in the TOF/MS-IDA-TOF MS/MS mode with the ESI source operating in positive and negative ion modes. MS parameters were set as follows: ionization source, ESI; spray voltage, 5,500 kV/–4,500 kV; collision energy, 40 V/–40 V; declustering potential, 80 V/–80 V; ion source temperature, 550°C; and scan range, 100–1250 *m/z*. Data acquisition and processing were performed using SCIEX OS 2.2 and PeakView 2.2.

### 2.6 Data analysis

The data analysis method applied in this study was referenced based on the previous research literature (Gao et al., 2022). Using a combination of literature and the traditional Chinese medicine database and analysis platform (TCMSP), a self-constructed chemical library was created containing essential details such as compound names, molecular formula, compound structure, and accurate molecular mass. To identify the unknown compounds in ZJTSD, the chromatographic characteristics of the available reference compounds were analyzed. The chemical compounds from the self-established library were imported into PeakView 2.2 for the identification of chemical compounds of ZJTSD. Only the compound with an error in the exact mass accuracy within 5 ppm was taken into account and then identified by the analysis of its fragmentation rules in the mass spectra. To differentiate between structural isomers, we introduced the valuable ClogP values analyzed via ChemDraw Ultra 19.0. Generally, compounds with higher ClogP values exhibited longer retention times within reversed-phase liquid chromatography systems (Zhang et al., 2012; Liang et al., 2013).

The prototype compounds detected in the serum were imported into MetabolitePilot 2.0.4. Metabolites and their metabolic pathways were searched and identified. A semi-quantitative method was adopted based on the correction peak area *versus* time (Wang et al., 2011; Lin et al., 2019). The mean serum time-course profile of pharmacokinetics based on the peak area ratio *versus* time was plotted using Origin 2018. The kinetic analysis of the peak area ratio (×1,000)–time curve was calculated using Phoenix WinNonlin 8.1 (Phoenix, American) software and illustrated.

## 3 Results

### 3.1 Identification and characterization of chemical compounds

**FIGURE 1:**
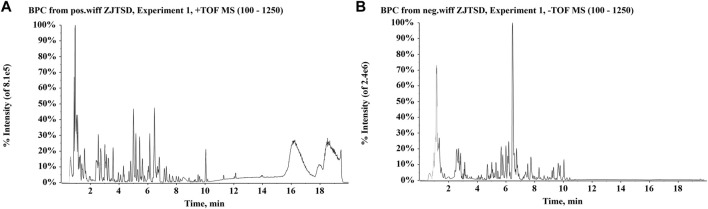
Base peak chromatograms (BPCs) of Zhijun Tangshen Decoction (ZJTSD). **(A)** Positive ion mode and **(B)** negative ion mode.

Chemical compounds were identified by comparing the obtained relative retention time, precise molecular weight, and characteristic fragment ions with parameters in accessible databases, reference standards, and existing literature. A total of 15 compounds of ZJTSD were accurately identified with reference standards, including ferulic acid, astragaloside IV, protocatechualdehyde, uridine, quercetin, aloe-emodin, rhein, tanshinone IIA, cryptotanshinone, rosmarinic acid, oleanolic acid, caffeic acid, rutin, salvianolic acid B, ursolic acid, and emodin. UPLC-Q-TOF-MS identified and tentatively characterized a total of 190 xenobiotics. These compounds included 40 flavonoids, 34 anthraquinones, 11 amino acids, 31 terpenoids, 31 phenylpropanoids, and 43 other categories. The base peak chromatograms (BPCs) of the chemical compounds of ZJTSD are shown in Figure 1, and specific MS data on these compounds are given in Supplementary Table S1.

#### 3.1.1 Flavonoids

**FIGURE 2:**
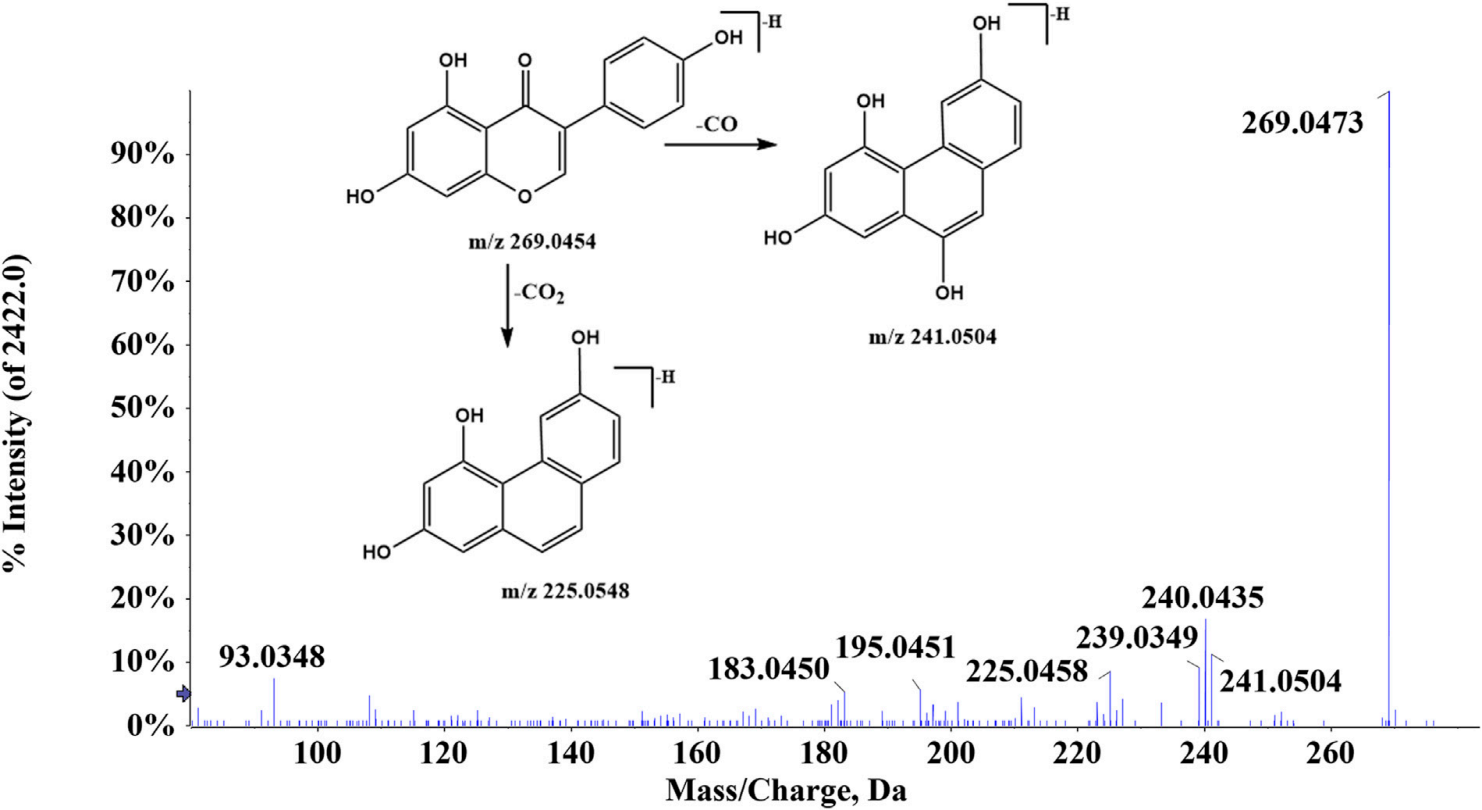
Proposed fragmentation pathway of genistein (compound 6).

**FIGURE 3:**
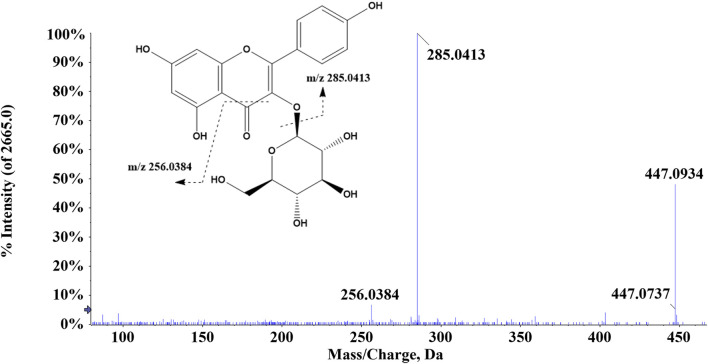
Proposed fragmentation pathway of kaempferol-3-*O*-*β*-D-glucopyranoside (compound 19).

There were 42 flavonoid compounds identified in ZJTSD in this study. The retro-Diels–Alder (RDA) reaction and the loss of radicals (CO, CO_2_, H_2_O, etc.) and neutral small molecules were the dominant fragmentation pathways for aglycones. Compound 6 (t_R_ = 6.10 min) produced the [M-H]^-^ ion at *m/z* 269.0454 and the fragment ions at *m/z* 241.0454 and 225.0548 by eliminating one molecule of CO or CO_2_ at *m/z* 137.0235 (Figure 2), suggesting that compound 6 was preliminarily identified as genistein (Yin et al., 2019). Additionally, compound 19 (t_R_ = 4.94 min) was recognized as kaempferol-3-*O*-β-D-glucopyranoside (Chen et al., 2023), which exhibited characteristic ions at *m/z* 285.0413 [M-H-C_6_H_10_O_5_]^-^ and *m/z* 447.0928 [M-H]^-^. Further analysis revealed that the fragment ion at *m/z* 285.0413 resulted from the loss of CHO (Figure 3).

#### 3.1.2 Anthraquinones

**FIGURE 4:**
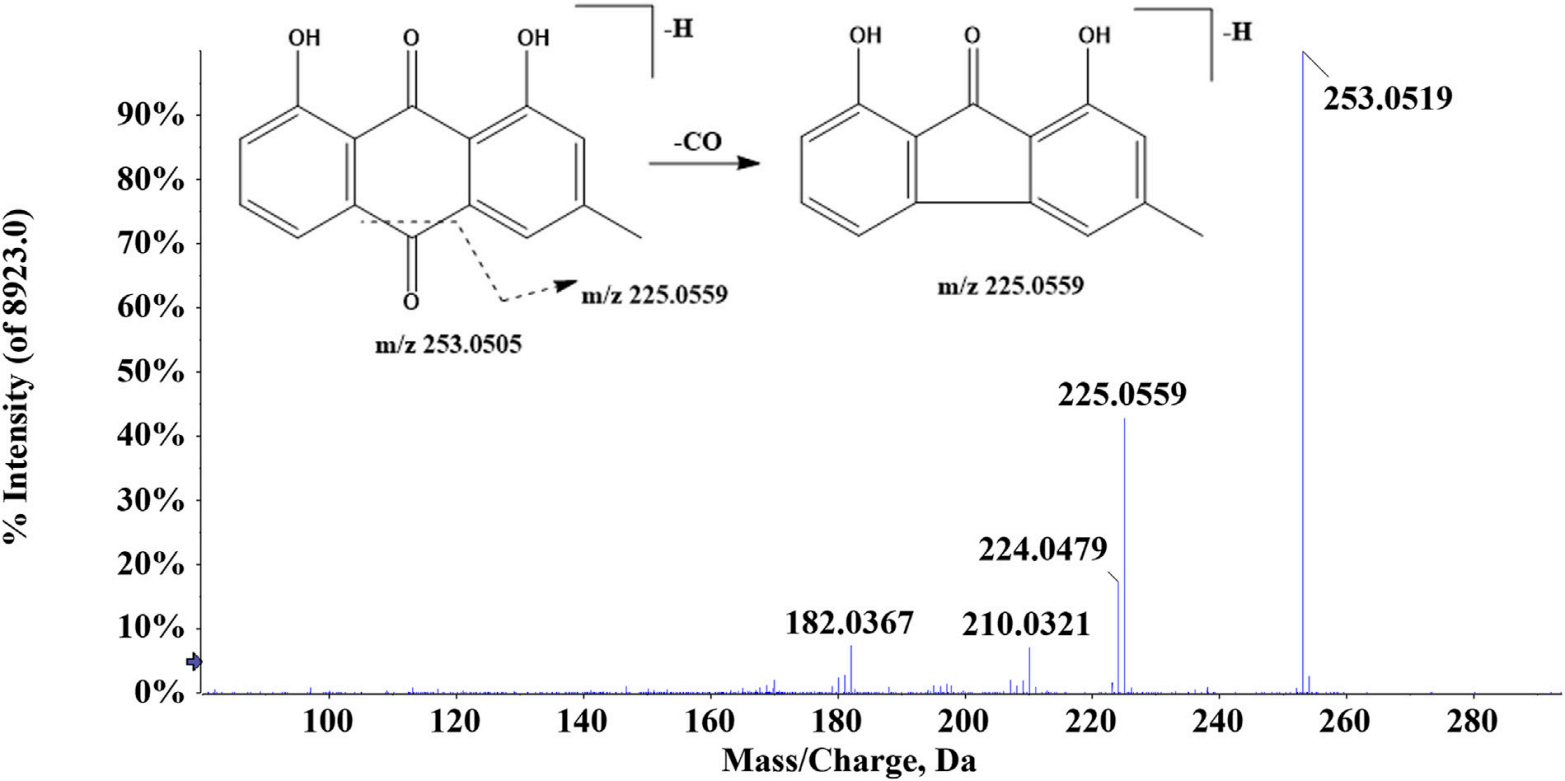
Proposed fragmentation pathway of chrysophanol (compound 43).

In ZJTSD, a total of 34 anthraquinones were identified. Compound 43 (t_R_ = 9.65 min) generated the [M-H]^-^ ion at *m/z* 253.0505 and produced a fragment ion at *m/z* 225.0559 caused by the cleavage of CO, suggesting that compound 43 was tentatively characterized as chrysophanol (Figure 4) (Gong et al., 2022). Compound 49 (t_R_ = 8.47 min) produced the precursor ion [M-H]^-^ at *m/z* 269.0452 and a fragment ion at *m/z* 240.0432 due to the elimination of CHO. The production of the ion *m/z* 239.0352 was caused by the loss of HCHO, and then, the ion *m/z* 239.0352 produced *m/z* 211.0399 and *m/z* 183.0457 after the successive loss of two molecules of CO. Compound 50 exhibited the [M-H]^-^ ion at *m/z* 269.0469. This ion split into *m/z* 225.0566 and *m/z* 241.0514. Compounds 49 and 50 had the same molecular formula and were a pair of structural isomers. The retention time of compounds 49 and 50 was 8.47 min and 10.46 min, respectively, whereas compound 49 had a ClogP value of 2.70, and compound 50 had a ClogP value of 3.61. Based on the theory that compounds in reversed-phase liquid chromatography systems have higher ClogP values, they showed longer retention times. In addition, compound 49 was identified as aloe-emodin based on retention times, fragment ions, and MS/MS spectral patterns of the standards, and compound 50 was characterized as emodin.

#### 3.1.3 Terpenoids

**FIGURE 5:**
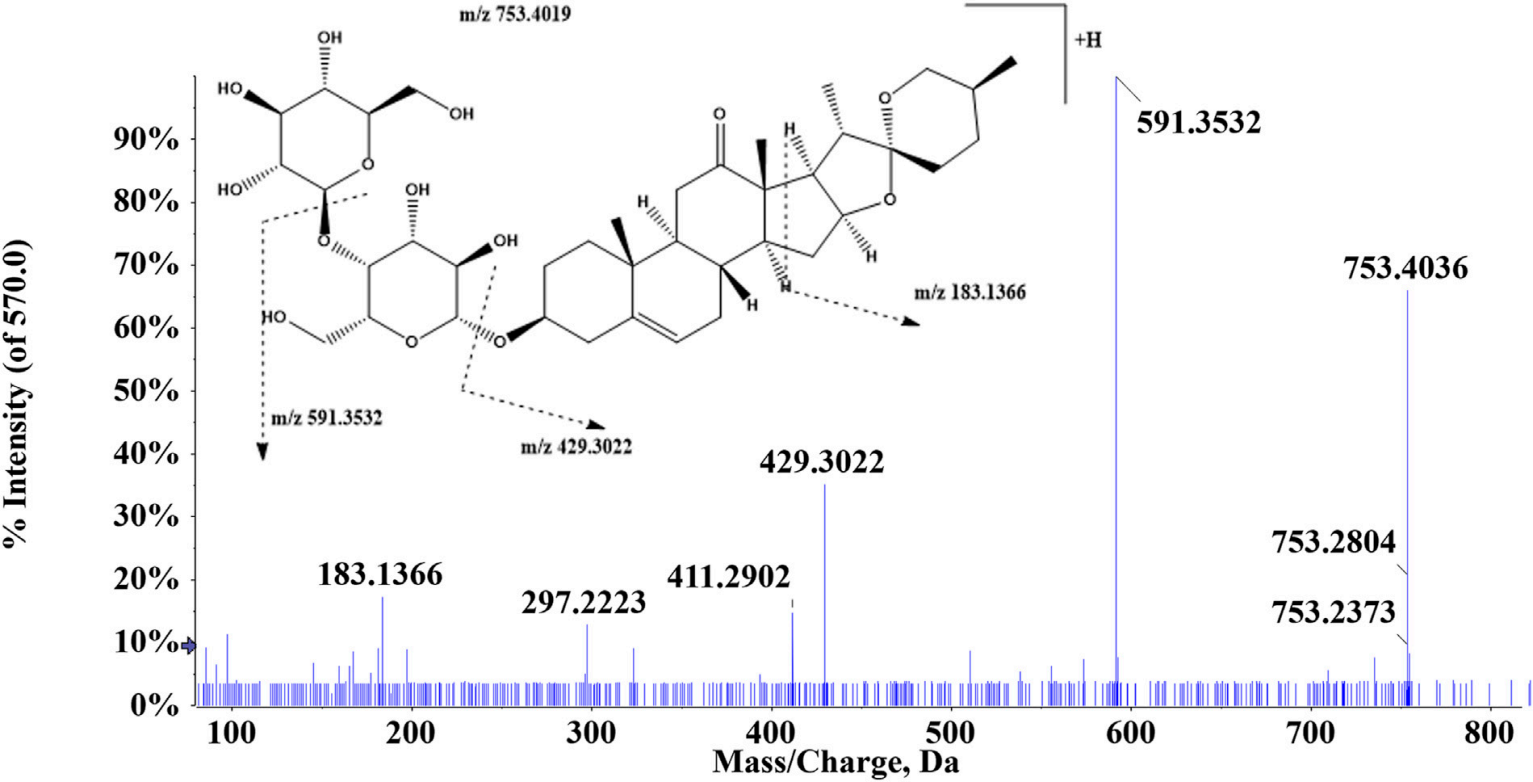
Proposed fragmentation pathway of (25*S*)-kingianoside A (compound 112).

Triterpenoids have a neutral loss identical to that of glucosides, which exhibit comparable ion fragmentation, and the remaining glucosides have H_2_O, CH_3_, and CO loss. A typical example is that compound 112 was eluted at 6.34 min and showed an [M + H]^+^ ion at *m/z* 753.4019, and then in the MS^2^ mass spectra, the precursor ion generated ions at *m/z* 591.3532 and *m/z* 429.4036 by losing 1 and 2 molecules of glucose residue, respectively (Figure 5). Additionally, a fragment [M + H-2Glu-C_16_H_22_O_2_]^+^ existed at *m/z* 183.1366. Above all, compound 112 was suggested to be (25*S*)-kingianoside A. Compound 115 (t_R_ = 7.81 min) showed a quasi-molecular ion at *m/z* 829.4592 [M + HCOOH-H]^-^ in the mass spectra. One molecule of the HCOOH group was eliminated, thus resulting in the generation of a fragment ion at *m/z* 783.4540. By summarizing the above results and comparing fragment ions with the reference standard, it could be speculated that compound 115 may be astragaloside IV. The precursor ion [M + H]^+^ was detected at *m/z* 281.0804 for compound 102 (t_R_ = 8.98 min). The fragment ions at *m/z* 253.0856 and *m/z* 263.0698 were produced through the loss of 1 molecule of CO and H_2_O, respectively. In addition, the precursor ion lost 1 molecule of H_2_O and CO to produce the ion *m/z* 235.0749, and *m/z* 207.0802 was 28 Da less than *m/z* 235.0749, suggesting that the carbonyl group was lost. Thus, compound 102 was presumed to be nortanshinone.

#### 3.1.4 Amino acids

**FIGURE 6:**
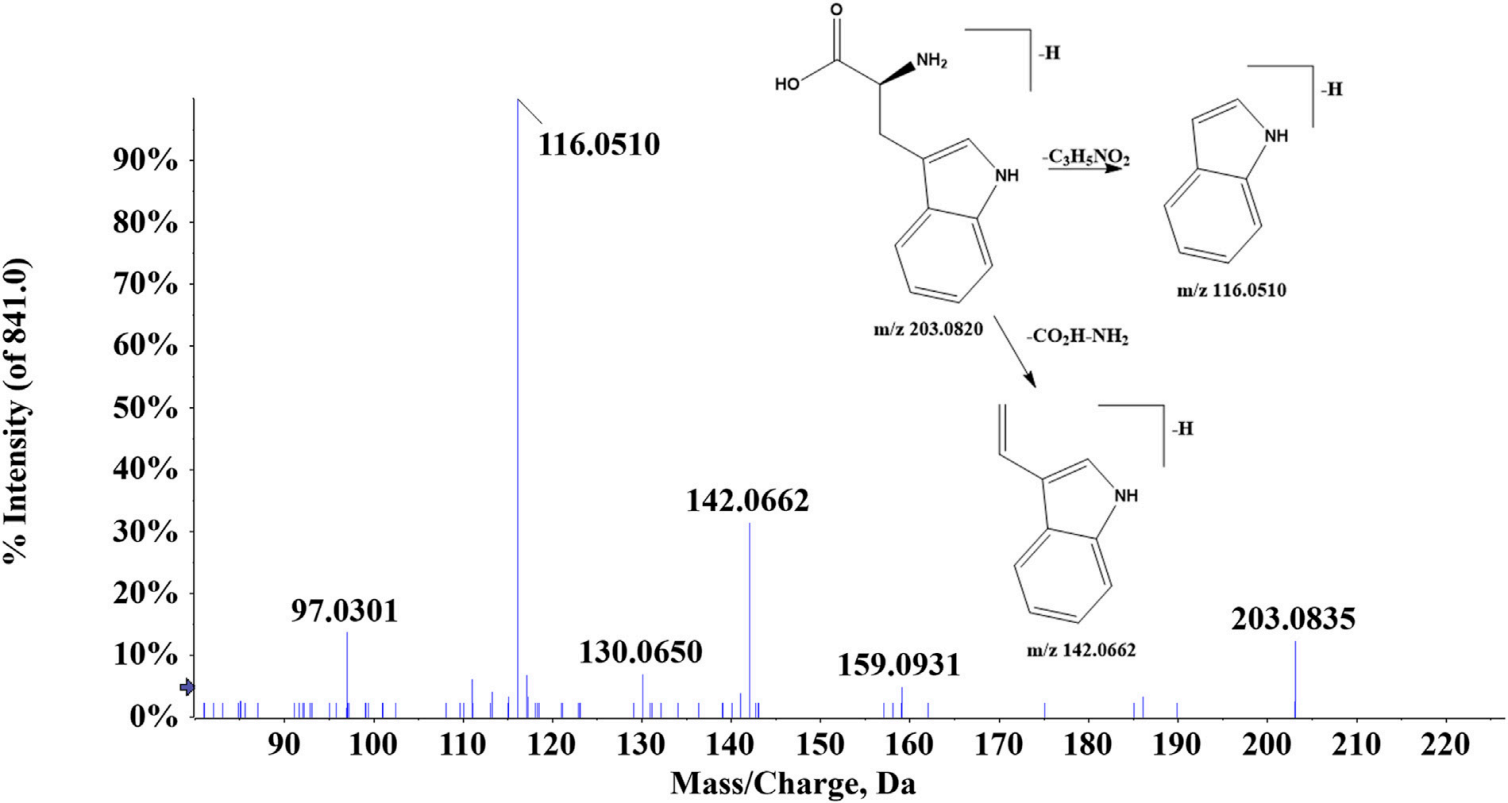
Proposed fragmentation pathway of tryptophan (compound 80).

A total of 11 amino acids were found in ZJTSD. Compound 80 (t_R_ = 3.19 min) indicated the [M-H]^-^ ion at *m/z* 203.0820 and the fragment ions [M-H-C_3_H_5_NO_2_]^-^ at *m/z* 116.0510 and [M-H-CO_2_H-NH_2_]^-^ at *m/z* 142.0662, which were suggested to be tryptophan (Figure 6). Compound 84 (t_R_ = 1.38 min) had the quasi-molecular ion [M + H]^+^ at *m/z* 150.0574, which exhibited characteristic ions at *m/z* 104.0525 [M + H-COOH]^+^, *m/z* 133.0311 [M + H-NH3]^+^, and *m/z* 102.0547 [M + H-CH_3_SH]^+^. In addition, the ion *m/z* 87.0258 was produced by the loss of CH_2_S from *m/z* 133.0311 and NH from *m/z* 102.0547. Compound 84 was identified to be methionine by comparing with the study by Li et al. (2021).

#### 3.1.5 Alkaloids

This study identified a total of seven alkaloids by comparing retention time, accurate mass, and abundance fragment information. Compound 161 showed the quasi-molecular ion [M + H]^+^ at *m/z* 166.0857 with the molecular formula C_9_H_11_NO_2_. The characteristic ions at *m/z* 136.0753 and *m/z* 148.0801 were produced by the orderly elimination of HCHO and H_2_O units, respectively. The ion at *m/z* 118.0644 was 18 Da less than *m/z* 136.0753 and 28 Da less than *m/z* 148.0801, suggesting the removal of HCHO and H_2_O. Therefore, it was presumed that compound 161 was regarded as polygonatine A. Similarly, compound 120 was tentatively postulated to be betaine by referring the study by Zhao et al. (2020).

#### 3.1.6 Phenylpropanoids

A total of 31 phenylpropanoids were identified in this study. An [M + H]^+^ ion at *m/z* 193.0494 was produced by compound 153 (t_R_ = 19.56 min). Its distinctive fragment ion at *m/z* 178.0260 was generated by losing CH_3_, whereas the fragment ion at *m/z* 133.0283 was generated by losing CH3 and CHO. As a result, compound 153 was identified as scopoletin (Tan et al., 2017). The precursor [M-H]^-^ ion at *m/z* 365.1040 was observed for compound 126 (t_R_ = 7.18 min). It was identified as cinnamic acid by the characteristic dominant fragment ion at m/z 103.056 (He et al., 2022). The precursor ion [M + H]^+^ of compound 152 was *m/z* 179.0334 at 4.33 min. It produced *m/z* 133.0280 and *m/z* 151.0383 fragments by removing CO_2_ and CO, respectively. In addition, the elimination of CO produced *m/z* 123.0433, and then, the loss of H_2_O generated *m/z* 105.0336. Therefore, compound 152 was characterized as esculetin (Liu et al., 2023).

#### 3.1.7 Others

A total of 34 compounds were identified from other species, including steroids, organic acids, nucleosides, purines, phospholipids, and phenolics. The [M + H]^+^ ion at *m/z* 268.1033 of compound 181 produced the fragments ions of [M + H-C_5_H_8_O_4_]^+^ at *m/z* 136.0616. Then, NH_3_ was lost from the remaining residue and was determined as adenosine (Sang et al., 2021). Compound 177 was detected in the positive ion mode at *m/z* 153.0401, with the primary fragments found at *m/z* 110.0345 and *m/z* 136.0135, corresponding to the loss of CN and NH_3_ from the precursor, respectively. This suggested that substance 177 was 2,6-dihydroxypurine. Compound 187 (C_24_H_52_NO_6_P.) had the protonated ion [M + H]^+^ at *m/z* 482.3593. The fragment ions *m/z* 104.1065 and *m/z* 184.0726 are produced by the cleavage of P-O and C-O bonds, respectively. As a result, it is assumed that chemical 187 was 1-*O*-hexadecyl-sn-glycero-3-phosphocholine. Compound 165 showed the [M-H]^-^ ion at *m/z* 243.0612, and the corresponding molecular formula was C_9_H_12_N_2_O_6_. The fragment ions were *m/z* 110.0249 and 82.0303, produced by the sequential loss of ribose (C_5_H_8_O_4_, 132 Da) and carbonyl (CO, 28 Da), respectively. The above data indicated that compound 165 could be identified as uridine.

### 3.2 Identification of compounds of ZJTSD absorbed into blood

**FIGURE 7:**
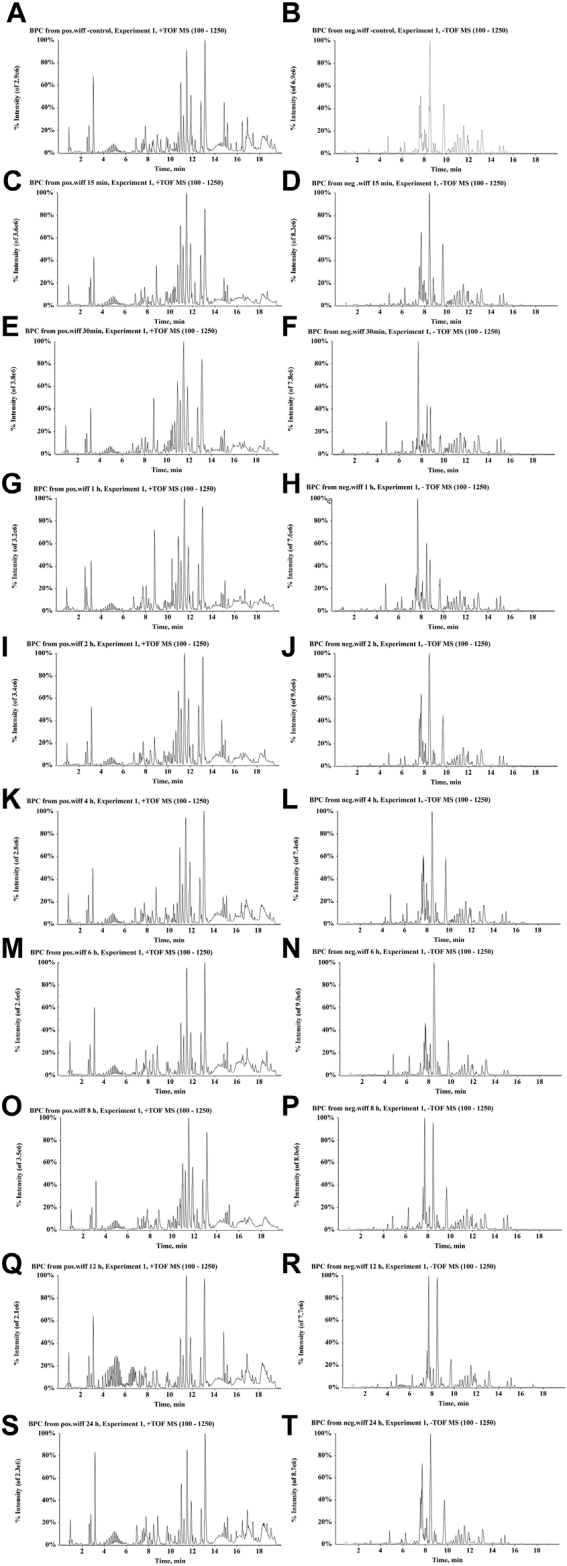
BPCs after the oral administration of ZJTSD in rats at different time points. For positive ion mode: **(A)** control group; **(C)** 15 min; **(E)** 30 min; **(G)** 1 h; **(I)** 2 h; **(K)** 4 h; **(M)** 6 h; **(O)** 8 h; **(Q)** 12 h; and **(S)** 24 h after administration. For negative ion mode: **(B)** control group; **(D)** 15 min; **(F)** 30 min; **(H)** 1 h; **(J)** 2 h; **(L)** 4 h; **(N)** 6 h; **(P)** 8 h; **(R)** 12 h; and **(T)** 24 h after administration.

UPLC-Q/TOF-MS coupled with PeakView and MetabolitePilot processing software was used to investigate the serum metabolic profile of rats following the oral administration of ZJTSD. A total of 156 compounds related to ZJTSD were detected, of which 51 were prototype compounds and 105 were metabolites in the serum. The majority of these compounds were related to flavonoids, anthraquinones, and terpenoids. It mainly underwent metabolic reactions of phase I such as reduction, oxidation, and demethylation and metabolic reactions of phase II such as glycosylation, acetylation, methylation, glucuronidation, hydrolysis, sulfation, and hydrogenation. After the oral administration of ZJTSD, BPCs of prototype compounds and metabolites in negative and positive ion modes are shown in Figure 7. The data on the prototype compounds and metabolites of ZJTSD in the rat serum are shown in Supplementary Tables S2, S3.

#### 3.2.1 Characterization of flavonoid-related prototype compounds and metabolites

In this study, a total of 10 prototype compounds of flavonoids were detected *in vivo*. P8 was identified as methylnissolin with [M + H]^+^ at *m/z* 301.1054. The major fragment ions including *m/z* 167.0695, *m/z* 134.0346, and *m/z* 152.0458 were matched with the mass spectral information about methylnissolin in the literature (Zhang et al., 2023). P9 induced a molecular ion [M + H]^+^ at *m/z* 447.1275 and produced a fragment ion at *m/z* 285.0751 by the cleavage of a glycosidic bond. The fragment ion at *m/z* 270.0522 was generated by losing a CH_3_ group. Thus, P9 could be identified as glycitin.

**FIGURE 8:**
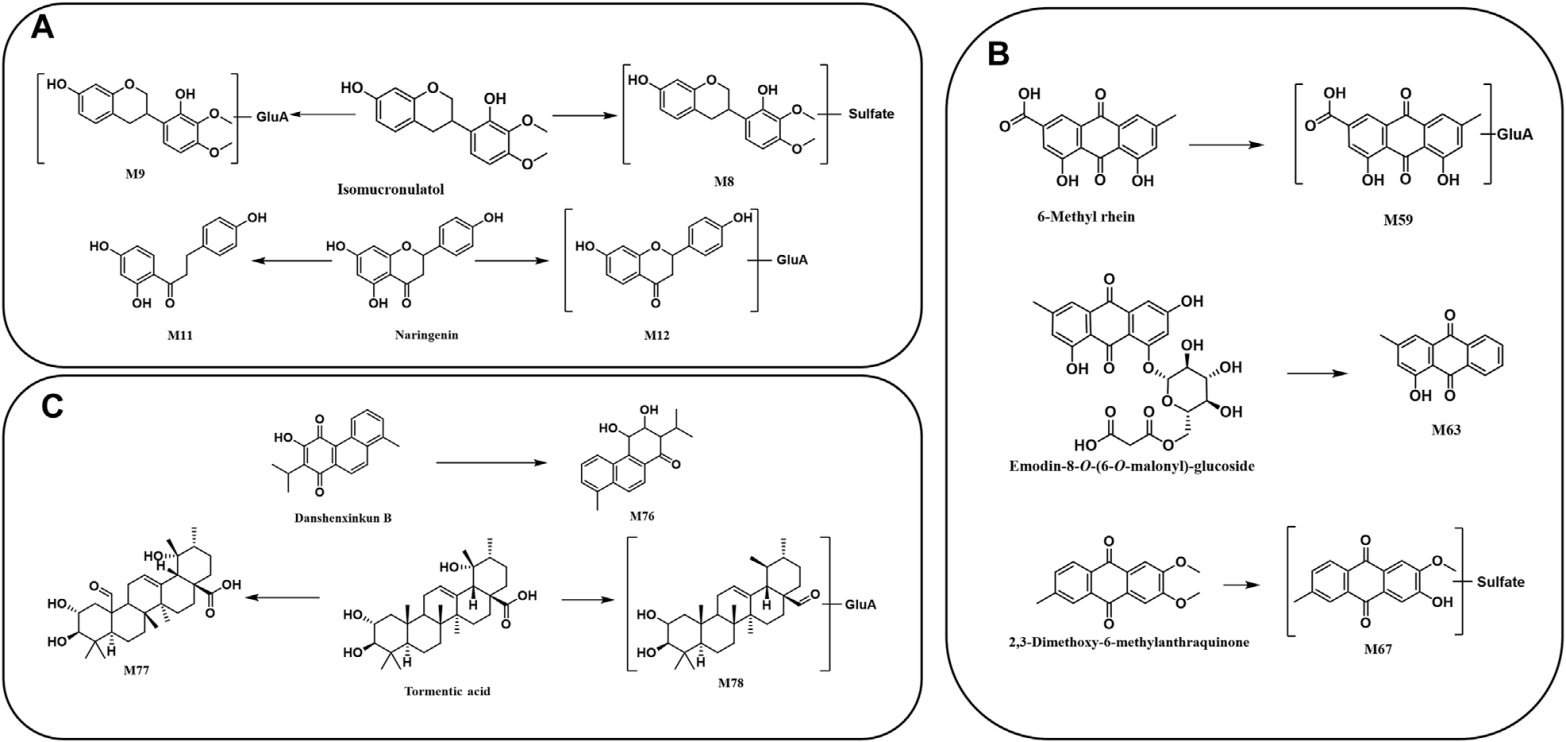
Proposed metabolic pathway of prototype compounds: **(A)** flavonoids, **(B)** anthraquinones, and **(C)** terpenoids.

There were 18 flavones as metabolic prototype compounds, which generated metabolites. The molar weights of M8 and M9 were 80 Da (SO_3_) and 176 Da (GluA) higher than those of isomucronulatol, respectively, which might be the sulfated and glucuronidated products of isomucronulatol, respectively (Figure 8A). M12 is suggested to contain a glucuronide structure by the ion [M-H-176]^-^ at *m/z* 255.0669. Meanwhile, ions with *m/z* 149.0253 and *m/z* 135.0091 suggested that it contained a flavonoid parent nucleus. Additionally, M12 was 16 Da smaller than naringenin, indicating that it was a deoxy and glucuronidated product of naringenin. Similarly, the molecular weight of M11 was 14 Da higher than that of naringenin, suggesting that M11 is the product of the C-ring dehydroxylation of naringenin (Figure 8A). The metabolic pathways of other flavonoids are shown in Supplementary Figure S1.

#### 3.2.2 Characterization of anthraquinone-related prototype compounds and metabolites

A total of 12 prototype compounds of anthraquinones were detected *in vivo*. The [M-H]^-^ ion at *m/z* 297.0406 of P19 produced the fragments ions at *m/z* 253.0610 and *m/z* 225.0584, respectively, which indicated a loss of molecules of CO and CO_2_. Therefore, P19 might be 6-methyl-rhein (Zhang et al., 2020).

The main metabolites from *R. palmatum* L. were anthraquinone-related *in vivo*. M67 (C_16_H_12_O_7_S) provided a precursor ion [M-H]^-^ at *m/z* 347.0214 with the sequential loss of CH_3_ (15 Da) and conjugation of SO_3_ (80 Da), corresponding to a mass weight of 65 Da over 2,3-dimethoxy-6-methylanthraquinone. Therefore, it was supposed to be the de-methylation and sulfate conjugation metabolite of 2,3-dimethoxy-6-methylanthraquinone (Figure 8B). M63 (C_15_H_10_O_3_) had the quasi-molecular ion [M-H]^-^ at *m/z* 237.0536, and its fragment ion *m/z* 209.0596 was produced by losing CO. Additionally, the mass of M63 was 264 Da less than that of chrysophanol-8-*O*-(6-*O*-malonyl)-glucoside [M-H]^-^, which was consistent with a malonyl glucosyl mass. Based on the above data, it could be speculated that M63 was a metabolite of chrysophanol-8-*O*-(6-*O*-malonyl)-glucoside, resulting from the breakage of the glycosidic bond. M59 presented a deprotonated molecular [M-H]^-^ ion at *m/z* 473.0710, which was 176 Da higher than 6-methyl-rhein, suggesting that it may be a glucuronidated product of 6-methyl-rhein (Figure 8B). The metabolic pathways of anthraquinones are shown in Supplementary Figure S2.

#### 3.2.3 Characterization of terpenoid-related prototype compounds and metabolites

In the negative ion mode, tanshinone V, miltionone I, and miltionone II were found in the positive ion mode, while scandoside, deacetyl asperulosidic acid, anshenxinkun A, and tanshinone VI or przewaquinone C were found. Their mass spectrometry information is shown in Supplementary Table S2.

M76 was found at 10.29 min in the negative ion mode, with a fragment ion at *m/z* of 283.1325. The product ion at *m/z* 255.1394 was produced by losing CO, whereas *m/z* 239.1071 was produced by losing CHO and CH, which revealed that the molecular structure of M76 contains methyl, carbonyl, or enolic hydroxyl groups. In addition, M76 was 4 Da larger than danshenxinkun B (Figure 8C). Therefore, M76 was tentatively identified as a hydrogenation metabolite of danshenxinkun B with the carbonyl and carbon–carbon double bonds being hydrogenated. M77 (C_30_H_46_O_6_) and M78 (C_36_H_56_O_9_) were identified as the metabolite of tormentic acid. M77 was the result of the oxidation of tormentic acid, which oxidizes a methyl to a carbonyl group. M78 provided the [M-H]^-^ ion at *m/z* 631.3823, and M78 was a metabolite of the loss of oxhydryl group and conjugated with glucuronide (Figure 8C). The metabolic pathways of other terpenoids are shown in Supplementary Figure S3.

### 3.3 Multi-compound pharmacokinetics of ZJTSD

The pharmacokinetic study was conducted to simultaneously monitor the 156 components of ZJTSD, i.e., 51 prototype compounds and 105 metabolites, exposed in the serum after oral administration by a semi-quantification method. The mean serum time-course curve of pharmacokinetics based on the peak area ratio *versus* time is presented in Supplementary Figures S6, S7. The analysis of the peak area ratio *versus* the time curves was calculated using Phoenix WinNonlin 8.1 software (Supplementary Tables S4, S5).

**FIGURE 9:**
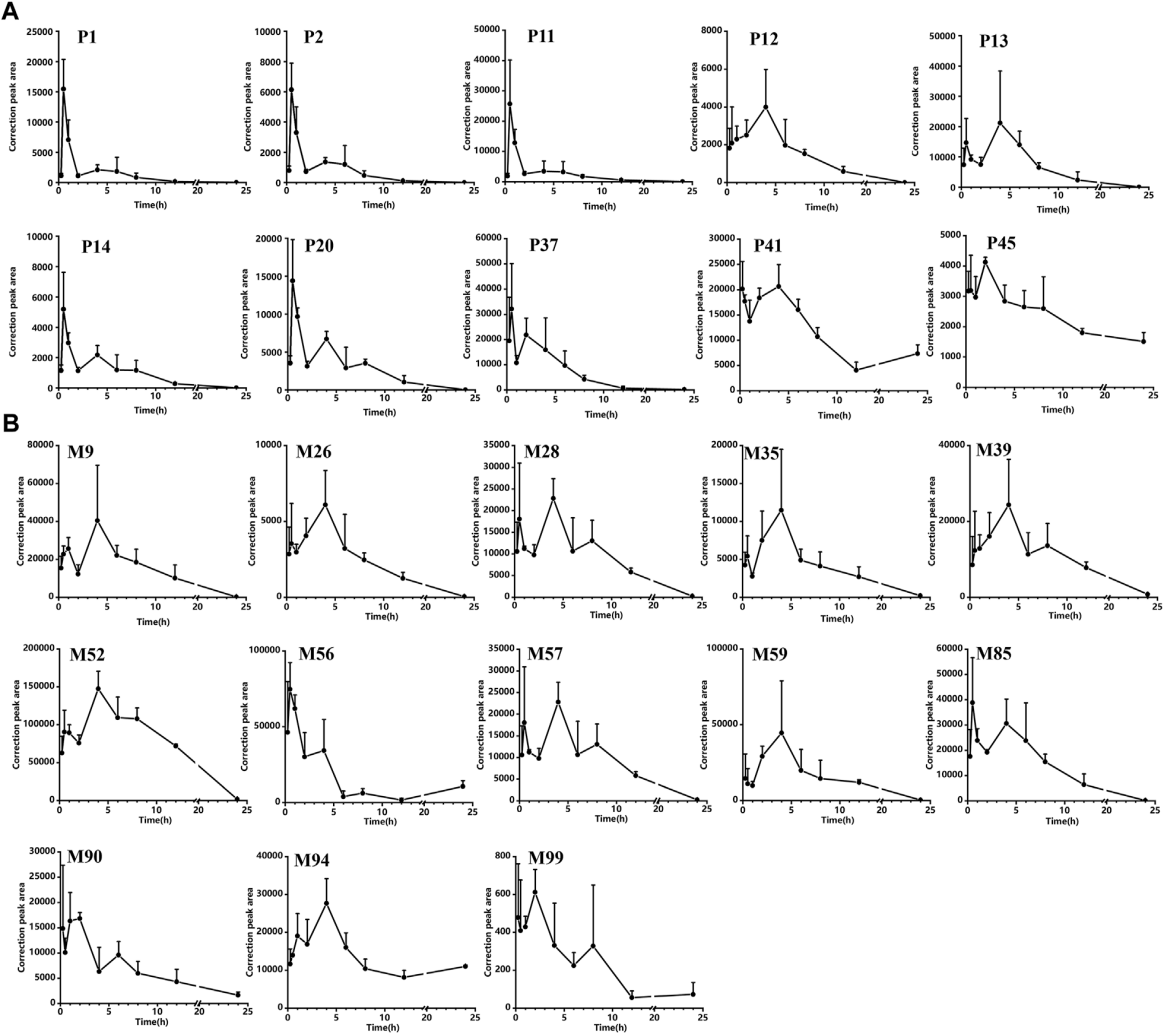
Correction peak area–time curves for compounds of ZJTSD absorbed into the rat serum: **(A)** prototype compounds and **(B)** possible metabolites.

Supplementary Figure S6 shows the concentration *versus* time curves of these compounds in rats corresponding to the ZJTSD group. Pharmacokinetic parameters are shown in Supplementary Table S5. All flavonoids in the serum were detected at 15 min after administration. Except for P47, prototypical compounds reached T_max_ in rats within 0.5–4 h, indicating that the prototype compounds in ZJTSD had fast absorption. Double peaks were observed in some types of components, including prototype compounds of flavonoids, anthraquinones, terpenoids, alkaloids, and phenylpropanoids, occurring mainly within 8 h of dosing. This phenomenon may be related to the dual absorption, enterohepatic circulation, intestinal efflux, and metabolism of their corresponding glucosides (Roberts et al., 2002; Zhang et al., 2007; Zhang et al., 2021). Nevertheless, terpenoids (except P28) were absorbed and eliminated more slowly, which meant that substances remained in the blood for long periods and were exposed to high concentrations. Flavonoids (P1 and P2), anthraquinones (P11, P12, P13, P14, and P20), and phenylpropanoids (P37) indicated a higher level of exposure in the blood, which merits pharmacodynamic assessment. These compounds exhibited a high AUC and the maximum concentration (C_max_). With the exception of P40 and P12, the T_max_ values of these compounds were less than 2 h, the elimination half-life (t_1/2_) was greater than 2.17 h, and the MRT values were over 3.13 h, indicating more rapid absorption, slower elimination, and a more long-lasting effect. It was worth noting that P40 and P45 had a half-life of 21 h. The MRT values were 9.56 h and 9.91 h, respectively. The AUC value of P40 was the largest, suggesting that P40 and P45 remained *in vivo* for a longer period of bioavailability. In brief, comparing the dynamics of each composition, the initial choice of the 10 prototype compounds as the active candidate ingredients is as follows: P1, P2, P11, P12, P13, P14, P20, P37, P40, and P45 (Figure 9A).

As shown in Supplementary Figure S7, the metabolism of the prototype compounds through phase I and phase II produced potential metabolites that were rapidly distributed and slowly metabolized. According to the non-compartment model parameters T_max_, t_1/2_, and AUC (shown in Supplementary Table S5), M47, M56, and M90 required a faster time to peak; the C_max_ of M9, M26, M28, M35, M56, M85, M90, M94, and M99 was reached within 3.5 h. The C_max_ of M39 and M52 was reached after 4 h. The t_1/2_ of most metabolites, such as M9, M26, M28, M35, M57, and M85, was less than 4 h. However, the t_1/2_ of M56, M90, M94, and M99 was longer than 7.5 h. M52 and M56 demonstrated higher peak concentrations. The majority of metabolites of flavonoids and anthraquinones presented a double-peak phenomenon and possessed similar dynamic curves. The AUC values of the serum concentration were greater in M26, M28, M39, M52, M57, M56, M57, M59, M61, M74, M85, M90, and M94 than in others, reflecting that these may have better bioavailability *in vivo*. In summary, the potential metabolites M9, M26, M28, M35, M39, M52, M57, M56, M59, M85, M90, M94, and M99 had better pharmacokinetic behavior than other metabolites; thus, they may exert a more active effect (Figure 9B).

## 4 Discussion

For a complex Chinese decoction, it is believed that only a few key compounds with favorable drug-like properties are responsible for the therapeutic effect of the medicine, rather than all the compounds present (Li et al., 2022). These key compounds of traditional Chinese medicines are the basis for the multi-compound and multi-target properties of treating diseases. However, few studies have reported on the screening of potential active ingredients based on the pharmacokinetics of the whole TCM formula. To analyze the potential bioactive ingredients in ZJTSD, it is required to further analyze the *in vivo* pharmacokinetic profiles of the numerous absorbed compounds in order to determine the optical time-course behavior for a more comprehensive analysis. This study developed a highly sensitive, high-resolution UPLC-Q/TOF-MS method to determine the compounds of ZJTSD. Additionally, after the oral administration of ZJTSD, the key compounds were identified based on the absorption of the compounds in rat serum. This is the first comprehensive characterization of the chemical composition of ZJTSD. A total of 190 compounds, including flavonoids, anthraquinones, phenylpropanoids, and terpenoids, were identified in ZJTSD in both positive and negative ion modes. Among them, 42 were from *R. palmatum* L., 12 were from *H. nipponica* Whitman, 29 were from *A. mongholicus* Bunge, 12 were from *P. sibiricum* Redouté, 34 were from *S. diffusum* (Willd.) R.J.Wang, 45 were from *S. miltiorrhiza* Bunge, and 16 were from several different drugs. Meanwhile, the rapid identification method of several types of compounds was preliminarily established, which enriched the mass spectrometry information about various compounds of ZJTSD, and the cleavage rules provided an important basis for the structural identification of various compounds of ZJTSD, laying the foundation for future research on ZJTSD.

The composition of ZJTSD was complex, and after oral administration, the compounds were transformed in the organism, thus making the composition of exogenous small-molecule compounds even more complicated. Multi-compound pharmacokinetics can be performed using semi-quantitative analysis based on peak area *versus* time. It also helped screen compounds which may be candidates for further pharmacodynamic and mechanism evaluation. The approach could be a way to provide identification methods for unclear metabolites and non-standard substances. Accurate analysis of the pharmacokinetic behavior of bioactive ingredients in ZJTSD could provide quick and reliable time-course information about the compound for further investigations. After analyzing the ZJTSD compounds that were potentially absorbed into the blood, a total of 51 prototype compounds and 105 metabolites were identified at different time points. Flavonoids, the main compounds of ZJTSD, have significant therapeutic effects in diabetic nephropathy (Hu et al., 2021; Li et al., 2022). Flavonoids in ZJTSD mainly underwent metabolic reactions of oxidation, glucuronidation, sulfate conjugation, hydrolysis, reduction, and methylation. The flavonoid glycosides in ZJTSD underwent hydrolysis of the glycosidic bonds during metabolism *in vivo*, followed by glucuronidation and other metabolic reactions to enhance the solubility of the compounds (Zhao et al., 2019; Naeem et al., 2022). For example, the metabolites M17 and M18 of liquiritin were detected *in vivo*, whereas liquiritin, a compound of the botanical drug, was not detected in the serum. M9, M10, and M12 are flavonoid glucuronidation metabolites, which have a relatively high blood concentration *in vivo* and may serve as key compounds. It has also been reported that flavonoids could undergo phase II metabolism in the human small intestine and liver, in which glucuronidation is the main metabolism (Chen et al., 2022). The prototype compounds of anthraquinone compounds and their metabolites were mainly derived from the *R. palmatum* L. In particular, emodin, aloe-emodin, and rhein were absorbed as prototype compounds. The anthraquinone compounds such as emodin, aloe-emodin, and rhein went through glucuronidation and sulfation, while rhein could also be present in the blood in prototype form (Dong et al., 2020). Based on the identified metabolites, the major biotransformation of anthraquinone included reduction oxidation of phase Ⅰ and glucuronidation and sulfation of phase Ⅱ *in vivo*. The metabolites of the terpenoids were mainly derived from *S. diffusum* (Willd.) R.J.Wang and *S. miltiorrhiza* Bunge, with reduction and glucuronidation as the main metabolic pathways. Metabolites of the other categories were mainly produced through reduction and methylation of organic acids and phospholipid compounds. The phenylpropanoids and alkaloids were basically in the blood as prototype compounds. All of these prototype compounds and metabolites identified in this work could be used to elucidate the substance base of ZJTSD.

An increasing number of studies have shown that TCM is a multi-target drug (Huo et al., 2022). The compounds explored in this study have also been reported in the treatment of diabetic nephropathy. Studies have shown that the anthraquinone glycoside extract of *R. palmatum* L. significantly improved T2DM induced by high-fat diet feeding combined with streptozotocin administration through the modulation of the gut microbiota (Cui et al., 2019). Emodin treatment improved urinary albumin, serum creatinine, and blood urea nitrogen levels in DN mice and attenuated foot cell apoptosis by inhibiting the PERK-eIF2α signaling pathway *in vitro* and *in vivo*, suggesting a possible therapeutic role for emodin in DN (Tian et al., 2018). Chrysophanol also significantly slowed the progression of DN through the inactivation of TGF-β/EMT signaling *in vitro* and *in vivo* (Guo et al., 2017). Scopoletin, a naturally occurring hydroxycoumarin, acts as an antidiabetic agent, exhibiting anti-inflammatory and antioxidant activities and improving insulin resistance (Jang et al., 2018; Lee et al., 2021). *H. nipponica* Whitman plays a key role in treating diabetic vascular disease (Wang et al., 2021). 1-*O*-Octadecanoyl-sn-glycero-3-phosphocholine and 1-*O*-hexadecanoyl-*sn*-glycero-3-phosphocholine were lysophosphatidylcholines (LPCs). Some researchers have found that LPC has hypoglycemic effects. GPR119 is expressed on pancreatic beta cells and enhances insulin secretion, and researchers have discovered that LPC and its 2-Ome analog (2-Ome-LPC) can bind to GPR119 and stimulate insulin secretion (Drzazga et al., 2018a; Drzazga et al., 2018b). In brief, the screened compounds indicated a strong connection with DN.

Furthermore, the pharmacokinetic profiles of 156 components were analyzed. Based on the non-compartmental model analysis, active ingredients with faster absorption, longer half-life, higher peak concentration, and larger AUC values were considered screening criteria. Prototype compounds P1, P2, P11, P12, P13, P14, P20, P37, P41, and P45 and the metabolites M9, M26, M28, M35, M39, M52, M56, M57, M59, M85, M90, M94, and M99 had better pharmacokinetic behavior than other prototype compounds and metabolites. The potential active ingredients were mainly derived from prototype compounds of flavonoids and anthraquinones and metabolites resulting from their oxidation and methylation. Prototype compounds and metabolites of flavonoids and anthraquinones showed a fluctuation in blood concentration or double absorption peaks in serum concentration–time curves, which might result from enterohepatic circulation, reabsorption, and biotransformation (Song and Jiang, 2017). Flavonoids and anthraquinones were the two predominant classes of compounds in ZJTSD, which were absorbed into the blood in the form of prototype compounds and metabolites. Flavonoids play a multi-target and multi-pathway role in DN therapy, particularly through anti-oxidative stress and anti-inflammatory actions that are associated with apoptosis, glomerular protection, and renal fibrosis (Hu et al., 2021). A range of pathophysiological alterations are involved in the etiology of DN; persistently elevated blood glucose levels cause aberrant inflammatory responses and redox reactions in kidney tissue, resulting in glomerulosclerosis and tubulointerstitial fibrosis (Zhang et al., 2021; Wu et al., 2021). Previous studies indicated that anthraquinones caused improvements in renal functions in diabetic nephropathy rats (Li et al., 2017). Furthermore, existing research literature demonstrated that the active ingredients of rhubarb anthraquinones extracts can mediate multiple molecular mechanisms, particularly oxidative stress, and inflammation (Mohammed et al., 2020; Luo et al., 2021; Dey et al., 2022). Therefore, flavonoids and anthraquinones could be considered the potential active ingredients of Zhijun Tangshen Decoction. In brief, the differences reflected in the dynamic changes of these compounds *in vivo* could contribute to the prediction of which compounds may be potentially active. Moreover, it is essential to carry out further experiments for the investigation and validation of the exact activities of these compounds absorbed into the blood.

## 5 Conclusion

In this study, UPLC-Q/TOF-MS was first applied for the characterization of the chemical compound of ZJTSD. Subsequently, prototype compounds and metabolites were identified after gavage of ZJTSD in rats. Moreover, the multi-compound pharmacokinetics strategy combined with semi-quantitative methods presented the dynamic changes in the prototypical compounds and metabolites *in vivo* by UPLC-Q/TOF-MS. This work provided a dependable and appropriate strategy for the screening and discovery of potentially bioactive ingredients that promote the pharmacological effects of TCM, laying a foundation for further in-depth studies on the bioactive ingredients of ZJTSD.

## Data Availability

The datasets presented in this study can be found in online repositories. The names of the repository/repositories and accession number(s) can be found in the article/Supplementary Material.

## Abbreviation

ZJTSD, Zhijun Tangshen Decoction; BPCs, base peak chromatograms; AUC0→t, area under the concentration–time curve at the last experimental point; MRT0→t, mean residence time at the last experimental point; t1/2, the elimination half-life; T_max_, time for maximal concentration; DN, diabetic nephropathy; C_max_, the maximum concentration; IS, internal standard; TCM, traditional Chinese medicine; UPLC-Q/TOF-MS, ultra-high performance liquid chromatography coupled with quadrupole time-of-flight mass spectrometry; UPLC, ultra-high performance liquid chromatography; Q/TOF-MS, quadrupole time-of-flight mass spectrometry.

## References

[B1] Bakris G. L. Agarwal R. Anker S. D. Pitt B. Ruilope L. M. Rossing P. (2020). Effect of finerenone on chronic kidney disease outcomes in type 2 diabetes. N. Engl. J. Med. 383 (23), 2219–2229. 10.1056/NEJMoa2025845 33264825

[B2] Chen H. Zhu Y. Zhao X. Yang Z. (2023). Tingli Dazao Decoction pretreatment ameliorates mitochondrial damage induced by oxidative stress in cardiomyocytes. J. Ethnopharmacol. 303, 115987. 10.1016/j.jep.2022.115987 36455763

[B3] Chen J. Shen B. Jiang Z. (2022a). Traditional Chinese medicine prescription Shenling BaiZhu powder to treat ulcerative colitis: clinical evidence and potential mechanisms. Front. Pharmacol. 13, 978558. 10.3389/fphar.2022.978558 36160392 PMC9494158

[B4] Chen L. Cao H. Huang Q. Xiao J. Teng H. (2022b). Absorption, metabolism and bioavailability of flavonoids: a review. Crit. Rev. Food Sci. Nutr. 62 (28), 7730–7742. 10.1080/10408398.2021.1917508 34078189

[B5] Cui H. X. Zhang L. S. Luo Y. Yuan K. Huang Z. Y. Guo Y. (2019). A purified anthraquinone-glycoside preparation from rhubarb ameliorates type 2 diabetes mellitus by modulating the gut microbiota and reducing inflammation. Front. Microbiol. 10, 1423. 10.3389/fmicb.2019.01423 31293553 PMC6603233

[B6] Del Vecchio L. Beretta A. Jovane C. Peiti S. Genovesi S. (2021). A role for SGLT-2 inhibitors in treating non-diabetic chronic kidney disease. Drugs 81 (13), 1491–1511. 10.1007/s40265-021-01573-3 34363606

[B7] Dey P. Kundu A. Lee H. E. Kar B. Vishal V. Dash S. (2022). Molineria recurvata ameliorates streptozotocin-induced diabetic nephropathy through antioxidant and anti-inflammatory pathways. Molecules 27 (15), 4985. 10.3390/molecules27154985 35956936 PMC9370403

[B8] Dong X. Zeng Y. Liu Y. You L. Yin X. Fu J. (2020). Aloe-emodin: a review of its pharmacology, toxicity, and pharmacokinetics. Phytother. Res. 34 (2), 270–281. 10.1002/ptr.6532 31680350

[B9] Drzazga A. Kristinsson H. Sałaga M. Zatorski H. Koziołkiewicz M. Gendaszewska-Darmach E. (2018a). Lysophosphatidylcholine and its phosphorothioate analogues potentiate insulin secretion via GPR40 (FFAR1), GPR55 and GPR119 receptors in a different manner. Mol. Cell Endocrinol. 472, 117–125. 10.1016/j.mce.2017.12.002 29225068

[B10] Drzazga A. Sowińska A. Krzemińska A. Okruszek A. Paneth P. Koziołkiewicz M. (2018b). 2-OMe-lysophosphatidylcholine analogues are GPR119 ligands and activate insulin secretion from βTC-3 pancreatic cells: evaluation of structure-dependent biological activity. Biochim. Biophys. Acta Mol. Cell Biol. Lipids 1863 (1), 91–103. 10.1016/j.bbalip.2017.10.004 29079451

[B11] Du P. Guan Y. An Z. Li P. Liu L. (2019). A selective and robust UPLC-MS/MS method for the simultaneous quantitative determination of anlotinib, ceritinib and ibrutinib in rat plasma and its application to a pharmacokinetic study. Analyst 144 (18), 5462–5471. 10.1039/c9an00861f 31380858

[B12] Gao M. Xue X. Zhang X. Chang Y. Zhang Q. Li X. (2022). Discovery of potential active ingredients of Er-Zhi-Wan, a famous traditional Chinese formulation, in model rat serum for treating osteoporosis with kidney-yin deficiency by UPLC-Q/TOF-MS and molecular docking. J. Chromatogr. B Anal. Technol. Biomed. Life Sci. 1208, 123397. 10.1016/j.jchromb.2022.123397 35921699

[B13] Garofalo C. Borrelli S. Liberti M. E. Andreucci M. Conte G. Minutolo R. (2019). SGLT2 inhibitors: nephroprotective efficacy and side effects. Med. Kaunas. 55 (6), 268. 10.3390/medicina55060268 PMC6630922 31212638

[B14] Gong G. W. Tang W. H. Zhou Z. Jiang Y. W. Wang C. Z. Cheng H. (2022). Potential efficacious materials investigation of Yi-Yi Mixture based on Metabolome-oriented network pharmacology strategy. J. Chromatogr. B Anal. Technol. Biomed. Life Sci. 1197, 123199. 10.1016/j.jchromb.2022.123199 35305386

[B15] Guo J. Chen H. Zhao X. Zhao L. Tong X. (2017). Diabetic kidney disease treated with a modified Shenzhuo formula derived from Traditional Chinese Medicine: a case report. J. Tradit. Chin. Med. 37 (6), 854–861. 10.1016/s0254-6272(18)30051-7 32188197

[B16] He L. Liu Y. Yang K. Zou Z. Fan C. Yao Z. (2021). The discovery of Q-markers of Qiliqiangxin Capsule, a traditional Chinese medicine prescription in the treatment of chronic heart failure, based on a novel strategy of multi-dimensional "radar chart" mode evaluation. Phytomedicine 82, 153443. 10.1016/j.phymed.2020.153443 33429210

[B17] He Y. Zhou Z. Li W. Zhang Y. Shi R. Li T. (2022). Metabolic profiling and pharmacokinetic studies of Baihu-Guizhi decoction in rats by UFLC-Q-TOF-MS/MS and UHPLC-Q-TRAP-MS/MS. Chin. Med. 17 (1), 117. 10.1186/s13020-022-00665-w 36195951 PMC9531372

[B18] Hu Q. Qu C. Xiao X. Zhang W. Jiang Y. Wu Z. (2021). Flavonoids on diabetic nephropathy: advances and therapeutic opportunities. Chin. Med. 16 (1), 74. 10.1186/s13020-021-00485-4 34364389 PMC8349014

[B19] Huang Q. Zhang F. Liu S. Jiang Y. Ouyang D. (2021). Systematic investigation of the pharmacological mechanism for renal protection by the leaves of Eucommia ulmoides Oliver using UPLC-Q-TOF/MS combined with network pharmacology analysis. Biomed. Pharmacother. 140, 111735. 10.1016/j.biopha.2021.111735 34020251

[B20] Huo X. Gu Y. Zhang Y. (2022). The discovery of multi-target compounds with anti-inflammation activity from traditional Chinese medicine by TCM-target effects relationship spectrum. J. Ethnopharmacol. 293, 115289. 10.1016/j.jep.2022.115289 35427724

[B21] Jang J. H. Park J. E. Han J. S. (2018). Scopoletin inhibits α-glucosidase *in vitro* and alleviates postprandial hyperglycemia in mice with diabetes. Eur. J. Pharmacol. 834, 152–156. 10.1016/j.ejphar.2018.07.032 30031794

[B22] Lee E. J. Na W. Kang M. K. Kim Y. H. Kim D. Y. Oh H. (2021). Hydroxycoumarin scopoletin inhibits bone loss through enhancing induction of bone turnover markers in a mouse model of type 2 diabetes. Biomedicines 9 (6), 648. 10.3390/biomedicines9060648 34200167 PMC8227109

[B23] Li C. Jia W. W. Yang J. L. Cheng C. Olaleye O. E. (2022a). Multi-compound and drug-combination pharmacokinetic research on Chinese herbal medicines. Acta Pharmacol. Sin. 43 (12), 3080–3095. 10.1038/s41401-022-00983-7 36114271 PMC9483253

[B24] Li C. Wang J. Wang Y. Gao H. Wei G. Huang Y. (2019). Recent progress in drug delivery. Acta Pharm. Sin. B 9 (6), 1145–1162. 10.1016/j.apsb.2019.08.003 31867161 PMC6900554

[B25] Li J. Ma J. Li Q. Fan S. Fan L. Ma H. (2021). Determination of 35 free amino acids in tea using ultra-performance liquid chromatography coupled with quadrupole time-of-flight mass spectrometry. Front. Nutr. 8, 767801. 10.3389/fnut.2021.767801 34957181 PMC8697017

[B26] Li P. Lu Q. Jiang W. Pei X. Sun Y. Hao H. (2017a). Pharmacokinetics and pharmacodynamics of rhubarb anthraquinones extract in normal and disease rats. Biomed. Pharmacother. 91, 425–435. 10.1016/j.biopha.2017.04.109 28475921

[B27] Li Y. Peng Y. Wang M. Tu P. Li X. (2017b). Human gastrointestinal metabolism of the cistanches herba water extract *in vitro*: elucidation of the metabolic profile based on comprehensive metabolite identification in gastric juice, intestinal juice, human intestinal bacteria, and intestinal microsomes. J. Agric. Food Chem. 65 (34), 7447–7456. 10.1021/acs.jafc.7b02829 28771352

[B28] Li Z. Deng H. Guo X. Yan S. Lu C. Zhao Z. (2022b). Effective dose/duration of natural flavonoid quercetin for treatment of diabetic nephropathy: a systematic review and meta-analysis of rodent data. Phytomedicine 105, 154348. 10.1016/j.phymed.2022.154348 35908521

[B29] Liang J. Xu F. Zhang Y. Z. Huang S. Zang X. Y. Zhao X. (2013). The profiling and identification of the absorbed constituents and metabolites of Paeoniae Radix Rubra decoction in rat plasma and urine by the HPLC-DAD-ESI-IT-TOF-MS(n) technique: a novel strategy for the systematic screening and identification of absorbed constituents and metabolites from traditional Chinese medicines. J. Pharm. Biomed. Anal. 83, 108–121. 10.1016/j.jpba.2013.04.029 23727363

[B30] Lin P. Dai Y. Yao Z. Qin Z. He L. Wang Q. (2019). Metabolic profiles and pharmacokinetics of Qingre Xiaoyanning capsule, a traditional Chinese medicine prescription of Sarcandrae Herba, in rats by UHPLC coupled with quadrupole time-of-flight tandem mass spectrometry. J. Sep. Sci. 42 (4), 784–796. 10.1002/jssc.201800981 30511805

[B31] Liu W. He H. Li Z. Zhou Q. Zhou B. Li Z. F. (2023). Analysis of chemical constituents of Sabia parviflora by ultrahigh performance liquid chromatography quadrupole time of flight tandem mass spectrometry. J. Chromatogr. A 1687, 463650. 10.1016/j.chroma.2022.463650 36462476

[B32] Luo L. P. Suo P. Ren L. L. Liu H. J. Zhang Y. Zhao Y. Y. (2021). Shenkang injection and its three anthraquinones ameliorates renal fibrosis by simultaneous targeting IƙB/NF-ƙB and keap1/nrf2 signaling pathways. Front. Pharmacol. 12, 800522. 10.3389/fphar.2021.800522 35002735 PMC8729217

[B33] Mohammed A. Ibrahim M. A. Tajuddeen N. Aliyu A. B. Isah M. B. (2020). Antidiabetic potential of anthraquinones: a review. Phytother. Res. 34 (3), 486–504. 10.1002/ptr.6544 31773816

[B34] Naeem A. Ming Y. Pengyi H. Jie K. Y. Yali L. Haiyan Z. (2022). The fate of flavonoids after oral administration: a comprehensive overview of its bioavailability. Crit. Rev. Food Sci. Nutr. 62 (22), 6169–6186. 10.1080/10408398.2021.1898333 33847202

[B35] Ning Z. W. Zhai L. X. Huang T. Peng J. Hu D. Xiao H. T. (2019). Identification of α-glucosidase inhibitors from cyclocarya paliurus tea leaves using UF-UPLC-Q/TOF-MS/MS and molecular docking. Food Funct. 10 (4), 1893–1902. 10.1039/c8fo01845f 30865735

[B36] Roberts M. S. Magnusson B. M. Burczynski F. J. Weiss M. (2002). Enterohepatic circulation: physiological, pharmacokinetic and clinical implications. Clin. Pharmacokinet. 41 (10), 751–790. 10.2165/00003088-200241100-00005 12162761

[B37] Sang Q. Jia Q. Zhang H. Lin C. Zhao X. Zhang M. (2021). Chemical profiling and quality evaluation of Zhishi-Xiebai-Guizhi Decoction by UPLC-Q-TOF-MS and UPLC fingerprint. J. Pharm. Biomed. Anal. 194, 113771. 10.1016/j.jpba.2020.113771 33280997

[B38] Selby N. M. Taal M. W. (2020). An updated overview of diabetic nephropathy: diagnosis, prognosis, treatment goals and latest guidelines. Diabetes Obes. Metab. 22 (Suppl. 1), 3–15. 10.1111/dom.14007 32267079

[B39] Shen Z. Cui T. Liu Y. Wu S. Han C. Li J. (2023). Astragalus membranaceus and Salvia miltiorrhiza ameliorate diabetic kidney disease via the "gut-kidney axis. Phytomedicine 121, 155129. 10.1016/j.phymed.2023.155129 37804821

[B40] Song D. X. Jiang J. G. (2017). Hypolipidemic components from medicine Food homology species used in China: pharmacological and health effects. Arch. Med. Res. 48 (7), 569–581. 10.1016/j.arcmed.2018.01.004 29452699

[B41] Tan T. Luo Y. Zhong C. C. Xu X. Feng Y. (2017). Comprehensive profiling and characterization of coumarins from roots, stems, leaves, branches, and seeds of Chimonanthus nitensOliv. using ultra-performance liquid chromatography/quadrupole-time-of-flight mass spectrometry combined with modified mass defect filter. J. Pharm. Biomed. Anal. 141, 140–148. 10.1016/j.jpba.2017.04.016 28445814

[B42] Tang Y. P. Xu D. Q. Yue S. J. Chen Y. Y. Fu R. J. Bai X. (2022). Modern research thoughts and methods on bio-active components of TCM formulae. Chin. J. Nat. Med. 20 (7), 481–493. 10.1016/s1875-5364(22)60206-1 35907647

[B43] Tian N. Gao Y. Wang X. Wu X. Zou D. Zhu Z. (2018). Emodin mitigates podocytes apoptosis induced by endoplasmic reticulum stress through the inhibition of the PERK pathway in diabetic nephropathy. Drug Des. Devel Ther. 12, 2195–2211. 10.2147/dddt.S167405 PMC6047613 30034224

[B44] Wang F. Huang S. Chen Q. Hu Z. Li Z. Zheng P. (2020). Chemical characterisation and quantification of the major constituents in the Chinese herbal formula Jian-Pi-Yi-Shen pill by UPLC-Q-TOF-MS/MS and HPLC-QQQ-MS/MS. Phytochem. Anal. 31 (6), 915–929. 10.1002/pca.2963 32488993

[B45] Wang J. S. Feng J. L. Li X. Chen Z. L. Bao B. H. Deng S. (2021). Effect of leech-centipede medicine on improving erectile function in diabetes-induced erectile dysfunction rats via PDE5 signalling pathway-related molecules. Pharm. Biol. 59 (1), 167–174. 10.1080/13880209.2021.1878237 33569974 PMC7889219

[B46] Wang S. Q. Sheng M. X. Zhang C. P. Yan X. L. Wu L. X. (2019). Observation on the therapeutic effect of method of clearing collaterals, reducing turbidity and tonifying kidney in treating patients with diabetic nephropathy in the early and middle stages and its effect on inflammatory factors in serum. J. Chin. Med. Mater. 42 (04), 920–923. 10.13863/j.issn1001-4454.2019.04.045

[B47] Wang X. Sun H. Zhang A. Jiao G. Sun W. Yuan Y. (2011). Pharmacokinetics screening for multi-components absorbed in the rat plasma after oral administration traditional Chinese medicine formula Yin-Chen-Hao-Tang by ultra performance liquid chromatography-electrospray ionization/quadrupole-time-of-flight mass spectrometry combined with pattern recognition methods. Analyst 136 (23), 5068–5076. 10.1039/c1an15752c 21991580

[B48] Wei W. L. An Y. L. Li Z. W. Wang Y. Y. Ji H. J. Hou J. J. (2019). Simultaneous determination of resibufogenin and its eight metabolites in rat plasma by LC-MS/MS for metabolic profiles and pharmacokinetic study. Phytomedicine 60, 152971. 10.1016/j.phymed.2019.152971 31178234

[B49] Wu D. Chen Q. Chen X. Han F. Chen Z. Wang Y. (2023). The blood-brain barrier: structure, regulation, and drug delivery. Signal Transduct. Target Ther. 8 (1), 217. 10.1038/s41392-023-01481-w 37231000 PMC10212980

[B50] Wu L. X. (1999). Treatment of diabetic nephropathy with 64 cases of diabetic nephropathy by self-proposed Zhijun Tangshen Decotion. J. Nanjing Univ. Traditional Chin. Med. 15 (4), 253.

[B51] Wu X. Li H. Wan Z. Wang R. Liu J. Liu Q. (2021). The combination of ursolic acid and empagliflozin relieves diabetic nephropathy by reducing inflammation, oxidative stress and renal fibrosis. Biomed. Pharmacother. 144, 112267. 10.1016/j.biopha.2021.112267 34624679

[B52] Yin J. Zhang X. Zhang Y. Ma Y. Li L. Li D. (2019). Comprehensive study of the *in vivo* and *in vitro* metabolism of dietary isoflavone biochanin A based on UHPLC-Q-TOF-MS/MS. J. Agric. Food Chem. 67 (45), 12481–12495. 10.1021/acs.jafc.9b05776 31630515

[B53] Zeng J. Y. Wang Y. Miao M. Bao X. R. (2021). The effects of rhubarb for the treatment of diabetic nephropathy in animals: a systematic review and meta-analysis. Front. Pharmacol. 12, 602816. 10.3389/fphar.2021.602816 34177560 PMC8226322

[B54] Zhang A. Xu Q. Jiang J. Zhao Z. Zhang L. Tao K. (2023). Qualitative and quantitative determination of chemical constituents in Jinbei oral liquid, a modern Chinese medicine for coronavirus disease 2019, by ultra-performance liquid chromatography coupled with mass spectrometry. Front. Chem. 11, 1079288. 10.3389/fchem.2023.1079288 36825225 PMC9941701

[B55] Zhang D. Wei C. Hop C. Wright M. R. Hu M. Lai Y. (2021a). Intestinal excretion, intestinal recirculation, and renal tubule reabsorption are underappreciated mechanisms that drive the distribution and pharmacokinetic behavior of small molecule drugs. J. Med. Chem. 64 (11), 7045–7059. 10.1021/acs.jmedchem.0c01720 34010555

[B56] Zhang L. Jing M. Liu Q. (2021b). Crocin alleviates the inflammation and oxidative stress responses associated with diabetic nephropathy in rats via NLRP3 inflammasomes. Life Sci. 278, 119542. 10.1016/j.lfs.2021.119542 33915128

[B57] Zhang L. Zuo Z. Lin G. (2007). Intestinal and hepatic glucuronidation of flavonoids. Mol. Pharm. 4 (6), 833–845. 10.1021/mp700077z 17979245

[B58] Zhang X. Liu Y. Wu H. Cao S. Wu P. Zhou A. (2020). Identification of major bioactive components and metabolites of Gandou decoction in rat urine by an integrative approach based on UPLC-Q-TOF-MS∼E coupled with xenometabolomics analytical platform. Acta Pharm. Sin. 55 (5), 971–978.

[B59] Zhang Y. Z. Xu F. Dong J. Liang J. Hashi Y. Shang M. Y. (2012). Profiling and identification of the metabolites of calycosin in rat hepatic 9000×g supernatant incubation system and the metabolites of calycosin-7-O-β-D-glucoside in rat urine by HPLC-DAD-ESI-IT-TOF-MS(n) technique. J. Pharm. Biomed. Anal. 70, 425–439. 10.1016/j.jpba.2012.06.006 22766358

[B60] Zhao G. Zhao W. Han L. Ding J. Chang Y. (2020). Metabolomics analysis of sea cucumber (Apostichopus japonicus) in different geographical origins using UPLC-Q-TOF/MS. Food Chem. 333, 127453. 10.1016/j.foodchem.2020.127453 32659664

[B61] Zhao J. Yang J. Xie Y. (2019). Improvement strategies for the oral bioavailability of poorly water-soluble flavonoids: an overview. Int. J. Pharm. 570, 118642. 10.1016/j.ijpharm.2019.118642 31446024

[B62] Zhong M. Tian X. Chen S. Chen M. Guo Z. Zhang M. (2019). Identifying the active components of Baihe-Zhimu decoction that ameliorate depressive disease by an effective integrated strategy: a systemic pharmacokinetics study combined with classical depression model tests. Chin. Med. 14, 37. 10.1186/s13020-019-0254-9 31572489 PMC6757420

[B63] Zhou M. Huo J. Wang C. Wang W. (2021). UPLC/Q-TOF MS screening and identification of antibacterial compounds in Forsythia suspensa (thunb.) Vahl leaves. Front. Pharmacol. 12, 704260. 10.3389/fphar.2021.704260 35153732 PMC8831367

