# Supplementary material for: Integrated chemical characterization, metabolite profiling, and pharmacokinetics analysis of Zhijun Tangshen Decoction by UPLC-Q/TOF-MS
Source: Front Pharmacol. 2024 Mar 8;15:1363678. doi: 10.3389/fphar.2024.1363678 (PMC10957775; doi:10.3389/fphar.2024.1363678)
Supplement: Supplementary file 1 [file DataSheet1.docx]

Supplementary Material

**Integrated chemical characterization, metabolite profiling and pharmacokinetics analysis of Zhijun Tangshen Decoction by UPLC-QTOF-MS**

**Qingheng Tong^1 †^, Yueyue Chang^1 †^,** **Guanxiong Shang^1^,** **Jiu Yin^1^, Xiaoqi Zhou^1^,** **Suwei Wang^2^, Xiaofeng Yan^2^,** **Fangfang Zhang^2^, Suqin Wang^2,^ *, Weifeng Yao^1,^ ***

^1^Jiangsu Collaborative Innovation Center of Chinese Medicinal Resources Industrialization, National and Local Collaborative Engineering Center of Chinese Medicinal Resources Industrialization and Formulae Innovative Medicine, School of Pharmacy, Nanjing University of Chinese Medicine, Nanjing, 210023, China

^2^Huai’an TCM Hospital Affiliated to Nanjing University of Chinese Medicine

Qingheng Tong^a^ ^†^ and Yueyue Chang^a^ ^†^ These authors contributed equally to this work and share first authorship.

***** **Corresponding author:**

Suqin Wang
fsyy02067@njucm.edu.cn

Weifeng Yao
yaowf@njucm.edu.cn


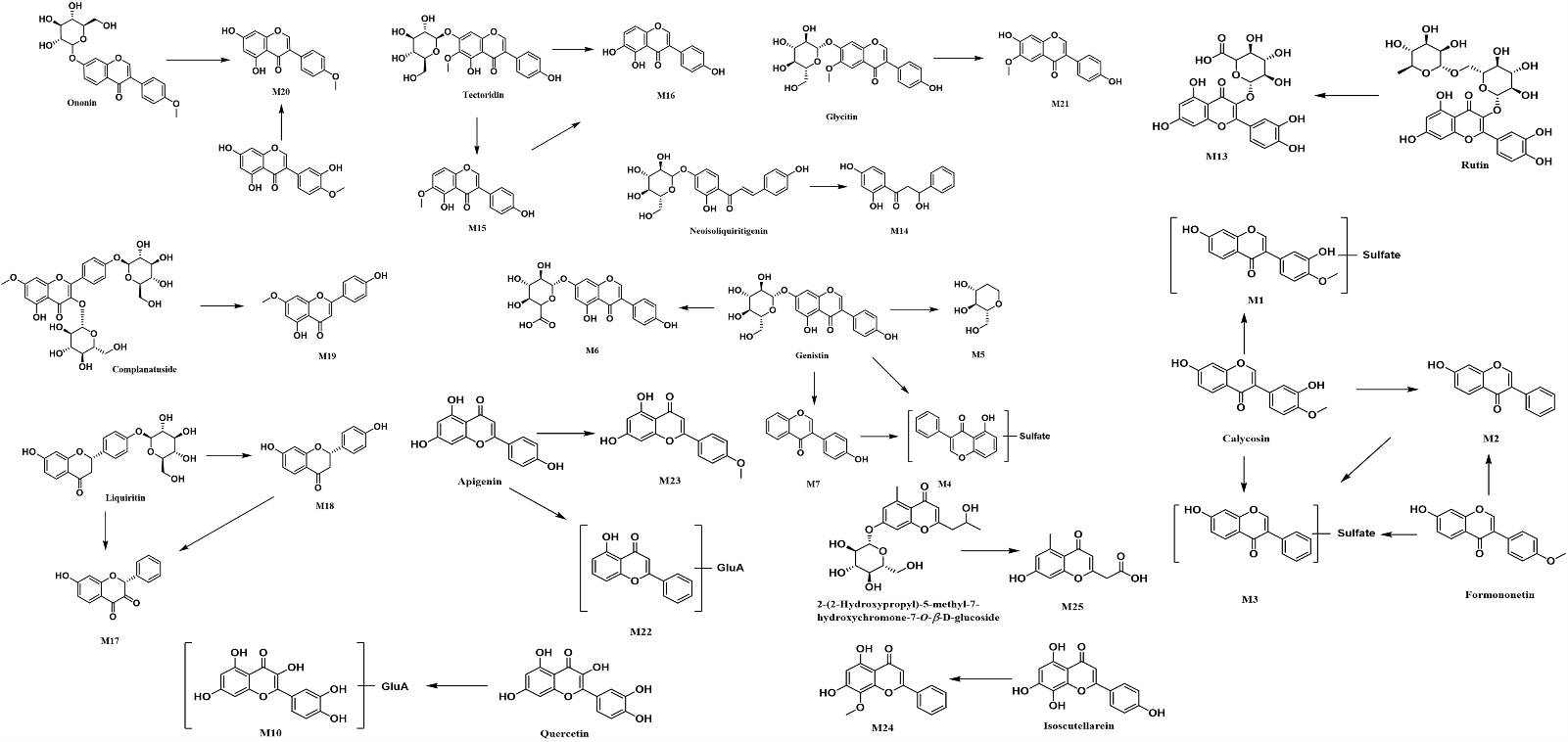


**Figure S1** The proposed metabolic pathway of flavonoids.


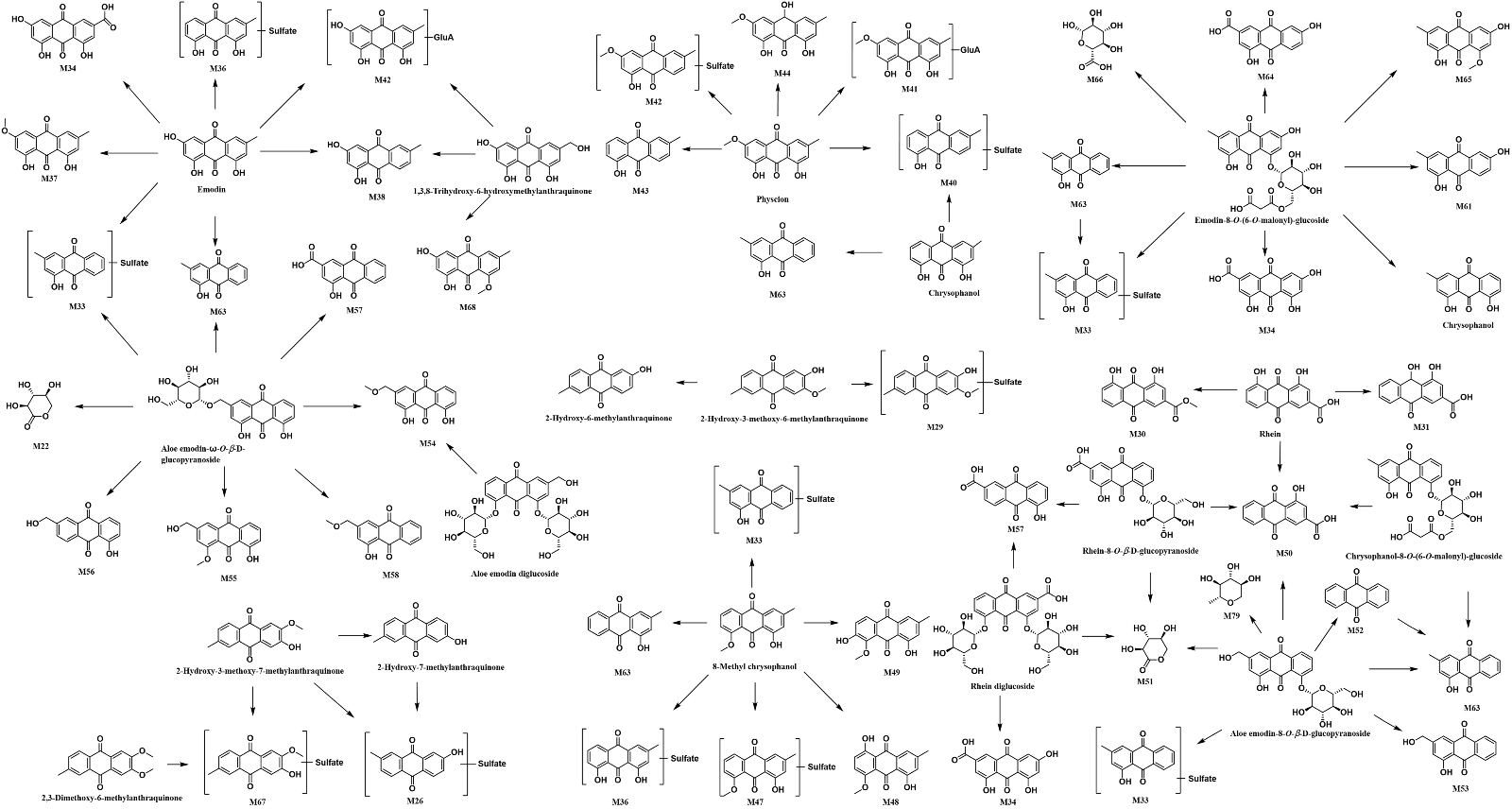


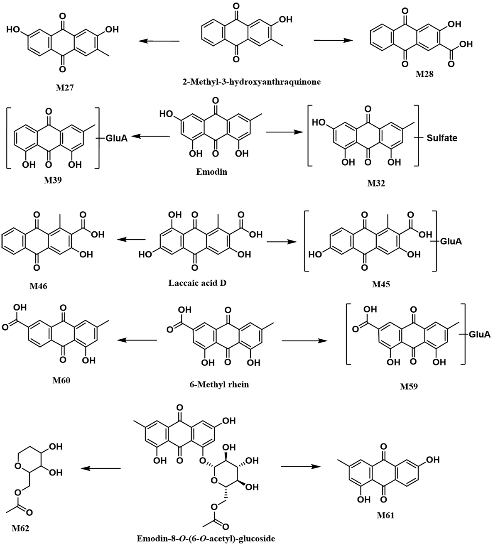

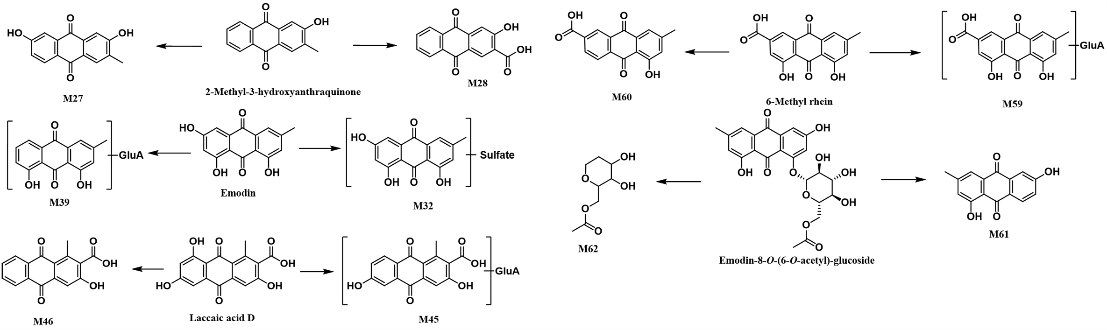


**Figure S2** The proposed metabolic pathway of anthraquinone.


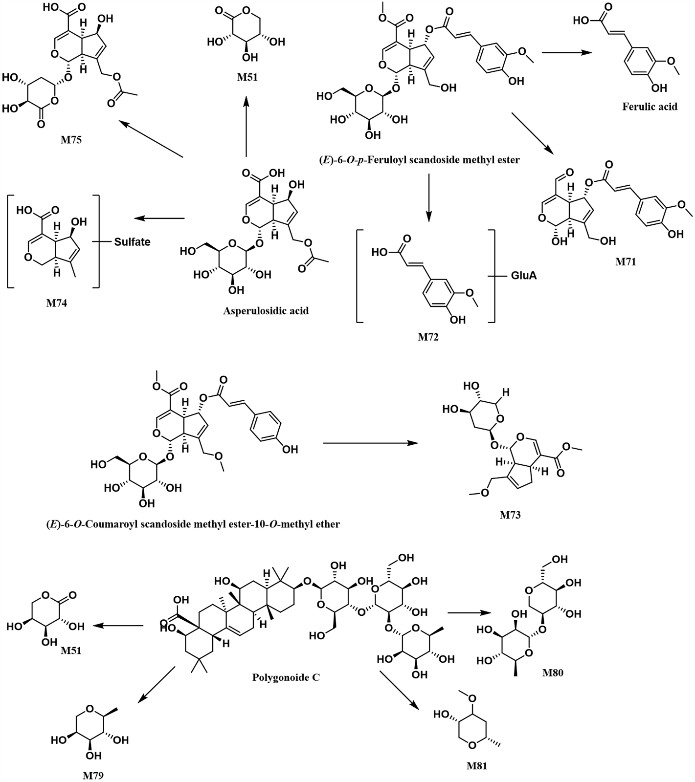


**Figure S3** The proposed metabolic pathway of terpenoids.


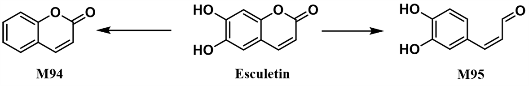


**Figure S4** The proposed metabolic pathway of phenylpropanoids.


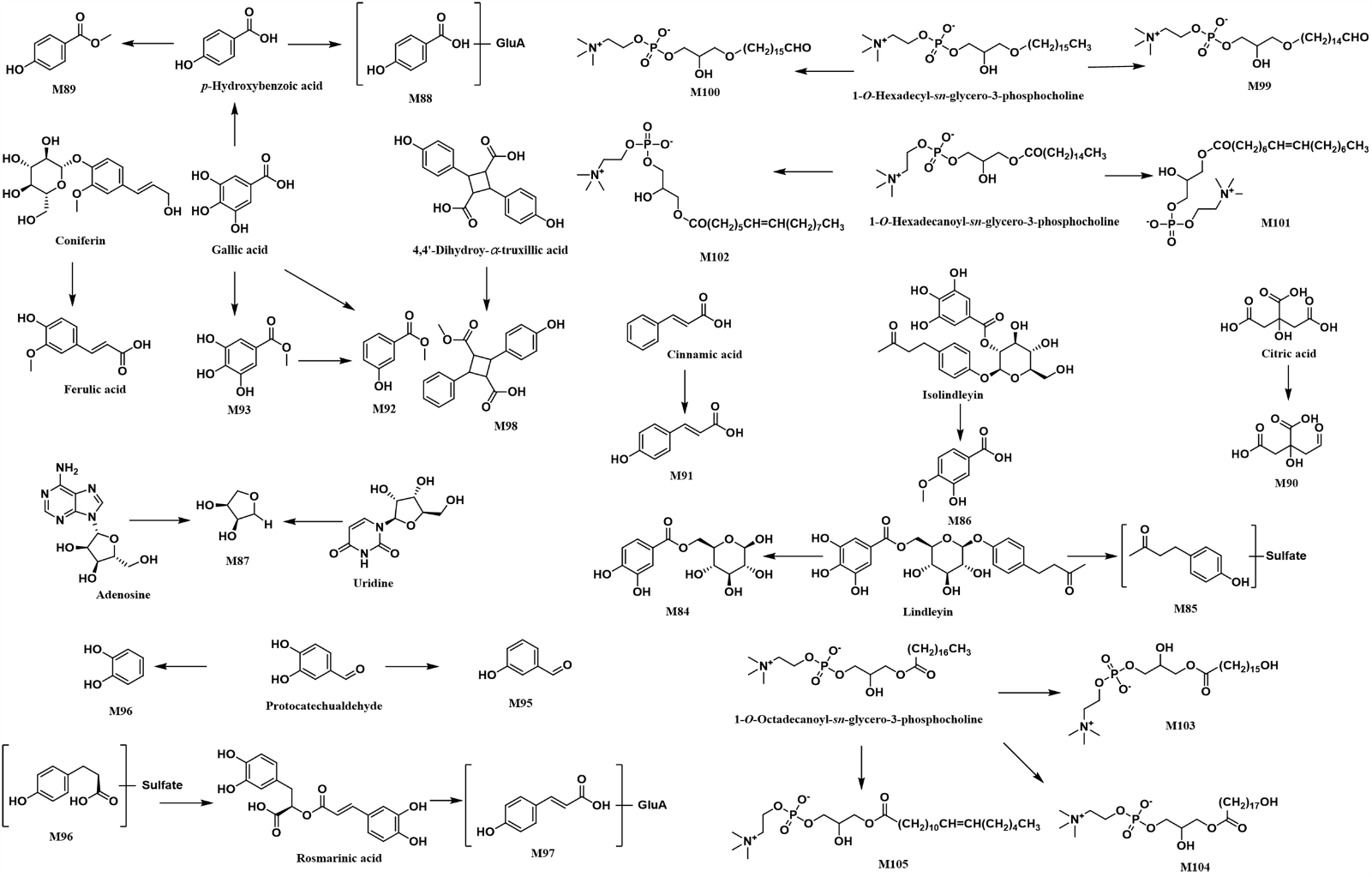


**Figure S5** The proposed metabolic pathway of other categories.


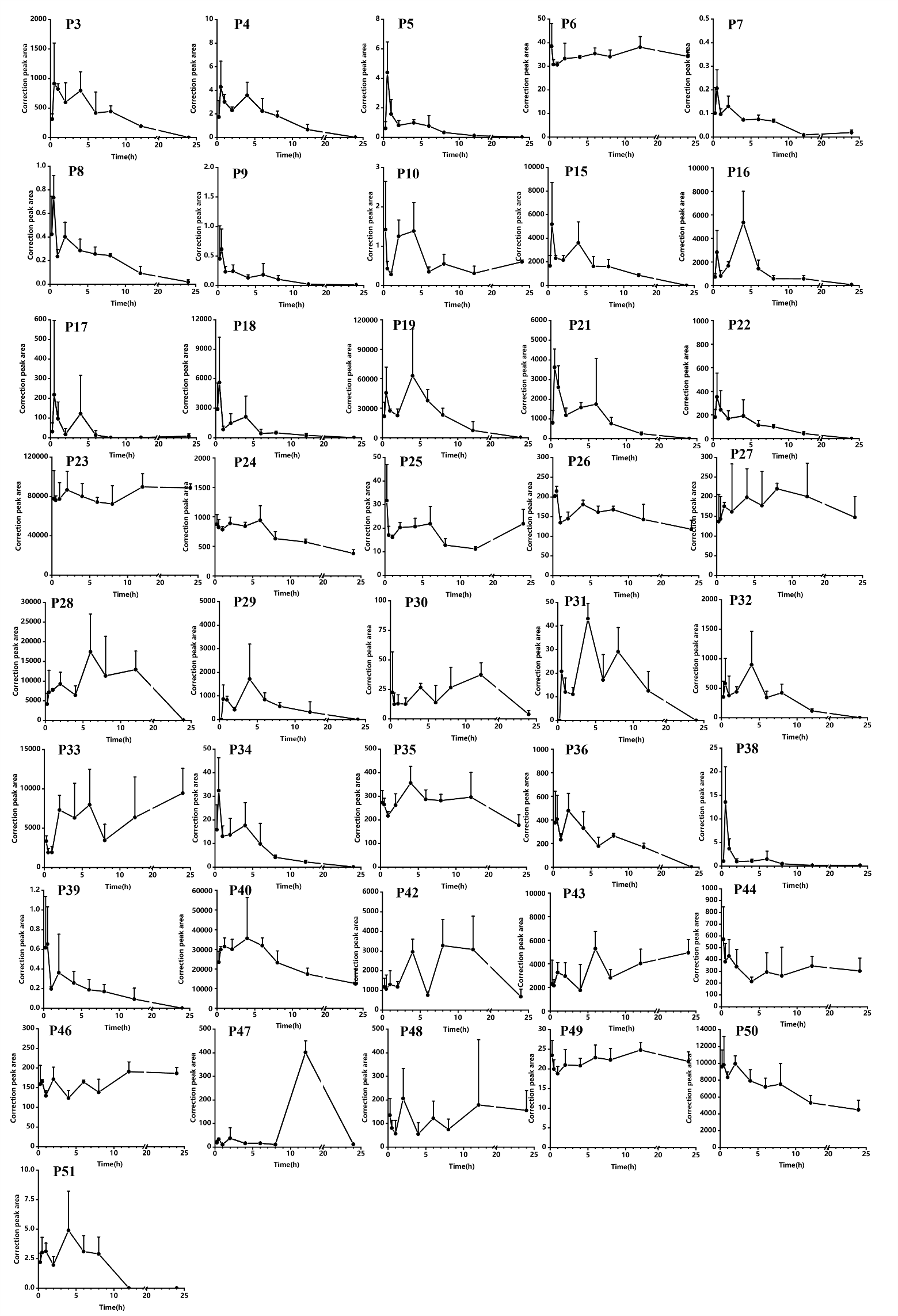


**Figure S6** Correction peak area-time curves for prototype compounds of ZJTSD absorbed into rat serum.


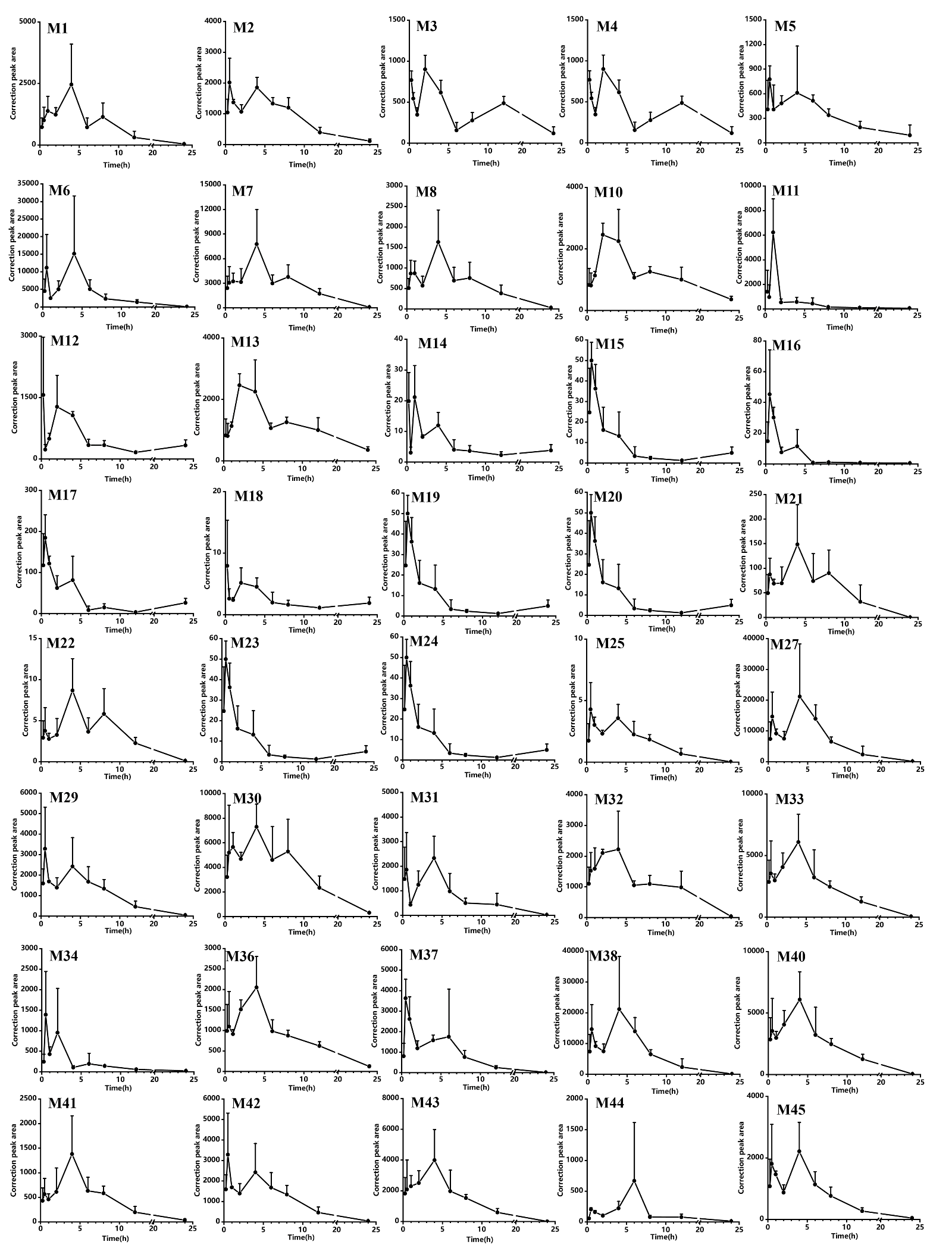


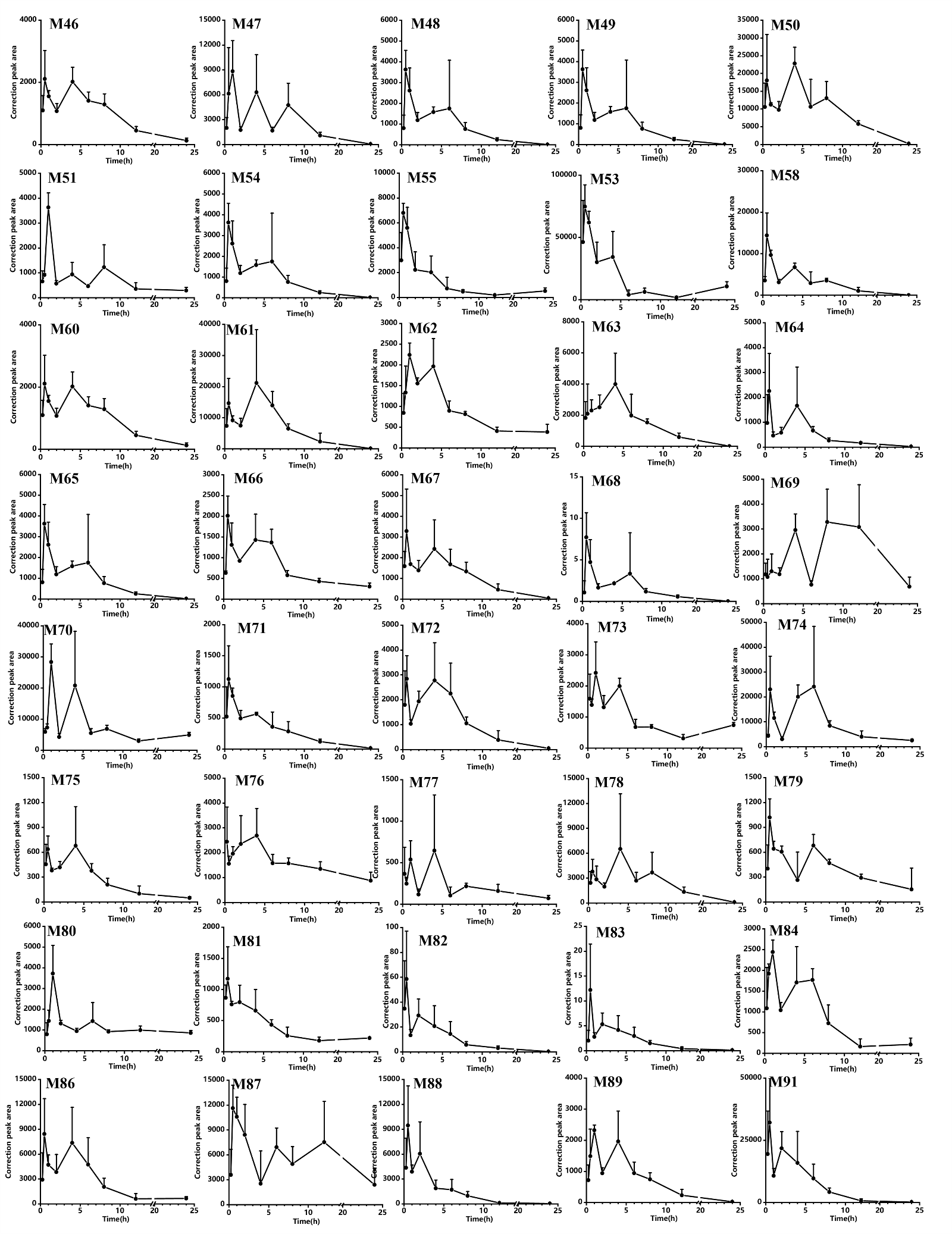


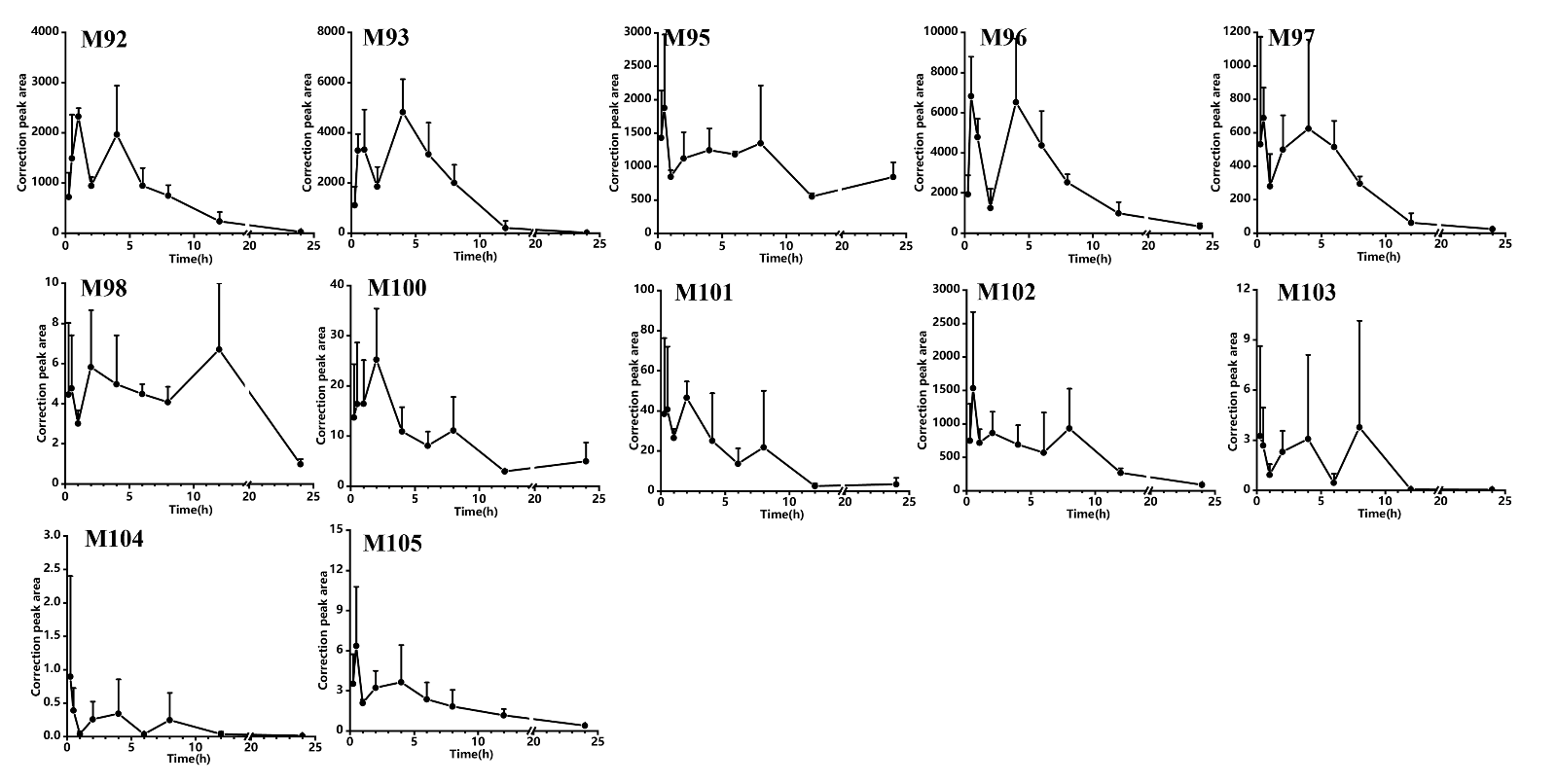


**Figure S7** Correction peak area-time curves for metabolites of ZJTSD in serum.

**Table S1** The mass spectral information of the compounds detected in ZJTSD.

| **No** | **Component Name** | **Retention Time (min)** | **Adduct** | **Formula** | **Found at Mass** | **Error**  **(ppm)** | **Category** | **Source** | **Fragments** |
| --- | --- | --- | --- | --- | --- | --- | --- | --- | --- |
| 1 | 2,5-Dimethyl-7-hydroxychromone | 6.38 | [M-H]^-^ | C_11_H_10_O_3_ | 189.0554 | -1.7 | Flavonoids | RP | 189.0571，146.0383，159.0453，105.0353，174.0324，147.0456 |
| 2 | 2-Methyl-5-acetonyl-7-hydroxychromone | 5.6 | [M-H]^-^ | C_13_H_12_O_4_ | 231.0662 | -0.3 | Flavonoids | RP | 189.0564，231.0673，188.0481 |
| 3 | 2-Methyl-5-carboxymethyl-7-hydroxyc hromone | 5.13 | [M-H]^-^ | C_12_H_10_O_5_ | 233.0449 | -2.8 | Flavonoids | RP | 189.0570，147.0456 |
| 4 | 7-Hydroxy-2-(2-hydroxypropyl)-5-methylchromen-4-one | 5.67 | [M-H]^-^ | C_13_H_14_O_4_ | 233.0814 | -2.3 | Flavonoids | RP | 189.0569，233.0828，187.0401，188.0485，159.0453，105.0353，149.0248 |
| 5 | Formononetin | 6.16 | [M-H]^-^ | C_16_H_12_O_4_ | 267.0660 | -1.0 | Flavonoids | AM | 252.0433，223.0405，267.0665，251.0353，195.0455 |
| 6 | Genistein | 6.1 | [M-H]^-^ | C_15_H_10_O_5_ | 269.0454 | -0.6 | Flavonoids | AM | 269.0473，240.0435，241.0504，225.0548 |
| 7 | Naringenin | 5.66 | [M-H]^-^ | C_15_H_12_O_5_ | 271.0600 | -4.4 | Flavonoids | AM | 119.0511，151.0042，155.0330，271.0577 |
| 8 | 7,4'-Dihydroxy-3'-methoxyisoflavone *or* Calycosin | 5.01 | [M-H]^-^ | C_16_H_12_O_5_ | 283.0609 | -1.1 | Flavonoids | AM | 268.0392，211.0403，283.0620，239.0361，240.0428，135.0094 |
| 9 | (*Z*)-2',5'-dihydroxy-6-methoxyaurone | 5.673 | [M-H]^-^ | C_16_H_12_O_5_ | 283.0610 | -0.7 | Flavonoids | AM | 268.0393，211.0408，283.0627，239.0364，240.0436 |
| 10 | 3-Methoxy-5,7-dihydroxyflavone | 7.05 | [M-H]^-^ | C_16_H_12_O_5_ | 283.0613 | 0.4 | Flavonoids | HD | 268.0385，239.0362，283.0620 |
| 11 | (6aR,11aR)-10-Hydroxy-3,9-dimethoxypterane | 6.89 | [M-H]^-^ | C_17_H_16_O_5_ | 299.0921 | -1.3 | Flavonoids | PS | 269.0468，284.0699，241.0513，255.0319，299.0926 |
| 12 | Quercetin^S^ | 6.97 | [M-H]^-^ | C_15_H_10_O_7_ | 301.0351 | -0.9 | Flavonoids | HD/AM | 151.0044，301.0361，121.0300，107.0141，178.9994 |
| 13 | Isomucronulatol | 6.57 | [M-H]^-^ | C_17_H_18_O_5_ | 301.1072 | -3.2 | Flavonoids | AM | 271.0619，301.1069，135.0452，121.0296，286.0847 |
| 14 | Odoratin | 10.04 | [M-H]^-^ | C_19_H_22_O_4_ | 313.1449 | 1.2 | Flavonoids | AM | 213.1200，269.1583，313.1483 |
| 15 | Aloesone-7-*O*-*β*-D-glucopyranoside | 5.85 | [M-H]^-^ | C_19_H_22_O_9_ | 393.1186 | -1.3 | Flavonoids | RP | 231.0675 |
| 16 | Kaempferol-3-*O*-glucorhamnoside | 5.41 | [M-H]^-^ | C_21_H_20_O_10_ | 431.0974 | -2.3 | Flavonoids | RP | 269.0463，431.0971，225.0575，431.1124，295.0733 |
| 17 | Apigenin-7-*O*-*β*-D-glucoside | 5.94 | [M-H]^-^ | C_21_H_20_O_10_ | 431.0978 | -1.3 | Flavonoids | PS | 269.0463，431.1023 |
| 18 | Genistin | 5.63 | [M-H]^-^ | C_21_H_20_O_10_ | 431.0983 | -0.2 | Flavonoids | AM | 269.0471，240.0434 |
| 19 | Kaempferol-3-O-β-D-glucopyranoside | 4.94 | [M-H]^-^ | C_21_H_20_O_11_ | 447.0928 | -1.1 | Flavonoids | HD | 285.0413，447.0934 |
| 20 | Isoquercitrin | 5.31 | [M-H]^-^ | C_21_H_20_O_12_ | 463.0876 | -1.3 | Flavonoids | AM | 300.0291，463.0883，301.0356 |
| 21 | 2',7-Dihydroxy-3',4'-dimethoxyisoflavanoside | 6.56 | [M-H]^-^ | C_23_H_28_O_10_ | 463.1608 | -0.4 | Flavonoids | PS | 301.1092，121.0303，254.0593 |
| 22 | Rheinoside C | 6.04 | [M-H]^-^ | C_27_H_30_O_15_ | 593.1504 | -1.3 | Flavonoids | RP | 593.1528，269.0466 |
| 23 | Rutin^S^ | 5.08 | [M-H]^-^ | C_27_H_30_O_16_ | 609.1466 | 0.8 | Flavonoids | HD | 609.1507，300.0299，301.0369 |
| 24 | Kaempferol-3-*O*-[2”-*O*-(*E*-6”’-*O*-caffeoyl)-*β*-D-glucopyranosyl]-*β*-D-galactopyranoside | 5.75 | [M-H]^-^ | C_37_H_38_O_19_ | 785.1935 | 0.1 | Flavonoids | HD | 785.1953，284.0327，609.1464，285.0408 |
| 25 | 2,5-Dimethyl-7-methoxychromone | 12.14 | [M+H]^+^ | C_12_H_12_O_3_ | 205.0854 | -2.5 | Flavonoids | RP | 149.0229，121.0277 |
| 26 | Isoliquiritigenin *or* Liquiritigenin | 5.2 | [M+H]^+^ | C_15_H_12_O_4_ | 257.0800 | -3.3 | Flavonoids | PS/AM | 137.0233，257.0837，147.0438 |
| 27 | Apigenin | 8.99 | [M+H]^+^ | C_15_H_10_O_5_ | 271.059 | -4.1 | Flavonoids | HD | 271.0597 |
| 28 | Isoscutellarein | 6.99 | [M+H]^+^ | C_15_H_10_O_6_ | 287.0543 | -2.5 | Flavonoids | HD | 241.0494，287.0552，269.0448，257.0444，167.0488 |
| 29 | Pratensein | 4.59 | [M+H]^+^ | C_16_H_12_O_6_ | 301.0699 | -2.5 | Flavonoids | AM | 301.0708，213.0544，269.0442，241.0492，137.0229 |
| 30 | Rhamnocitrin | 6.58 | [M+H]^+^ | C_16_H_12_O_6_ | 301.0707 | 0.1 | Flavonoids | AM | 301.0716，167.0698，301.0933，283.0521 |
| 31 | Methylnissolin | 6.42 | [M+H]^+^ | C_17_H_16_O_5_ | 301.1068 | -0.8 | Flavonoids | AM | 167.0702，134.0359，152.0465，301.1070，106.0410 |
| 32 | 2',4',5',5,7-Pentahydroxy flavone | 6 | [M+H]^+^ | C_15_H_10_O_7_ | 303.0496 | -1.1 | Flavonoids | HD | 303.0491，153.0167，137.0228 |
| 33 | 2-(2-Hydroxypropyl)-5-methyl-7-hydroxychromone-7-*O*-*β*-D-glucoside | 4.12 | [M+H]^+^ | C_19_H_24_O_9_ | 397.1488 | -1.3 | Flavonoids | RP | 235.0962，191.0700 |
| 34 | Liquiritin *or* Neoisoliquiritigenin | 5.29 | [M+H]^+^ | C_21_H_22_O_9_ | 419.1336 | -0.1 | Flavonoids | PS | 257.0803，419.1340 |
| 35 | 2-Methyl-5-(2'-oxo-4'-hydroxybutyl)-7-hydroxychromone-7-*O*-*β*-D-glucoside | 3.94 | [M+H]^+^ | C_21_H_26_O_10_ | 439.1588 | -2.4 | Flavonoids | RP | 277.1076，439.1610 |
| 36 | Glycitin | 5 | [M+H]^+^ | C_22_H_22_O_10_ | 447.1283 | -0.6 | Flavonoids | AM | 285.0758，270.0524，447.1285 |
| 37 | Tectoridin^L^ | 5.68 | [M+H]^+^ | C_22_H_22_O_11_ | 463.123 | -1.1 | Flavonoids | PS | 301.0702 |
| 38 | Isomucronulatol-7-*O*-glucoside | 6.57 | [M+H]^+^ | C_23_H_28_O_10_ | 465.1749 | -1.3 | Flavonoids | AM | 167.0699，123.0436 |
| 39 | Complanatuside | 3.44 | [M+H]^+^ | C_28_H_32_O_16_ | 625.1762 | -0.2 | Flavonoids | AM | 301.0701，463.1223，625.1806 |
| 40 | Ononin | 6.16 | [M+COOH]^-^ | C_22_H_22_O_9_•HCOOH | 475.1230 | -3.3 | Flavonoids | AM | 267.0703，431.1003，268.0399，475.0923，252.0439，269.0467 |
| 41 | 2-Hydroxy-6-methylanthraquinone *or* 2-Hydroxy-7-methylanthraquinone | 9.68 | [M-H]^-^ | C_15_H_10_O_3_ | 237.0551 | -2.6 | Anthraquinones | HD | 237.0554，236.0479，209.0613 |
| 42 | 2-Methyl-3-hydroxyanthraquinone | 9.32 | [M-H]^-^ | C_15_H_10_O_3_ | 237.0555 | -0.9 | Anthraquinones | HD | 237.0578，209.0614 |
| 43 | Chrysophanol | 9.65 | [M-H]^-^ | C_15_H_10_O_4_ | 253.0505 | -0.5 | Anthraquinones | RP | 253.0519，225.0559 |
| 44 | 2-Hydroxy-3-hydroxymethylanthraquinone | 6.81 | [M-H]^-^ | C_15_H_10_O_4_ | 253.0508 | 0.7 | Anthraquinones | HD | 253.0535，225.0571 |
| 45 | 8-Methyl chrysophanol | 8.16 | [M-H]^-^ | C_16_H_12_O_4_ | 267.0657 | -2.2 | Anthraquinones | RP | 252.0439，223.0415，267.0671，195.0458，251.0357 |
| 46 | 2-Hydroxy-1-methoxyanthraquinone | 8.18 | [M-H]^-^ | C_15_H_10_O_4_ | 253.0505 | -0.5 | Anthraquinones | HD | 210.0333，253.0519，238.0285 |
| 47 | 2-Hydroxy-3-methoxy-6-methylanthraquinone *or* 2-Hydroxy-3-methoxy-7-methylanthraquinone | 6.69 | [M-H]^-^ | C_16_H_12_O_4_ | 267.0658 | -1.8 | Anthraquinones | HD | 252.0435，267.0668，223.0410，195.0457，251.0358 |
| 48 | 2,6-Dihydroxy-1-methoxyanthraquinone | 7.21 | [M-H]^-^ | C_15_H_10_O_5_ | 269.0448 | -2.8 | Anthraquinones | HD | 254.0234，226.0283，198.0328，269.0460 |
| 49 | Aloe-emodin^S^ | 8.47 | [M-H]^-^ | C_15_H_10_O_5_ | 269.0452 | -1.3 | Anthraquinones | RP | 269.0454，240.0432，239.0352，183.0457 |
| 50 | Emodin^S^ | 10.46 | [M-H]^-^ | C_15_H_10_O_5_ | 269.0459 | 1.3 | Anthraquinones | RP | 269.0467，225.0566，241.0514，197.0619 |
| 51 | 2,3-Dimethoxy-6-methylanthraquinone | 7.21 | [M-H]^-^ | C_17_H_14_O_4_ | 281.0813 | -2.2 | Anthraquinones | HD | 281.0836，253.0871，281.1191，252.0788 |
| 52 | Rhein^S^ | 9.77 | [M-H]^-^ | C_15_H_8_O_6_ | 283.0251 | 1.0 | Anthraquinones | RP | 183.0544，211.0441，239.0462，283.0276 |
| 53 | 2,6-Dihydroxy-1-methoxy-3-methylanthraquinone | 6.84 | [M-H]^-^ | C_16_H_12_O_5_ | 283.0604 | -2.8 | Anthraquinones | HD | 268.0387，211.0405，283.0631，239.0364，135.0089 |
| 54 | 2,6-Dihydroxy-3-methyl-4-methoxyanthraquinone *or* 1,7-Dihydroxy-6-methoxy-2-methylanthraquinone | 7.14 | [M-H]^-^ | C_16_H_12_O_5_ | 283.0607 | -1.8 | Anthraquinones | HD | 240.0435，283.0617 |
| 55 | Physcion | 7.31 | [M-H]^-^ | C_16_H_12_O_5_ | 283.0611 | -0.4 | Anthraquinones | RP | 240.0444，283.0623 |
| 56 | 6-Methyl-rhein | 9.65 | [M-H]^-^ | C_16_H1_0_O_6_ | 297.0406 | 0.5 | Anthraquinones | RP | 253.0630，225.0591，224.0487，297.0416 |
| 57 | laccaic acid D | 8.41 | [M-H]^-^ | C_16_H_10_O_7_ | 313.0354 | 0.1 | Anthraquinones | RP | 269.0474，225.0565，241.0520 |
| 58 | Chrysophanol-8-*O*-*β*-D-glucopyranoside^L^ *or* Chrysophanol-1-*O*-*β*-D-glucopyranoside | 6.82 | [M-H]^-^ | C_21_H_20_O_9_ | 415.1036 | 0.3 | Anthraquinones | RP | 253.0568，225.0560，277.0519 |
| 59 | Emodin-6-*O*-*β*-D-glucoside | 7.31 | [M-H]^-^ | C_21_H_20_O_10_ | 431.0977 | -1.6 | Anthraquinones | RP | 269.0458，282.0539，241.0509 |
| 60 | Emodin-8-*O*-*β*-D-glucoside^L^ | 6.3 | [M-H]^-^ | C_21_H_20_O1_0_ | 431.0977 | -1.6 | Anthraquinones | RP | 269.0464，311.0576，431.1005 |
| 61 | Emodin-1-*O*-*β*-D-glucoside | 6.05 | [M-H]^-^ | C_21_H_20_O_10_ | 431.0981 | -0.6 | Anthraquinones | RP | 431.0997，269.0471，240.0441 |
| 62 | Aloe-emodin-8-*O*-*β*-D-glucopyranoside *or* Aloe-emodin-1-*O*-*β*-D-glucopyranoside | 5 | [M-H]^-^ | C_21_H_20_O_10_ | 431.0983 | -0.2 | Anthraquinones | RP | 269.0487，293.0476 |
| 63 | Aloe-emodin-ω-*O*-*β*-D-glucopyranoside | 6.75 | [M-H]^-^ | C_21_H_20_O_10_ | 431.0986 | 0.5 | Anthraquinones | RP | 269.0464，431.0991 |
| 64 | Rhein-8-*O*-*β*-D-glucopyranoside | 5.79 | [M-H]^-^ | C_21_H_18_O_11_ | 445.0784 | 1.7 | Anthraquinones | RP | 239.0359，283.0258 |
| 65 | Emodin-8-*O*-(6-*O*-acetyl)-glucoside | 6.71 | [M-H]^-^ | C_23_H_22_O_11_ | 473.1079 | -2.2 | Anthraquinones | RP | 311.0573，473.1103 |
| 66 | Aloe-emodin-8-*O*-(6-*O*-acetyl)-glucoside | 5.84 | [M-H]^-^ | C_23_H_22_O_11_ | 473.1083 | -1.4 | Anthraquinones | RP | 311.0578，473.1102 |
| 67 | Physcion-8-*O*-*β*-D-(6-acetyl)-glucoside | 8.44 | [M-H]^-^ | C_24_H_24_O_11_ | 487.1240 | -1.2 | Anthraquinones | RP | 283.0611，240.0432 |
| 68 | Chrysophanol-8-*O*-(6-*O*-malonyl)-glucoside | 6.38 | [M-H]^-^ | C_24_H_22_O_12_ | 501.1036 | -0.5 | Anthraquinones | RP | 253.0521，501.1057，457.1157 |
| 69 | Emodin-8-*O*-(6-*O*-malonyl)-glucoside | 7.33 | [M-H]^-^ | C_24_H_22_O_13_ | 517.0984 | -0.7 | Anthraquinones | RP | 269.0462，473.1101 |
| 70 | Chrysophanol diglucoside | 5.88 | [M-H]^-^ | C_27_H_30_O_14_ | 577.1561 | -0.3 | Anthraquinones | RP | 253.0512，577.1588 |
| 71 | Rhein diglucoside | 5.1 | [M-H]^-^ | C_27_H_28_O_16_ | 607.1302 | -0.4 | Anthraquinones | RP | 607.1358，283.0254 |
| 72 | 2-Hydroxy-1,3-dimethoxyanthraquinone | 6.03 | [M+H]^+^ | C_16_H_12_O_5_ | 285.0753 | -1.6 | Anthraquinones | HD | 285.0757，270.0520，137.0228 |
| 73 | 1,3,8-Trihydroxy-6-Hydroxymethylanthraquinone | 4.94 | [M+H]^+^ | C_15_H_10_O_6_ | 287.0553 | 1.0 | Anthraquinones | RP | 287.0550，241.0492 |
| 74 | Physcion-8-*O*-*β*-D-gentianoside | 6.03 | [M+H]^+^ | C_28_H_32_O_14_ | 593.1851 | -2.3 | Anthraquinones | RP | 269.0802，593.1853 |
| 75 | 4-Aminobutyric acid | 0.95 | [M-H]^-^ | C_4_H_9_NO_2_ | 102.0562 | 1.5 | Amino acids | AM | 102.0568 |
| 76 | L-Aspartic acid^L^ *or* Asparagine | 0.97 | [M-H]^-^ | C_4_H_7_NO_4_ | 132.0303 | 0.5 | Amino acids | AM/HN | 88.0439，115.0042，132.0314 |
| 77 | L-Glutamic acid | 0.95 | [M-H]^-^ | C_5_H_9_NO_4_ | 146.0463 | 2.9 | Amino acids | AM | 102.0565，128.0356，146.0459 |
| 78 | Histidine | 0.9 | [M-H]^-^ | C_6_H_9_N_3_O_2_ | 154.0617 | -3.2 | Amino acids | HN | 93.0466，81.0462，137.0362，154.0636 |
| 79 | Arginine | 0.88 | [M-H]^-^ | C_6_H_14_N_4_O_2_ | 173.1043 | -0.6 | Amino acids | HN | 131.0844 |
| 80 | Tryptophan | 3.19 | [M-H]^-^ | C_11_H_12_N_2_O_2_ | 203.0820 | -3.0 | Amino acids | PS/HN | 116.0510，142.0662，97.0301，203.0835 |
| 81 | Proline | 0.98 | [M+H]^+^ | C_5_H_9_NO_2_ | 116.0704 | -1.8 | Amino acids | AM/HN | 116.0705 |
| 82 | DL-Threonine | 2.13 | [M+H]^+^ | C_4_H_9_NO_3_ | 120.0651 | -3.5 | Amino acids | AM | 103.0383，102.0123，85.0280 |
| 83 | L-Isoleucine *or* Leucine | 1.8 | [M+H]^+^ | C_6_H1_3_NO_2_ | 132.1017 | -1.6 | Amino acids | AM/HN | 86.0962 |
| 84 | L-Methionine | 1.38 | [M+H]^+^ | C_5_H_11_NO_2_S | 150.0574 | -6.2 | Amino acids | AM | 104.0525，133.0311，87.0258，102.0547 |
| 85 | Phenylalanine | 2.75 | [M+H]+ | C_9_H_11_NO_2_ | 166.0859 | -1.2 | Amino acids | AM/HN | 120.0807，103.0540，91.0539，93.0695 |
| 86 | Danshenxinkun B | 9.52 | [M-H]^-^ | C_18_H_18_O_3_ | 281.1181 | -0.8 | Terpenoids | SM | 281.1189，265.0876，237.0919 |
| 87 | Danshenxinkun A *or* Tanshinone VI *or* Przewaquinone C | 9.63 | [M-H]^-^ | C_18_H_16_O_4_ | 295.0973 | -0.9 | Terpenoids | SM | 295.0976，96.9594，237.0931，265.0862，267.1016，295.1584，277.0839 |
| 88 | Tanshindiol C *or* Tanshindiol B | 6.56 | [M-H]^-^ | C_18_H_16_O_5_ | 311.0916 | -2.9 | Terpenoids | SM | 281.0475，253.0501，296.0699，311.0849，281.0614 |
| 89 | Carnosol | 9.19 | [M-H]^-^ | C_20_H_26_O_4_ | 329.1754 | -1.3 | Terpenoids | SM | 285.1853，329.2346，267.1401，285.0386，329.1974 |
| 90 | Methyl tanshinonate | 6.97 | [M-H]^-^ | C_20_H_18_O_5_ | 337.1074 | -2.2 | Terpenoids | SM | 307.0619，279.0677，322.0851，291.0667，235.0747，306.0883， |
| 91 | Geniposidic acid | 3.11 | [M-H]^-^ | C_16_H_22_O_10_ | 373.1132 | -2.2 | Terpenoids | HD | 123.0455，149.0611 |
| 92 | Deacetyl asperulosidic acid *or* Scandoside | 2.81 | [M-H]^-^ | C_16_H_22_O_11_ | 389.1094 | 1.2 | Terpenoids | HD | 165.0567，183.0677，389.1118，147.0459，89.0250，209.0466，227.0566 |
| 93 | Asperulosidic acid | 3.61 | [M-H]^-^ | C_18_H_24_O_12_ | 431.1199 | 0.9 | Terpenoids | HD | 431.1205，89.0251，165.0566，147.0456 |
| 94 | Oleanolic acid^S^ *or* Ursolic acid^S^ | 15.39 | [M-H]^-^ | C_30_H_48_O_3_ | 455.3526 | -1.0 | Terpenoids | HD/SM | 455.3535 |
| 95 | Tormentic acid *or* Barbinervic acid | 10.56 | [M-H]^-^ | C_30_H_48_O_5_ | 487.3418 | -2.3 | Terpenoids | SM | 443.2722，487.3416，425.3369，487.2960 |
| 96 | (*E*)-6-*O*-Coumaroyl scandoside methyl ester-10-*O*-methyl ether | 4.84 | [M-H]^-^ | C_27_H_32_O_13_ | 563.1766 | -0.7 | Terpenoids | HD | 281.0822，443.1349，563.1781 |
| 97 | (*E*)-6-*O*-*p*-Feruloyl scandoside methyl ester *or* (*Z*)-6-*O*-*p*-Feruloyl scandoside methyl ester | 4.79 | [M-H]^-^ | C_27_H_32_O_14_ | 579.1701 | -3.2 | Terpenoids | HD | 579.1712，96.9601，579.1472，133.0142，533.1732，417.1309 |
| 98 | 10-*O*-Benzoyl-1-*O*-(6-*O-α*-L-arabinopyranosyl)-*β*-D-glucopyranosyl geniposidic acid | 5.9 | [M-H]^-^ | C_28_H_34_O_15_ | 609.1819 | -1.0 | Terpenoids | HD | 609.1839，121.0304，149.0586，89.0247，193.0512，487.1465 |
| 99 | Soyasaponin I | 8.65 | [M-H]^-^ | C_48_H_78_O_18_ | 941.5114 | -0.1 | Terpenoids | AM | 941.5152 |
| 100 | Polygonoide C | 9.62 | [M-H]^-^ | C_48_H_78_O_19_ | 957.5048 | -1.7 | Terpenoids | PS | 89.0249，957.5078 |
| 101 | 1,2-Dihydrotanshinquinone *or* Methylene tanshinquinone | 10.18 | [M+H]^+^ | C_18_H_14_O_3_ | 279.1010 | -2.0 | Terpenoids | SM | 279.1010，261.0905，233.0955，205.1003 |
| 102 | Nortanshinone | 8.98 | [M+H]^+^ | C_17_H_12_O_4_ | 281.0804 | -1.6 | Terpenoids | SM | 235.0749，207.0802，281.0805，253.0856，263.0698 |
| 103 | 1,2,15,16-Tetrahydrotanshiquinone *or* Methylenedihydrotanshinquinone | 10.51 | [M+H]^+^ | C_18_H_16_O_3_ | 281.1162 | -3.6 | Terpenoids | SM | 235.1115，192.0930，263.1059 |
| 104 | Tanshinone IIA^S^ | 12.56 | [M+H]^+^ | C_19_H_18_O_3_ | 295.1322 | -2.3 | Terpenoids | SM | 295.1322，277.1211，249.1262 |
| 105 | Cryptotanshinone^S^ | 11.28 | [M+H]^+^ | C_19_H_20_O_3_ | 297.1481 | -1.4 | Terpenoids | SM | 297.1482，251.1423 |
| 106 | Sugiol | 11.75 | [M+H]^+^ | C_20_H_28_O_2_ | 301.2150 | -4.0 | Terpenoids | SM | 301.2143，301.1410，259.1682，301.1758，163.0747 |
| 107 | Miltionone II | 7.9 | [M+H]^+^ | C_19_H_20_O_4_ | 313.1429 | -1.7 | Terpenoids | SM | 313.1436，295.1321，267.1366，255.1012 |
| 108 | 17-Hydroxytanshindiol B | 5.9 | [M+H]^+^ | C_18_H_18_O_5_ | 315.1221 | -1.9 | Terpenoids | SM | 269.1163，297.1112，251.1061，223.1105，315.1222 |
| 109 | Tanshinone V | 10.66 | [M+H]^+^ | C_19_H_22_O_4_ | 315.1582 | -2.8 | Terpenoids | SM | 297.1477，315.1584，279.1371 |
| 110 | Methyl dihydronortanshinonate | 9.48 | [M+H]^+^ | C_20_H_20_O5 | 341.1377 | -1.9 | Terpenoids | SM | 235.1110，341.1366，263.1052，281.1162 |
| 111 | Asperuloside | 3.57 | [M+H]+ | C_18_H_22_O_11_ | 415.1229 | -1.4 | Terpenoids | HD | 147.0440，175.0384，119.0489，91.0541，193.0486，235.0589，165.0541 |
| 112 | (25*S*)-Kingianoside A | 6.34 | [M+H]^+^ | C_39_H_60_O_14_ | 753.4019 | -4.9 | Terpenoids | PS | 591.3532，753.4036，429.3022，753.2804，753.3727，183.1366 |
| 113 | Astragaloside II *or* Isoastragaloside II | 7.66 | [M+H]^+^ | C_43_H_70_O_15_ | 827.4779 | -1.0 | Terpenoids | AM | 175.0600，143.1060，157.0491，437.3402，455.3521，473.3608，419.3284 |
| 114 | Isoastragaloside I | 8.52 | [M+H]^+^ | C_45_H_72_O_16_ | 869.4854 | -4.5 | Terpenoids | AM | 157.0493，217.0704，143.1072 |
| 115 | Astragaloside IV^S^ | 7.81 | [M+COOH]^-^ | C_41_H_68_O_14_•HCOOH | 829.4592 | 0.1 | Terpenoids | AM | 829.4597，783.4540 |
| 116 | Astragaloside III | 7.91 | [M+COOH]^-^ | C_41_H_68_O_14_•HCOOH | 829.4597 | 0.7 | Terpenoids | AM | 783.4557，829.4602，784.4601 |
| 117 | 5-Oxo-pyrrolidine-2-carboxylic acid methyl ester | 2.87 | [M-H]^-^ | C_6_H_9_NO_3_ | 142.0507 | -1.9 | Alkaloids | PS | 100.0413，142.0515 |
| 118 | 5-Hydroxy-pyridine-2-carboxylate | 4.14 | [M-H]^-^ | C_7_H_7_NO_3_ | 152.0351 | -1.4 | Alkaloids | PS | 93.0224，152.0384 |
| 119 | 1-(5-Hydroxym-ethyl-tetrahydro-furan-2-yl)-9*H*-*β*-carboline-3-carboxylic acid | 6.85 | [M-H]^-^ | C_17_H_12_N_2_O_4_ | 307.0719 | -1.7 | Alkaloids | PS | 233.0732，205.0777，263.0838，96.9604 |
| 120 | Betaine | 0.93 | [M+H]^+^ | C_5_H_11_NO_2_ | 118.0861 | -1.4 | Alkaloids | AM | 118.0863 |
| 121 | 5-(Methoxymethyl)-l*H*-pyrrole-2-carbaldehyde | 3.25 | [M+H]^+^ | C_6_H_7_NO_2_ | 126.0547 | -2.1 | Alkaloids | SM | 80.0492，108.0447，126.0544 |
| 122 | Polygonatine A | 3.58 | [M+H]^+^ | C_9_H_11_NO_2_ | 166.0861 | -1.0 | Alkaloids | PS | 166.0862，136.0755，80.0493，118.0648，148.0753 |
| 123 | Salviadione | 8.69 | [M+H]^+^ | C_19_H_19_NO_2_ | 294.1482 | -2.2 | Alkaloids | SM | 294.1481，279.1249 |
| 124 | (+)-Catechin-4'-*O*-*β*-D-glucopyranoside *or* (+)-Catechin-7-*O*-*β*-D-glucopyranoside *or* (+)-Catechin-5-*O*-*β*-D-glucopyranoside *or* (+)-Ctechin-3'-*O*-*β*-D-glucopyranoside | 3.01 | [M-H]^-^ | C_21_H_24_O_11_ | 451.1242 | -0.9 | Flavonoids | RP | 331.0825，89.0240，109.0291，109.0391，361.0929，451.1248 |
| 125 | (+)-Epicatechin *or* D-Catechin | 3.46 | [M+H]^+^ | C_15_H_14_O_6_ | 291.0857 | -2.1 | Flavonoids | RP | 139.0390，123.0440，147.0439，165.0544 |
| 126 | Cinnamic acid | 7.18 | [M-H]^-^ | C_9_H_8_O_2_ | 147.0448 | -2.4 | Phenylpropanoids | RP | 103.0565，147.0452 |
| 127 | *p*-Coumaric acid | 5.3 | [M-H]^-^ | C_9_H_8_O_3_ | 163.0402 | 0.8 | Phenylpropanoids | HD | 119.0605，93.0392 |
| 128 | *Trans*-4-methoxycinnamic acid | 4.81 | [M-H]^-^ | C_10_H_10_O_3_ | 177.0554 | -1.8 | Phenylpropanoids | HD | 133.0290，177.0216，117.0351，105.0334，132.9722 |
| 129 | Caffeic acid^S^ | 4.52 | [M-H]^-^ | C_9_H_8_O_4_ | 179.0348 | -1.0 | Phenylpropanoids | HD/SM | 135.0475，134.0380 |
| 130 | Tanshinol | 6.46 | [M-H]^-^ | C_9_H_10_O_5_ | 197.0450 | -2.8 | Phenylpropanoids | SM | 135.0458，123.0463，134.0380，179.0361 |
| 131 | Salvianolic acid F | 6.02 | [M-H]^-^ | C_17_H_14_O_6_ | 313.0716 | -0.5 | Phenylpropanoids | SM | 161.0252，133.0303，109.0300 |
| 132 | Salvianolic acid G | 5.82 | [M-H]^-^ | C_18_H_12_O_7_ | 339.0510 | -0.1 | Phenylpropanoids | SM | 109.0300，295.0625，185.0250，339.0531，277.0513，280.0379，252.0425 |
| 133 | Przew alskinic acid A | 5.35 | [M-H]^-^ | C_18_H_14_O_8_ | 357.0615 | -0.3 | Phenylpropanoids | SM | 109.0301，269.0823，159.0457，203.0350，313.0724 |
| 134 | Rosmarinic acid^S^ | 5.98 | [M-H]^-^ | C_18_H_16_O_8_ | 359.0782 | 2.7 | Phenylpropanoids | SM | 161.0336，135.0476，133.0324，197.0493，179.0363 |
| 135 | Salvianolic acid M | 5.67 | [M-H]^-^ | C_19_H_16_O_8_ | 371.0770 | -0.6 | Phenylpropanoids | SM | 135.0457，145.0301，197.0466，173.0248，179.0353 |
| 136 | Salvianolic acid D | 5.67 | [M-H]^-^ | C_20_H_18_O_10_ | 417.0834 | 1.6 | Phenylpropanoids | SM | 175.0407，197.0460，179.0355，135.0456，417.0830，157.0298 |
| 137 | Salvianolic acid C | 6.6 | [M-H]^-^ | C_26_H_20_O_10_ | 491.0982 | -0.3 | Phenylpropanoids | SM | 293.0471，311.0578，135.0458，491.0992，265.0513，179.0354 |
| 138 | Salvianolic acid N | 6.31 | [M-H]^-^ | C_26_H_22_O_10_ | 493.1140 | 0.0 | Phenylpropanoids | SM | 295.0616，185.0252 |
| 139 | Salvianolicacid A | 6.6 | [M-H]^-^ | C_26_H_22_O_10_ | 493.1143 | 0.6 | Phenylpropanoids | SM | 295.0647，185.0261 |
| 140 | Salviaflaside | 5.34 | [M-H]^-^ | C_24_H_26_O_13_ | 521.1291 | -1.8 | Phenylpropanoids | SM | 161.0249，323.0777，359.0799，179.0355，197.0458 |
| 141 | Salvianolic acid H | 6.31 | [M-H]^-^ | C_27_H_22_O_12_ | 537.1032 | -1.2 | Phenylpropanoids | SM | 295.0626，185.0257，109.0306 |
| 142 | Salvianolic acid T | 5.36/5.45 | [M-H]^-^ | C_27_H_22_O_12_ | 537.1035 | -0.7 | Phenylpropanoids | SM | 295.0624，185.0251，109.0299，493.1145 |
| 143 | Salvianolic acid J | 6.2 | [M-H]^-^ | C_27_H_22_O_12_ | 537.1036 | -0.5 | Phenylpropanoids | SM | 185.0267，295.0668，109.0306，493.1171 |
| 144 | Lithospermic acid | 5.83 | [M-H]^-^ | C_27_H_22_O_12_ | 537.1045 | 1.2 | Phenylpropanoids | SM | 339.0569，295.0633 |
| 145 | Monomethyl lithospermate | 6.67 | [M-H]^-^ | C_28_H_24_O_12_ | 551.1186 | -1.6 | Phenylpropanoids | SM | 197.0465，309.0773，294.0540，507.1299，179.0357 |
| 146 | Salvianolic acid K | 5.13 | [M-H]^-^ | C_27_H_24_O_13_ | 555.1140 | -0.7 | Phenylpropanoids | SM | 313.0728，295.0622，185.0257，357.0626 |
| 147 | Salvianolic acid E | 5.94 | [M-H]^-^ | C_36_H_30_O_16_ | 717.1461 | 0.0 | Phenylpropanoids | SM | 295.0625，519.0958，717.1479，321.0416，493.1160，537.1059，339.0523 |
| 148 | Salvianolic acid L | 6.15 | [M-H]^-^ | C_36_H_30_O_16_ | 717.1469 | 1.1 | Phenylpropanoids | SM | 519.0952，321.0418，339.0519，717.1470，295.0622 |
| 149 | Salvianolic acid B^S^ | 6.47 | [M-H]^-^ | C_36_H_30_O_16_ | 717.1469 | 1.1 | Phenylpropanoids | SM | 519.0956，321.0416，339.0520，717.1478，295.0621 |
| 150 | Salvianolic acid Y | 6.51 | [M-H]^-^ | C_36_H_30_O_16_ | 717.1472 | 1.5 | Phenylpropanoids | SM | 519.0952，321.0417，339.0520，717.1483，295.0620 |
| 151 | Sagerinic acid | 6 | [M-H]^-^ | C_36_H_32_O_16_ | 719.1617 | -0.1 | Phenylpropanoids | SM | 359.0824，161.0270，197.0469，179.0363 |
| 152 | Esculetin | 4.32 | [M+H]^+^ | C_9_H_6_O_4_ | 179.0329 | -5.5 | Phenylpropanoids | HD | 123.0437，179.0333，133.0276，151.0384 |
| 153 | Scopoletin | 5.33 | [M+H]^+^ | C_10_H_8_O_4_ | 193.0494 | -0.7 | Phenylpropanoids | HD | 193.0498，133.0283，178.0260 |
| 154 | Ferulic acid^S^ | 5.54 | [M+H]^+^ | C_10_H_10_O_4_ | 195.0646 | -3.0 | Phenylpropanoids | HD/SM | 89.0383，177.0552，117.0334，145.0287，149.0591，134.0361 |
| 155 | Isofraxidin | 5.41 | [M+H]^+^ | C_11_H_10_O_5_ | 223.0598 | -1.3 | Phenylpropanoids | AM | 223.0598，162.0309，190.0260 |
| 156 | Hedyotiscone B | 6.26 | [M+H]^+^ | C_14_H_12_O_4_ | 245.0804 | -1.8 | Phenylpropanoids | HD | 245.0806，158.0723，201.0905，199.0748 |
| 157 | Succinic acid | 2.53 | [M-H]^-^ | C_4_H_6_O_4_ | 117.0192 | -1.1 | Others | RP | 99.0094，99.9262，117.0199 |
| 158 | 4-Methylcatechol | 3.2 | [M-H]^-^ | C_7_H_8_O_2_ | 123.0450 | -1.2 | Others | AM | 123.0461，122.0372，108.0216 |
| 159 | 5-Hydroxymethylfurfural | 2.85 | [M-H]^-^ | C_6_H_6_O_3_ | 125.0244 | -0.2 | Others | HD/PS | 125.0251，124.0174 |
| 160 | Protocatechualdehyde^S^ | 3.99 | [M-H]^-^ | C_7_H_6_O_3_ | 137.0244 | -0.1 | Others | SM | 137.0270，136.0173，119.0146 |
| 161 | *p*-Hydroxybenzoic acid | 7.25 | [M-H]^-^ | C_7_H_6_O_3_ | 137.0245 | 0.6 | Others | HD/RP/SM | 93.0429，137.0262 |
| 162 | 4-Vinyl-2-methoxyphenol | 7.01 | [M-H]^-^ | C_9_H_10_O_2_ | 149.0605 | -2.0 | Others | HD | 149.0610，105.0704，105.0367，92.0263 |
| 163 | Gallic acid | 2.87 | [M-H]^-^ | C_7_H_6_O_5_ | 169.0144 | 0.9 | Others | RP/SM | 125.0328，124.0176，81.0355，97.0304，169.0169 |
| 164 | Citric acid | 2.46 | [M-H]^-^ | C_6_H_8_O_7_ | 191.0200 | 1.4 | Others | RP | 87.0106，111.0137，85.0310，191.0216 |
| 165 | Uridine^S^ | 2.7 | [M-H]^-^ | C_9_H_12_N_2_O_6_ | 243.0617 | -2.3 | Others | HN | 110.0254，82.0303 |
| 166 | Danshenspiroketallactone | 7.29 | [M-H]^-^ | C_17_H_16_O_3_ | 267.1022 | -1.8 | Others | SM | 267.1030，223.1346，237.0915，267.0903，235.0773，249.0925 |
| 167 | Salvianonol | 8.76 | [M-H]^-^ | C_18_H_20_O_4_ | 299.1284 | -1.6 | Others | SM | 255.1386，237.1303，239.1069 |
| 168 | Methyl rosmarinate | 5.67 | [M-H]^-^ | C_19_H_18_O_8_ | 373.0926 | -0.8 | Others | SM | 175.0400，135.0449，197.0457，179.0350 |
| 169 | Torachrysone-8-*O*-*β*-D-glucopyranoside | 6.65 | [M-H]^-^ | C_20_H_24_O_9_ | 407.1349 | 0.3 | Others | HD | 245.0875，230.0615，215.0361 |
| 170 | Rhapontigenin-3'-*O*-*β*-D-glucopyranoside *or* Rhapontin *or* *cis*-3,5,3'-Trihydroxyl-4'-methoxystilbene-3-*O*-*β*-D-glucopyranoside *or* Isorhapontin | 4.97 | [M-H]^-^ | C_21_H_24_O_9_ | 419.1340 | -1.8 | Others | RP | 257.0822，121.0285，149.0232，419.1352 |
| 171 | Astrernestin | 7.72 | [M-H]^-^ | C_25_H_20_O_8_ | 447.1077 | -1.9 | Others | AM | 295.0615，185.0240，109.0301 |
| 172 | Lindleyin *or* Isolindleyin | 5.12 | [M-H]^-^ | C_23_H_26_O_11_ | 477.1403 | 0.1 | Others | RP | 477.1419，169.0153，313.0573，125.0254，163.0768 |
| 173 | 2-Piperidone | 2.91 | [M+H]^+^ | C_5_H_9_NO | 100.0752 | -4.9 | Others | HN | 100.0755，82.0649 |
| 174 | Ncotinic acid | 1.75 | [M+H]^+^ | C_6_H_5_NO_2_ | 124.0391 | -1.6 | Others | HN | 124.0388，80.0490 |
| 175 | Hypoxanthine *or* 2-Hydroxypurine | 1.97 | [M+H]^+^ | C_5_H_4_N_4_O | 137.0456 | -1.4 | Others | HN | 137.0454，110.0342，119.0346，82.0394 |
| 176 | Indole-3-carbaldehyde | 3.17 | [M+H]^+^ | C_9_H_7_NO | 146.0596 | -3.0 | Others | HN | 91.0538，118.0645，117.0568，146.0600 |
| 177 | 2,6-Dihydroxypurine | 2.38 | [M+H]^+^ | C_5_H_4_N_4_O_2_ | 153.0401 | -3.9 | Others | HN | 110.0345，136.0135，153.0401，81.0080，82.0394 |
| 178 | 3,4-Dihydroxybenzoic acid | 3.42 | [M+H]^+^ | C_7_H_6_O_4_ | 155.0335 | -2.5 | Others | HD/SM | 93.0334，81.0334，137.0242，111.0441 |
| 179 | (*E*)-4-[5-(Hydroxymethyl)-2-furanyl]-3-butene-2-one | 6.42 | [M+H]^+^ | C_9_H_10_O_3_ | 167.0699 | -2.2 | Others | SM | 167.0696，105.0327，106.0409，152.0471，134.0349，123.0440 |
| 180 | Resveratrol | 4.67 | [M+H]^+^ | C_14_H_12_O_3_ | 229.0858 | -0.5 | Others | RP | 107.0486，91.0538，135.0433，229.0854，165.0685 |
| 181 | Adenosine | 2.55 | [M+H]^+^ | C_10_H_13_N_5_O_4_ | 268.1033 | -2.7 | Others | PS/HN | 136.0616，119.0349，268.1036 |
| 182 | Dibutyl phthalate | 12.14 | [M+H]^+^ | C_16_H_22_O_4_ | 279.1584 | -2.5 | Others | HD/RP | 149.0228，121.0280 |
| 183 | Guanosine | 2.62 | [M+H]^+^ | C_10_H_13_N_5_O_5_ | 284.0999 | 3.3 | Others | HN | 152.0565，135.0301 |
| 184 | 4,4'-Dihydroy-*α*-truxillic acid | 6.79 | [M+H]^+^ | C_18_H_16_O_6_ | 329.1017 | -0.8 | Others | HD | 283.0964，269.0807，240.0782，329.1026 |
| 185 | Stigmasta-5,22-diene-3*β*-7*α*-diol *or* Stigmasta-5,22-diene-3*β*-7*β*-diol | 16.27 | [M+H]^+^ | C_29_H_48_O_2_ | 429.3714 | -3.1 | Others | HD | 165.0906，429.3718 |
| 186 | Aurantiamide acetate | 9.58 | [M+H]^+^ | C_27_H_28_N_2_O4 | 445.2112 | -2.2 | Others | HD | 105.0328，194.1169，224.1058，117.0693，134.0958 |
| 187 | 1-*O*-hexadecyl-*sn*-glycero-4-phosphocholine | 11.84 | [M+H]^+^ | C_24_H_52_NO_6_P | 482.3593 | -2.5 | Others | HN | 104.1065，482.3587，184.0728 |
| 188 | 1-*O*-hexadecanoyl-*sn*-glycero-4-phosphocholine | 11.48 | [M+H]^+^ | C_24_H_50_NO_7_P | 496.3385 | -2.6 | Others | HN | 184.0722，496.3376，104.1059，86.0956 |
| 189 | 1-*O*-octadecanoyl-*sn*-glycero-4-phosphocholine | 12.75 | [M+H]^+^ | C_26_H_54_NO_7_P | 524.3701 | -1.8 | Others | HN | 184.0730，524.3698，104.1068 |
| 190 | (25*S*)-Pratioside D1 *or* Pratioside D1 | 5.31 | [M+H]^+^ | C_45_H_70_O_19_ | 915.4578 | -0.7 | Others | PS | 915.4571，429.3000，411.2889，591.3512，753.4062 |

^S^ means compared with standard substances.

**Table S2** Prototype compounds of ZJTSD absorbed in rat serum.

| No | Component Name | Rt (min) | Adduct | Formula | Found at Mass | Error | Category | Source | Fragments |
| --- | --- | --- | --- | --- | --- | --- | --- | --- | --- |
| P1 | 2,5-Dimethyl-7-hydroxychromone | 6.37 | [M-H]- | C11H10O3 | 189.055 | -3.8 | Flavonoids | RP | 189.0558，146.0374，147.0450，159.0451，105.0347，133.0661 |
| P2 | 7-Hydroxy-2-(2-hydroxypropyl)-5-methylchromen-4-one | 5.64 | [M-H]- | C13H14O4 | 233.0811 | -3.6 | Flavonoids | RP | 189.0564，233.0829，187.0404，188.0479，159.0451，105.0348，161.0610，149.0248，123.0449 |
| P3 | 3-Methoxy-5,7-dihydroxyflavone | 7.02 | [M-H]- | C16H12O5 | 283.0597 | -5.3 | Flavonoids | HD | 268.0375，239.0352，267.0298，283.0609，223.0392，251.0345，210.0321 |
| P4 | 2-Methyl-5-carboxymethyl-7-hydroxychromone | 5.1 | [M+H]+ | C12H10O5 | 235.0595 | -2.6 | Flavonoids | RP | 189.0545，217.0501，161.0597 |
| P5 | 2-Methyl-5-acetonyl-7-hydroxychromone | 5.58 | [M+H]+ | C13H12O4 | 233.0801 | -3.2 | Flavonoids | RP | 233.0799，191.0702，215.0698，190.0617 |
| P6 | 2,5-Dimethyl-7-methoxychromone | 12.11 | [M+H]+ | C12H12O3 | 205.0853 | -3 | Flavonoids | RP | 149.0226，93.0329，121.0278 |
| P7 | 2-(2-Hydroxypropyl)-5-methyl-7-hydroxychromone-7-O-β-D-glucoside | 4.13 | [M+H]+ | C19H24O9 | 397.1487 | -1.5 | Flavonoids | RP | 235.0964，191.0696，397.1486 |
| P8 | Methylnissolin | 6.42 | [M+H]+ | C17H16O5 | 301.1054 | -5.5 | Flavonoids | AM | 167.0695，134.0346，301.1174，152.0458，301.1304，123.0429 |
| P9 | Glycitin | 5 | [M+H]+ | C22H22O10 | 447.1275 | -2.4 | Flavonoids | AM | 285.0751，270.0522，447.1287 |
| P10 | 7,4'-Dihydroxy-3'-methoxyisoflavone | 5.01 | [M+H]+ | C16H12O5 | 285.0749 | -3 | Flavonoids | PS | 285.0759，270.0513，242.0569 |
| P11 | 2-Methyl-3-hydroxyanthraquinone | 9.29 | [M-H]- | C15H10O3 | 237.0548 | -3.9 | Anthraquinones | HD | 237.0557，209.0611，236.0480，208.0531 |
| P12 | 2-Hydroxy-6-methylanthraquinone or 2-Hydroxy-7-methylanthraquinone | 9.69 | [M-H]- | C15H10O3 | 237.0536 | -8.9 | Anthraquinones | HD | 237.0550，209.0596，236.0475，237.0221，140.9951，195.0436 |
| P13 | Chrysophanol | 9.58 | [M-H]- | C15H10O4 | 253.0493 | -5.3 | Anthraquinones | RP | 253.0504，225.0557，224.0476，210.0315，173.0602 |
| P14 | 2-Hydroxy-1-methoxyanthraquinone | 8.15 | [M-H]- | C15H10O4 | 253.0492 | -5.7 | Anthraquinones | RP | 210.0322，238.0269，253.0504，182.0371 |
| P15 | 2-Hydroxy-3-hydroxymethylanthraquinone | 6.82 | [M-H]- | C15H10O4 | 253.0495 | -4.5 | Anthraquinones | HD | 253.0502，224.0472，225.0548，173.0239 |
| P16 | Emodin^S^ | 10.43 | [M-H]- | C15H10O5 | 269.0441 | -5.4 | Anthraquinones | RP | 269.0459，225.0559，241.0503，197.0606 |
| P17 | Aloe-emodin^S^ | 8.42 | [M-H]- | C15H10O5 | 269.0442 | -5 | Anthraquinones | RP | 269.0456，240.0429，239.0342，183.0448，116.9949，211.0393，241.0489，223.0390，268.0366 |
| P18 | Rhein^S^ | 9.76 | [M-H]- | C15H8O6 | 283.0238 | -3.6 | Anthraquinones | RP | 239.0344，183.0448，211.0397，265.1796，283.1901 |
| P19 | 6-Methyl-rhein | 9.58 | [M-H]- | C16H10O6 | 297.0394 | -3.6 | Anthraquinones | RP | 253.0610，225.0584，224.0474，297.0415，210.0319，254.0540，96.9605 |
| P20 | 8-Methyl chrysophanol | 8.16 | [M-H]- | C16H12O4 | 267.0653 | -1.5 | Anthraquinones | RP | 252.0431，223.0398，267.0658，195.045，0251.0348 |
| P21 | 2,6-Dihydroxy-1-methoxy-3-methylanthraquinone | 6.81 | [M-H]- | C16H12O5 | 283.06 | -4.2 | Anthraquinones | HD | 268.0376，211.0405，283.0630，239.0346，184.0530， |
| P22 | Chrysophanol-1-O-β-D-glucopyranoside or Chrysophanol-8-O-β-D-glucopyranoside | 6.67/6.81 | [M-H]- | C21H20O9 | 415.1015 | -4.7 | Anthraquinones | RP | 253.0507，162.8382，225.0546，277.0499，160.8416，266.0584 |
| P23 | Tryptophan | 3.14 | [M-H]- | C11H12N2O2 | 203.0824 | -1 | Amino acids | PS/HN | 116.0575，142.0677，203.0852，159.0935，130.0670，117.0541 |
| P24 | Histidine | 0.89 | [M-H]- | C6H9N3O2 | 154.0614 | -5.2 | Amino acids | HN | 93.0464，94.9252，154.0621，137.0350 |
| P25 | L-Methionine | 1.34 | [M+H]+ | C5H11NO2S | 150.058 | -2.2 | Amino acids | AM | 133.0318，104.0527，87.0260，102.0552，84.0441，150.0578，85.0273 |
| P26 | Proline | 0.96 | [M+H]+ | C5H9NO2 | 116.0702 | -3.5 | Amino acids | AM/HN | 116.0709 |
| P27 | Leucine or L-Isoleucine | 1.87 | [M+H]+ | C6H13NO2 | 132.1017 | -1.6 | Amino acids | AM/HN | 86.0964 |
| P28 | Scandoside or Deacetyl asperulosidic acid | 2.73 | [M-H]- | C16H22O11 | 389.1078 | -2.9 | Terpenoids | HD | 165.0560，389.1088，147.0456，139.0407，183.0668，89.0250，209.0459，227.0562，135.0456 |
| P29 | Danshenxinkun A or Tanshinone VI or Przewaquinone C | 9.59 | [M-H]- | C18H16O4 | 295.0962 | -4.7 | Terpenoids | SM | 237.0916，295.0977，265.0863，96.9604，249.0915，267.1024，238.0626，295.6093，222.0683，277.0875 |
| P30 | Miltionone I or Miltionone II | 7.96 | [M+H]+ | C19H20O4 | 313.1427 | -2.4 | Terpenoids | SM | 269.1523，171.0797，313.1429，253.0851，143.0849，199.0747，267.1370，169.0651，238.0618，254.1285 |
| P31 | Tanshinone V | 10.66 | [M+H]+ | C19H22O4 | 315.1582 | -2.8 | Terpenoids | SM | 297.1480，109.0641，97.0641，279.1378，315.1584，315.2312，251.1421，254.0928，268.1088，237.0893 |
| P32 | 1-(5-Hydroxym-ethyl-tetrahydro-furan-2-yl)-9H-β-carboline-3-carboxylic acid | 6.82 | [M-H]- | C17H12N2O4 | 307.0719 | -1.7 | Alkaloids | PS | 233.0715，205.0772，263.0824，96.9600，307.1200，191.0610，175.1801，307.0715，147.0451，204.0698，1660518 |
| P33 | 5-Oxo-pyrrolidine-2-carboxylic acid methyl ester | 2.85 | [M-H]- | C6H9NO3 | 142.0502 | -5.4 | Alkaloids | PS | 142.0510，114.0562 |
| P34 | Polygonatine A | 3.55 | [M+H]+ | C9H11NO2 | 166.0857 | -3.4 | Alkaloids | PS | 166.0861，136.0753，80.0488，118.0644，148.0753，120.0801，87.0430，108.0805，91.0546，103.0537 |
| P35 | Betaine | 0.92 | [M+H]+ | C5H11NO2 | 118.0858 | -3.9 | Alkaloids | AM | 118.086 |
| P36 | Cinnamic acid | 7.15 | [M-H]- | C9H8O2 | 147.0443 | -5.8 | Phenylpropanoids | RP | 103.0560，146.8984，147.0445，89.0233 |
| P37 | p-Coumaric acid | 5.28 | [M-H]- | C9H8O3 | 163.0397 | -2.3 | Phenylpropanoids | HD | 119.0594，93.0358，117.0357，91.0560，120.0540 |
| P38 | Scopoletin | 5.33 | [M+H]+ | C10H8O4 | 193.0489 | -3.3 | Phenylpropanoids | HD | 193.0485，133.0277，178.0250，122.0354，94.0409，150.0315，137.0587 |
| P39 | Esculetin | 4.33 | [M+H]+ | C9H6O4 | 179.0334 | -2.7 | Phenylpropanoids | HD | 179.0340，123.0433，133.0280，105.0336，151.0383，179.0442，91.0537，151.0608，89.0379，151.0942 |
| P40 | Citric acid | 2.54 | [M-H]- | C6H8O7 | 191.0196 | -0.7 | Others | RP | 87.0097，111.0113，85.0301，129.0195，191.0191 |
| P41 | p-Hydroxybenzoic acid | 7.24 | [M-H]- | C7H6O3 | 137.0239 | -3.8 | Others | HD/RP/SM | 93.0431，137.0261，94.0387 |
| P42 | 4-Vinyl-2-methoxyphenol | 6.99 | [M-H]- | C9H10O2 | 149.06 | -5.4 | Others | HD | 149.0612，105.0717 |
| P43 | 4-Vinyl-2-methoxyphenol | 2.65 | [M-H]- | C9H12N2O6 | 243.0612 | -4.4 | Others | HN | 110.0249，82.0303，143.1188，171.1134，122.0241，130.0507，152.0356，200.0566，181.1346，243.1335 |
| P44 | 1-O-Hexadecyl-sn-glycero-3-phosphocholine | 11.83 | [M+H]+ | C24H52NO6P | 482.3593 | -2.5 | Others | HN | 104.1069，482.3602，184.0732，86.0961，124.9995 |
| P45 | 1-O-Octadecanoyl-sn-glycero-3-phosphocholine | 13.11 | [M+H]+ | C26H54NO7P | 524.3697 | -2.6 | Others | HN | 184.0740，104.1070，524.3736，86.0962，506.3599，124.9994，341.3041 |
| P46 | Indole-3-carbaldehyde | 3.11 | [M+H]+ | C9H7NO | 146.0596 | -3 | Others | HN | 118.0650，91.0539，117.0568，146.0598，89.0378，90.0453，128.0497 |
| P47 | 2-Piperidone | 2.88 | [M+H]+ | C5H9NO | 100.0753 | -3.9 | Others | HN | 100.0751，82.0642 |
| P48 | Stigmasta-5,22-diene-3β-7α-diol or Stigmasta-5,22-diene-3β-7β-diol | 16.2 | [M+H]+ | C29H48O2 | 429.3706 | -4.9 | Others | HD | 429.3704，165.0900，163.1104，191.1055，177.0901，205.1204，149.0951，219.1366，95.0845 |
| P49 | Dibutyl phthalate | 12.11 | [M+H]+ | C16H22O4 | 279.158 | -3.9 | Others | HD/RP | 149.0230，121.0282，93.0330 |
| P50 | 1-O-Hexadecanoyl-sn-glycero-3-phosphocholine | 11.46 | [M+H]+ | C24H50NO7P | 496.3386 | -2.4 | Others | HN | 184.0739，104.1071，496.3423，86.0963，478.3288，124.9996，313.2732 |
| P51 | (E)-4-[5-(Hydroxymethyl)-2-furanyl]-3-butene-2-one | 6.5 | [M+H]+ | C9H10O3 | 167.0696 | -4 | Others | SM | 167.0699，105.0336，152.0467，106.0412，134.0361，123.0440，84.9593，151.0382，149.0587，124.0512 |

^S^ means compared with standard substances.

**Table S3** Metabolites of ZJTSD in serum.

| **Source** | **No** | **Rt** | **Adduct** | **Formula** | **Found at Mass** | **Error** | **Fragments** | **Reaction** |
| --- | --- | --- | --- | --- | --- | --- | --- | --- |
|  |  | **(min)** |  |  |  | **(ppm)** |  |  |
| **Flavonoids** | | | | | | | | |
| Calycosin | M1 | 9.01 | [M-H]^-^ | C_16_H_12_O_8_S | 363.0166 | -3.9 | 283.0608，268.0376，207.0450，224.0483，363.0168，251.0340，179.0492 | Sulfate Conjugation [M-H]^-^ |
|  | M2 | 7.41 | [M-H]^-^ | C_15_H_10_O_3_ | 237.0547 | -4.3 | 237.0554，209.0601，107.0503，236.0469，149.0611 | Loss of CH_2_O and O [M-H]^-^ |
|  | M3 | 9.19 | [M-H]^-^ | C_15_H_10_O_6_S | 317.0121 | -1.4 | 237.0552，193.0665，256.9836，96.9658，158.5557，228.9895，96.9699，248.9951 | Loss of CH_2_O and O+Sulfate Conjugation [M-H]^-^ |
| Genistin | M4 | 9.19 | [M-H]^-^ | C_15_H_10_O_6_S | 317.0121 | -1.4 | 237.0552，193.0665，256.9836，96.9658，158.5557，228.9895，96.9699，248.9951 | Loss of O and C_6_H_10_O_6_+Sulfate Conjugation [M-H]^-^ |
|  | M5 | 2.63 | [M-H]^-^ | C_6_H_12_O_4_ | 147.0653 | -6.7 | 85.0298，87.0093，101.0610，87.0285，129.0561，87.0196，147.0658，147.0495 | Loss of C_15_H_8_O_5_ and O [M-H]^-^ |
|  | M6 | 5.24 | [M-H]^-^ | C_21_H_18_O_11_ | 445.076 | -3.7 | 269.0459，240.0425，113.0250，85.0298 | Ketone Formation [M-H]^-^ |
|  | M7 | 6.75 | [M-H]^-^ | C_15_H_10_O_3_ | 237.0549 | -3.5 | 237.0563，209.0610，236.0476，208.0523 | Loss of O and C_6_H_10_O_6_ [M-H]^-^ |
| Isomucronulatol | M8 | 8.22 | [M-H]^-^ | C_17_H_18_O_8_S | 381.0629 | -5.4 | 301.1082，135.0451，271.0604，286.0840，381.0639，109.0294，121.0291，164.0474，335.2195，173.0015 | Sulfate Conjugation [M-H]^-^ |
|  | M9 | 6.49 | [M-H]^-^ | C_23_H_26_O_11_ | 477.1388 | -3 | 301.1095，113.0252，85.0300，477.1414，286.0846，99.0093，135.0456，175.0254，95.0143，271.0607 | Glucuronidation [M-H]^-^ |
| Quercetin | M10 | 7.3 | [M-H]^-^ | C_21_H_18_O_13_ | 477.0661 | -2.9 | 301.0355，477.0662，226.8740 | Glucuronidation [M-H]^-^ |
| Naringenin | M11 | 7.93 | [M-H]^-^ | C_15_H_14_O_4_ | 257.0808 | -4.4 | 257.0824，108.0224，109.0299，136.0173，80.0274，91.0194，135.0094，93.0349，239.0715 | Loss of H-2O [M-H]^-^ |
|  | M12 | 5.11 | [M-H]^-^ | C_21_H_20_O_10_ | 431.0981 | -0.6 | 255.0669、113.0250、149.0253、85.0301、175.0260、431.0985、99.0091、95.0142、135.0091、117.0198、103.0041、87.0094 | Loss of O+Glucuronidation [M-H]^-^ |
| Rutin | M13 | 7.3 | [M-H]^-^ | C_21_H_18_O_13_ | 477.0661 | -2.9 | 301.0355，477.0662，226.8740 | Loss of C6H10O5+Demethylation to Carboxylic Acid [M-H]^-^ |
| Neoisoliquiritigenin | M14 | 6.5 | [M+H]^+^ | C_15_H_14_O_4_ | 259.0958 | -2.7 | 121.0644，149.0593，91.0537，137.0593，103.0537，93.0694，165.0544，119.0482 | Loss of C_6_H_10_O_6_+Internal Hydrolysis [M+H]^+^ |
| Tectoridin | M15 | 6.72 | [M+H]^+^ | C_16_H_12_O_5_ | 285.075 | -2.6 | 285.0756，270.0517，242.0567，197.0585，225.0547 | Loss of C_6_H_10_O_6_ [M+H]^+^ |
|  | M16 | 7.6 | [M+H]^+^ | C_15_H_10_O_5_ | 271.0594 | -2.6 | 271.0598，153.0173，215.0689，91.0536，243.0632 | Loss of C_6_H_10_O_6_ and CH_2_ [M+H]^+^ |
| Liquiritin | M17 | 6.57 | [M+H]^+^ | C_15_H_10_O_4_ | 255.0645 | -2.7 | 255.0650、199.0750、137.0229、181.0637 | Loss of C_6_H_10_O_6_+Ketone Formation [M+H]^+^ |
|  | M18 | 5.45 | [M+H]^+^ | C_15_H_12_O_4_ | 257.0799 | -3.7 | 123.0348、107.0487、257.0812、95.0487、163.0385、137.0233、135.0433、147.0434 | Loss of C_6_H_10_O_5_ [M+H]^+^ |
| Complanatuside | M19 | 6.72 | [M+H]^+^ | C_16_H_12_O_5_ | 285.075 | -2.6 | 285.0756、270.0517、242.0567 | Loss of C_6_H_10_O_6_ and C_6_H_10_O_5_ [M+H]^+^ |
| Ononin | M20 | 6.72 | [M+H]^+^ | C_16_H_12_O_5_ | 285.075 | -2.6 | 285.0756，270.0517，242.0567，197.0585，225.0547 | Loss of C_6_H_10_O_5_+Oxidation [M+H]^+^ |
| Glycitin | M21 | 5.68 | [M+H]^+^ | C_16_H_12_O_5_ | 285.075 | -2.6 | 285.0754，270.0511，137.0222，253.0495，214.0611，225.0545，213.0542，134.0350 | Loss of C_6_H_10_O_5_ [M+H]^+^ |
| Apigenin | M22 | 6.77 | [M+H]^+^ | C_21_H_18_O_9_ | 415.1014 | -2.3 | 239.0700，85.0282，211.0751，113.0230 | Loss of O and O+Glucuronidation [M+H]^+^ |
|  | M23 | 6.72 | [M+H]^+^ | C_16_H_12_O_5_ | 285.075 | -2.6 | 285.0756，270.0517，242.0567，197.0585，225.0547 | Methylation [M+H]^+^ |
| Isoscutellarein | M24 | 6.72 | [M+H]^+^ | C_16_H_12_O_5_ | 285.075 | -2.6 | 285.0756，270.0517，242.0567，197.0585，225.0547，118.0403，229.0847，213.0540 | Loss of O+Methylation [M+H]^+^ |
| 2-(2-Hydroxypropyl)-5-methyl-7-hydroxychromone-7-*O*-*β*-D-glucoside | M25 | 5.1 | [M+H]^+^ | C_12_H_10_O_5_ | 235.0595 | 0.4 | 189.0545，217.0501，161.0579 | Loss of C_6_H_10_O_5_+Demethylation and Methylene to Ketone [M+H]^+^ |
| **Anthraquinones** | | | | | | | | |
| 2-Hydroxy-3-methoxy-7-methylanthraquinone | M26 | 9.68 | [M-H]^-^ | C_15_H_10_O_6_S | 317.0118 | -2.3 | 237.0570，209.0614 | Loss of CH_2_O+Sulfate Conjugation [M-H]^-^ |
| 2-Methyl-3-hydroxyanthraquinone | M27 | 9.58 | [M-H]^-^ | C_15_H_10_O_4_ | 253.0493 | -5.3 | 253.0504，225.0557，224.0476，210.0315，173.0602，182.0364，232.9844，254.0524 | Oxidation [M-H]^-^ |
|  | M28 | 9.08 | [M-H]^-^ | C_15_H_8_O_5_ | 267.0288 | -4.1 | 223.0436，195.0475，267.0303 | Demethylation to Carboxylic Acid [M-H]^-^ |
| 2-Hydroxy-3-methoxy-6-methylanthraquinone | M29 | 9.2 | [M-H]^-^ | C_16_H_12_O_7_S | 347.0213 | -5.2 | 252.0434，267.0662，224.0473 | Sulfate Conjugation [M-H]^-^ |
| Rhein | M30 | 10.57 | [M-H]^-^ | C_16_H_10_O_6_ | 297.0391 | -4.6 | 210.0327，238.0278，297.0400，253.0501，96.9604 | Methylation [M-H]^-^ |
|  | M31 | 7.48 | [M-H]^-^ | C_15_H_10_O_5_ | 269.045 | -2 | 269.0467，225.0565，241.0514，157.0238，197.0614，89.0243 | Loss of O+Hydrogenation [M-H]^-^ |
| Emodin | M32 | 9.62 | [M-H]^-^ | C_15_H_10_O_8_S | 349 | -6.8 | 254.0218，269.0449，226.0277，198.0323 | Sulfate Conjugation [M-H]^-^ |
|  | M33 | 9.68 | [M-H]^-^ | C_15_H_10_O_6_S | 317.0112 | -4.2 | 237.0557 | Loss of O and O+Sulfate Conjugation [M-H]^-^ |
|  | M34 | 11.43 | [M-H]^-^ | C_15_H_8_O_7_ | 299.018 | -5.8 | 255.0291，227.0345，226.0266，299.0185，183.0445，171.0450 | Demethylation to Carboxylic Acid [M-H]^-^ |
|  | M35 | 6.91 | [M-H]^-^ | C_21_H_18_O_11_ | 445.0759 | -3.9 | 269.0461，445.1750，445.0765，225.0563，113.0247，270.0485，85.0296 | Glucuronidation [M-H]^-^ |
|  | M36 | 10.6 | [M-H]^-^ | C_15_H_10_O_7_S | 333.0058 | -5 | 253.0504，225.0557，209.0603 | Loss of O+Sulfate Conjugation [M-H]^-^ |
|  | M37 | 6.81 | [M-H]^-^ | C_16_H_12_O_5_ | 283.06 | -4.2 | 268.0376，211.0405，283.0630，239.0346，184.0530，96.9602，135.0089，240.0421，148.0164 | Methylation [M-H]^-^ |
|  | M38 | 9.59 | [M-H]^-^ | C_15_H_10_O_4_ | 253.0494 | -4.9 | 253.0511，225.0557，224.0481，210.0320，182.0368 | Loss of O [M-H]^-^ |
|  | M39 | 6.8 | [M-H]^-^ | C_21_H_18_O_10_ | 429.0825 | -0.5 | 253.0534，113.0251，429.0834，85.0302，254.0547 | Loss of O+Glucuronidation [M-H]^-^ |
| Chrysophanol | M40 | 9.68 | [M-H]^-^ | C_15_H_10_O_6_S | 317.0118 | -2.3 | 237.0570，209.0614 | Loss of O+Sulfate Conjugation [M-H]^-^ |
| Physcion | M41 | 5.38 | [M-H]^-^ | C_22_H_20_O_11_ | 459.0929 | -0.9 | 283.0623，268.0387，113.0248，240.0438，85.0302，99.0090，175.0251，95.0138，103.0044，284.0629，117.0204 | Glucuronidation [M-H]^-^ |
|  | M42 | 9.2 | [M-H]^-^ | C_16_H_12_O_7_S | 347.0213 | -5.2 | 252.0434，267.0662，224.0473 | Loss of O+Sulfate Conjugation [M-H]^-^ |
|  | M43 | 9.69 | [M-H]^-^ | C_15_H_10_O_3_ | 237.0536 | -8.9 | 237.0550，209.0596，236.0475，237.0221，140.9951，195.0436 | Loss of CH_2_O and O [M-H]^-^ |
|  | M44 | 6.85 | [M-H]^-^ | C_16_H_14_O_5_ | 285.076 | -3 | 135.0094，91.0196，285.0776，270.0532 | Hydrogenation [M-H]^-^ |
| Laccaic acid D | M45 | 5.59 | [M-H]^-^ | C_22_H_18_O_12_ | 473.071 | -3.3 | 253.0508，297.0403，113.0248，85.0294 | Loss of O+Glucuronidation [M-H]^-^ |
|  | M46 | 7.41 | [M-H]^-^ | C_16_H_10_O_5_ | 281.0443 | -4.5 | 237.0556，209.0612，236.0484 | Loss of O and O [M-H]^-^ |
| 8-Methyl chrysophanol | M47 | 9.07 | [M-H]^-^ | C_16_H_12_O_7_S | 347.0214 | -4.9 | 267.0675，252.0433，347.0224，251.0351 | Sulfate Conjugation [M-H]^-^ |
|  | M48 | 6.81 | [M-H]^-^ | C_16_H_12_O_5_ | 283.06 | -4.2 | 268.0376，211.0405，283.0630，239.0346，184.0530，96.9602，135.0089，240.0421，148.0164 | Oxidation [M-H]^-^ |
|  | M49 | 6.81 | [M-H]^-^ | C_16_H_12_O_5_ | 283.06 | -4.2 | 268.0376，211.0405，283.0630，239.0346，184.0530，96.9602，135.0089，240.0421，148.0164 | Oxidation [M-H]^-^ |
| Rhein-8-*O*-*β*-D-glucopyranoside | M50 | 9.08 | [M-H]^-^ | C_15_H_8_O_5_ | 267.0288 | -4.1 | 223.0436，195.0475，267.0303 | Loss of C_6_H_10_O_6_ [M-H]^-^ |
|  | M51 | 2.46 | [M-H]^-^ | C_5_H_8_O_5_ | 147.0296 | -2 | 87.0095，85.0300，147.0301，103.0409，129.0194 | Loss of C_15_H_6_O_6_ and O+Demethylation and Methylene to Ketone [M-H]^-^ |
| Aloe-emodin-8-*O*-*β*-D-glucopyranoside | M52 | 9.01 | [M-H]^-^ | C_14_H_8_O_2_ | 207.0446 | -2.7 | 179.0508、207.0455、151.0553、197.0605 | Loss of C_6_H_10_O_6_ and O+Loss of Hydroxymethylene [M-H]^-^ |
|  | M53 | 6.59 | [M-H]^-^ | C_15_H_10_O_4_ | 253.0503 | -1.3 | 253.0617，91.0198，224.0483，133.0301，208.0536，132.0223，223.0406，209.0615，135.0093，180.0583 | Loss of C_6_H_10_O_6_ [M-H]^-^ |
| Aloe-emodin-ω-*O*-*β*-D-glucopyranoside | M54 | 6.81 | [M-H]^-^ | C_16_H_12_O_5_ | 283.06 | -4.2 | 268.0376，211.0405，283.0630，239.0346，184.0530，96.9602，135.0089，240.0421，148.0164 | Loss of C_6_H_10_O_5_+Methylation [M-H]^-^ |
|  | M55 | 6.71 | [M-H]^-^ | C_16_H_12_O_5_ | 283.0601 | -3.9 | 268.0379，240.0434，283.0624，211.0404，184.0532，239.0349，196.0530，267.0290，212.0468 | Loss of C_6_H_10_O_5_+Methylation [M-H]^-^ |
|  | M56 | 6.59 | [M-H]^-^ | C_15_H_10_O_4_ | 253.0503 | -1.3 | 253.0617，91.0198，224.0483，133.0301，208.0536，132.0223，223.0406，209.0615，135.0093，180.0583 | Loss of C_6_H_10_O_6_ [M-H]^-^ |
|  | M57 | 9.08 | [M-H]^-^ | C_15_H_8_O_5_ | 267.0288 | -4.1 | 223.0436，195.0475，267.0303 | Loss of C_6_H_10_O_6_+Ketone Formation [M-H]^-^ |
|  | M58 | 8.13 | [M-H]^-^ | C_16_H_12_O_4_ | 267.0651 | -4.4 | 252.0445，223.0403，267.0674，251.0351，195.0455，132.0217，135.0089，224.0473，91.0195，208.0528 | Loss of C_6_H_10_O_6_+Methylation [M-H]^-^ |
| 6-Methyl-rhein | M59 | 6.91 | [M-H]^-^ | C_22_H_18_O_12_ | 473.071 | -3.3 | 253.0551，297.0410，473.0730，254.0543，113.0248 | Glucuronidation [M-H]^-^ |
|  | M60 | 7.41 | [M-H]^-^ | C_16_H_10_O_5_ | 281.0443 | -4.5 | 237.0556，209.0612，236.0484，96.9600 | Loss of O [M-H]^-^ |
| Emodin-8-*O*-β-D-(6-*O*-acetyl)-glucoside | M61 | 9.58 | [M-H]^-^ | C_15_H_10_O_4_ | 253.0493 | -5.3 | 253.0504，225.0557，224.0476，210.0315，173.0602，182.0364，232.9844，254.0524 | Loss of C_8_H_12_O_7_ [M-H]^-^ |
|  | M62 | 5.05 | [M-H]^-^ | C_8_H_14_O_5_ | 189.0759 | -5 | 129.0564，99.0819，127.0767，189.0761，145.0870，171.0664 | Loss of C_15_H_8_O_5_ and O [M-H]^-^ |
| Chrysophanol-8-O-(6-O-malonyl)-glucoside | M63 | 9.69 | [M-H]^-^ | C_15_H_10_O_3_ | 237.0536 | -8.9 | 237.0550，209.0596，236.0475，237.0221，140.9951，195.0436 | Loss of C_9_H_12_O_9_ [M-H]^-^ |
| Emodin-8-*O*-(6-*O*-malonyl)-glucoside | M64 | 8.22 | [M-H]^-^ | C_15_H_8_O_6_ | 283.0232 | -5.7 | 239.0343，211.0399，283.0234，183.0445，240.0416，167.0500，283.0473 | Loss of C_9_H_12_O_9_+Demethylation to Carboxylic Acid [M-H]^-^ |
|  | M65 | 6.81 | [M-H]^-^ | C_16_H_12_O_5_ | 283.06 | -4.2 | 268.0376，211.0405，283.0630，239.0346，184.0530，96.9602，135.0089，240.0421，148.0164 | Loss of C_9_H_12_O_8_+Methylation [M-H]^-^ |
|  | M66 | 1.18 | [M-H]^-^ | C_6_H_10_O_7_ | 193.0345 | -4.6 | 158.8468，85.0298.，113.0251，135.0295，193.8156，115.0037，101.0245，95.0141 | Loss of C_15_H_8_O_4_ and C_3_H_2_O_4_+Demethylation to Carboxylic Acid [M-H]^-^ |
| 2,3-Dimethoxy-6-methylanthraquinone | M67 | 9.2 | [M-H]^-^ | C_16_H_12_O_7_S | 347.0213 | -5.2 | 252.0434，267.0662，224.0473 | Loss of CH_2_+Sulfate Conjugation [M-H]^-^ |
| 1,3,8-Trihydroxy-6-hydroxymethylanthraquinone | M68 | 6.82 | [M+H]^+^ | C_16_H_12_O_5_ | 285.0748 | -3.3 | 285.0753、270.0519、137.0231、213.0541、253.0486、225.0542、134.0356、214.0618、269.0441、197.0594 | Loss of O+Methylation [M+H]^+^ |
| **Amino acids** | | | | | | | | |
| Phenylalanine | M69 | 6.99 | [M-H]^-^ | C_9_H_10_O_2_ | 149.0599 | -6 | 149.0612，105.0717，92.0271 | Loss of NH [M-H]^-^ |
|  | M70 | 2.8 | [M-H]^-^ | C_15_H_21_NO_7_ | 326.1238 | -2.2 | 147.0493，164.0816，103.0571，101.0254，144.0826，206.0832 | Glucose Conjugation [M-H]^-^ |
| **Terpenoids** | | | | | | | | |
| (*E*)-6-*O*-*p*-Feruloyl scandoside methyl ester | M71 | 7.33 | [M-H]^-^ | C_20_H_20_O_8_ | 387.1066 | -5 | 211.0614，175.0404，160.0169，134.0375，193.0501，149.0606，165.0554，150.0324 | Loss of CH_2_O and C_6_H_10_O_5_ [M-H]^-^ |
|  | M72 | 4.51 | [M-H]^-^ | C_16_H_18_O_10_ | 369.081 | -4.7 | 193.0503，113.0248，178.0269，85.0298，134.0373，369.0829，96.9600，99.0084 | Loss of C_17_H_22_O_10_+Glucuronidation [M-H]^-^ |
| (*E*)-6-*O*-Coumaroyl scandoside methyl ester-10-*O*-methyl ether | M73 | 6.88 | [M-H]^-^ | C_17_H_24_O_8_ | 355.1384 | -4 | 164.0846，179.1080，113.0245，85.0296，355.1383，99.0091，117.0195 | Loss of C_9_H_6_O_3_ and O+Loss of Hydroxymethylene [M-H]^-^ |
| Asperulosidic acid | M74 | 5.7 | [M-H]^-^ | C_10_H_12_O_7_S | 275.0224 | -2.6 | 195.0674，136.0535，275.0234，135.0455，108.0221，123.0456，151.0767 | Loss of C_6_H_10_O_6_ and C_2_H_2_O_2_+Sulfate Conjugation [M-H]^-^ |
|  | M75 | 4.29 | [M-H]^-^ | C_17_H_20_O_11_ | 399.0932 | -0.2 | 223.0621，208.0383，164.0483，113.0247，85.0300，149.0251，193.0148，96.9607，99.0087 | Loss of O and O+Demethylation and Methylene to Ketone [M-H]^-^ |
| Danshenxinkun B | M76 | 10.29 | [M-H]^-^ | C_18_H_20_O_3_ | 283.1325 | -5.2 | 255.1394，283.1337，239.1071，240.1149，221.1909，225.0903，265.1817，283.1609 | Hydrogenation [M-H]^-^ |
| Tormentic acid | M77 | 11.24 | [M-H]^-^ | C_30_H_46_O_6_ | 501.3202 | -3.9 | 455.3169,501.2894,456.3200 | Ketone Formation [M-H]^-^ |
|  | M78 | 11.09 | [M-H]^-^ | C_36_H_56_O_9_ | 631.3823 | -4.5 | 631.3839，455.3521，175.0247，113.0239，89.0243，112.9859，85.0297，632.3788，113.0092 | Loss of O and O+Glucuronidation [M-H]^-^ |
| Polygonoide C | M79 | 2.86 | [M-H]^-^ | C_6_H_12_O_4_ | 147.0656 | -4.6 | 147.0667、87.0089、99.0446、129.0556、85.0298、89.0241、83.0499、98.9890 | Loss of C_42_H_66_O_15_ [M-H]^-^ |
|  | M80 | 3.47 | [M-H]^-^ | C_12_H_22_O_9_ | 309.1179 | -3.9 | 129.0560、115.0765、127.0768、189.0771、309.1183、85.0291、96.9599 | Loss of C_36_H_56_O_10_ [M-H]^-^ |
|  | M81 | 6.19 | [M-H]^-^ | C_7_H_14_O_3_ | 145.0869 | -0.8 | 99.0822、145.0882、97.0666、80.9651 | Loss of C_42_H_66_O_15_ and O+Methylation [M-H]^-^ |
| **Phenylpropanoids** | | | | | | | | |
| Esculetin | M82 | 5.27 | [M+H]^+^ | C_9_H_6_O_2_ | 147.0436 | -3.1 | 91.0535，119.0488，147.0435 | Loss of O and O [M+H]^+^ |
|  | M83 | 5.31 | [M+H]^+^ | C_9_H_8_O_3_ | 165.0541 | -3.2 | 91.0540，147.0440，119.0489 | Loss of H-2O [M+H]^+^ |
| **Others** | | | | | | | | |
| Lindleyin | M84 | 3.58 | [M-H]^-^ | C_13_H_16_O_9_ | 315.0709 | -4 | 108.0223，152.0115，315.0721，109.0297，153.0192，151.0414 | Loss of C_10_H_10_O_2_ [M-H]^-^ |
|  | M85 | 6.49 | [M-H]^-^ | C_10_H_12_O_5_S | 243.0334 | 0.5 | 163.0801，243.0369，80.9659，119.0507，164.0808 | Loss of C_13_H_14_O_9_+Sulfate Conjugation [M-H]^-^ |
| Isolindleyin | M86 | 5.33 | [M-H]^-^ | C_8_H_8_O_4_ | 167.0343 | -4.1 | 108.0221，152.0113，123.0441，167.0598，120.9988，167.0346，95.0136，80.0268 | Loss of C_16_H_20_O_7_+Methylation [M-H]^-^ |
| Adenosine | M87 | 2.74 | [M-H]^-^ | C_4_H_8_O_3_ | 103.0397 | -3.6 | 103.0405，85.0297，101.0249 | Loss of C_5_H_3_N_5_+Loss of Hydroxymethylene [M-H]^-^ |
| *p*-Hydroxybenzoic acid | M88 | 3.72 | [M-H]^-^ | C_13_H_14_O_9_ | 313.0555 | -3.2 | 137.0249，136.0165，113.0246，85.0298，313.0569，99.0089，108.0219 | Glucuronidation [M-H]^-^ |
|  | M89 | 6.82 | [M-H]^-^ | C_8_H_8_O_3_ | 151.0393 | -5.1 | 92.0272，136.0165，151.0399，108.0211 | Methylation [M-H]^-^ |
| Citric acid | M90 | 2.71 | [M-H]^-^ | C_6_H_8_O_6_ | 175.0244 | -2.3 | 87.0243，113.0251，85.0303，88.0127，157.0153 | Loss of O [M-H]^-^ |
| Cinnamic acid | M91 | 5.28 | [M-H]^-^ | C_9_H_8_O_3_ | 163.0397 | -2.3 | 119.0594，93.0358，117.0357，91.0560，120.0540，163.0409，104.0274 | Oxidation [M-H]^-^ |
| Gallic acid | M92 | 6.82 | [M-H]^-^ | C_8_H_8_O_3_ | 151.0393 | -5.1 | 92.0272、136.0165、151.0399、108.0211、91.0188、121.0281 | Loss of O and O+Methylation [M-H]^-^ |
|  | M93 | 4.87 | [M-H]^-^ | C_8_H_8_O_5_ | 183.0298 | -0.6 | 124.0172、168.0065、123.0091、95.0141、123.0216、83.3086、183.0074、109.0163、116.9283、80.9647、97.0289、96.9586、 | Methylation [M-H]^-^ |
| Protocatechualdehyde | M94 | 5.77 | [M-H]^-^ | C_6_H_6_O_2_ | 109.0293 | -1.8 | 109.0303，108.0224，91.0194，81.0347 | Loss of CO [M-H]^-^ |
|  | M95 | 4.78 | [M-H]^-^ | C_7_H_6_O_2_ | 121.0296 | 0.8 | 92.0274，121.0306，120.0225，93.0346，91.0193 | Loss of O [M-H]^-^ |
| Rosmarinic acid | M96 | 5.71 | [M-H]^-^ | C_9_H_10_O_6_S | 245.0117 | -3.4 | 165.0555、93.0351、245.0135、121.0660、80.9656 | Loss of C_9_H_6_O_4_ and O+Sulfate Conjugation [M-H]^-^ |
|  | M97 | 3.91 | [M-H]^-^ | C_15_H_16_O_9_ | 339.0719 | -0.8 | 119.0508、163.0408、113.0252、85.0302、99.0093、95.0144、229.0518、87.0092、124.0080、175.0246、117.0200、 | Loss of C_9_H_8_O_5_+Glucuronidation [M-H]^-^ |
| 4,4'-Dihydroy-*α*-truxillic acid | M98 | 8.12 | [M+H]^+^ | C_19_H_18_O_5_ | 327.1216 | -3.4 | 309.1125，265.1215，223.0739，250.0980，236.0828，247.1111，167.0852，195.0797 | Loss of O+Methylation [M+H]^+^ |
| 1-*O*-Hexadecyl-*sn*-glycero-3-phosphocholine | M99 | 10.76 | [M+H]^+^ | C_23_H_48_NO_7_P | 482.3227 | -2.9 | 184.0729，482.3228，104.1068，86.0962，464.3121，124.9994 | Demethylation and Methylene to Ketone [M+H]^+^ |
|  | M100 | 12.35 | [M+H]^+^ | C_24_H_50_NO_7_P | 496.3385 | -2.6 | 184.0725，496.3398，104.1066，86.0956，478.3261，124.9978 | Ketone Formation [M+H]^+^ |
| 1-*O*-Hexadecanoyl-*sn*-glycero-3-phosphocholine | M101 | 10.83 | [M+H]^+^ | C_24_H_48_NO_7_P | 494.3224 | -3.5 | 184.0730，494.3237，104.1067，86.0960，476.3131，124.9990 | Desaturation [M+H]^+^ |
|  | M102 | 10.47 | [M+H]^+^ | C_24_H_48_NO_7_P | 494.3223 | -3.7 | 184.0732，494.3238，104.1068，86.0963，476.3127，124.9994，311.2573 | Desaturation [M+H]^+^ |
| 1-*O*-Octadecanoyl-*sn*-glycero-3-phosphocholine | M103 | 8.28 | [M+H]^+^ | C_24_H_50_NO_8_P | 512.3327 | -3.9 | 512.3327，184.0722，104.1060，351.2306，512.2704，86.0955，512.2901，323.2344 | Ethyl to Alcohol [M+H]^+^ |
|  | M104 | 11.74 | [M+H]^+^ | C_26_H_54_NO_8_P | 540.3634 | -4.8 | 104.1068，540.3652，184.0731，86.0959，522.3537 | Oxidation [M+H]^+^ |
|  | M105 | 12.67 | [M+H]^+^ | C_26_H_52_NO_7_P | 522.35542 | -5 | 184.0726，522.3542，104.1064，86.0959，337.2732，504.3424，124.9991 | Desaturation [M+H]^+^ |

**Table S4** Pharmacokinetic parameters for prototype compounds of ZJTSD absorbed into rat serum (mean±SD, *n*=3).

| **No** | **Component Name** | **t_1/2_** | **T_max_** | **AUC_(0→t)_** | **MRT_(0→t)_** | **C_max_** |
| --- | --- | --- | --- | --- | --- | --- |
| P1 | 2,5-Dimethyl-7-hydroxychromone | 2.70±0.14 | 0.67±0.29 | 24671.06±4893.91 | 3.43±0.76 | 15742.70±4438.15 |
| P2 | 7-Hydroxy-2-(2-hydroxypropyl)-5-methylchromen-4-one | 2.17±0.12 | 0.67±0.29 | 12786.14±2018.04 | 3.45±0.58 | 6446.33±1297.42 |
| P3 | 3-Methoxy-5,7-dihydroxyflavone | 4.04±1.91 | 1.83±1.89 | 6057.85±629.10 | 4.80±0.38 | 1262.59±397.02 |
| P4 | 2-Methyl-5-carboxymethyl-7-hydroxychromone | 3.14±0.86 | 1.67±2.02 | 28.01±1.00 | 5.29±0.30 | 5.31±0.43 |
| P5 | 2-Methyl-5-acetonyl-7-hydroxychromone | 3.09±0.19 | 0.67±0.29 | 9.56±1.20 | 4.23±0.55 | 4.55±1.84 |
| P6 | 2,5-Dimethyl-7-methoxychromone | n.a. | 4.75±4.02 | 844.45±67.63 | 12.10±0.02 | 40.73±7.72 |
| P7 | 2-(2-Hydroxypropyl)-5-methyl-7-hydroxychromone-7-*O*-*β*-D-glucoside | 7.53±1.65 | 0.92±0.95 | 1.05±0.08 | 6.76±1.16 | 0.23±0.06 |
| P8 | Methylnissolin | 4.16±2.01 | 0.42±0.14 | 3.73±0.45 | 6.10±1.53 | 0.77±0.18 |
| P9 | Glycitin | 3.56±1.89 | 0.92±0.95 | 1.99±0.28 | 4.70±1.01 | 0.80±0.39 |
| P10 | 7,4'-Dihydroxy-3'-methoxyisoflavone | 67.26±4.40 | 2.08±1.88 | 13.77±1.30 | 10.76±0.72 | 2.24±0.54 |
| P11 | 2-Methyl-3-hydroxyanthraquinone | 4.29±2.71 | 0.67±0.29 | 42755.90±9960.20 | 3.23±0.59 | 27897.04±11297.76 |
| P12 | 2-Hydroxy-6-methylanthraquinone or 2-Hydroxy-7-methylanthraquinone | 5.43±2.61 | 3.50±2.78 | 24366.32±2635.93 | 4.73±0.33 | 4650.07±1389.32 |
| P13 | Chrysophanol | 2.42±1.63 | 1.67±2.02 | 119599.26±21102.35 | 4.89±0.61 | 26478.39±12536.14 |
| P14 | 2-Hydroxy-1-methoxyanthraquinone | 3.50±2.06 | 0.67±0.29 | 16829.25±2437.99 | 4.24±0.57 | 5433.35±2131.05 |
| P15 | 2-Hydroxy-3-hydroxymethylanthraquinone | 345.54±590.13 | 1.67±2.02 | 24336.71±1472.76 | 4.81±0.31 | 6536.62±1879.08 |
| P16 | Emodin | 8.19±9.39 | 2.83±2.02 | 24511.26±3448.36 | 5.84±0.37 | 5712.01±2179.35 |
| P17 | Aloe-emodin | 1.73 | 4.17±3.75 | 501.23±506.79 | 5.20±3.03 | 334.26±327.82 |
| P18 | Rhein | 4.70±0.90 | 1.67±2.02 | 12656.91±1942.37 | 3.55±0.57 | 6870.16±2791.69 |
| P19 | 6-Methyl-rhein | 1.98±0.38 | 1.67±2.02 | 406691.82±79143.95 | 5.64±1.31 | 79253.50±34585.36 |
| P20 | 8-Methyl chrysophanol | 3.38±0.96 | 0.67±0.29 | 49952.22±8257.37 | 4.30±0.34 | 15105.85±4254.91 |
| P21 | 2,6-Dihydroxy-1-methoxy-3-methylanthraquinone | 3.45±1.44 | 2.50±3.04 | 14708.19±3795.11 | 4.27±0.55 | 4131.99±286.40 |
| P22 | Chrysophanol-1-O-β-D-glucopyranoside or Chrysophanol-8-O-β-D-glucopyranoside | 4.10±0.92 | 1.67±2.02 | 1607.45±136.64 | 4.40±0.15 | 426.29±80.57 |
| P23 | Tryptophan | 131.22 | 1.08±0.88 | 2012763.31±100209.70 | 12.43±0.14 | 104174.99±9481.89 |
| P24 | Histidine | 21.08±10.21 | 2.75±2.95 | 14750.17±755.80 | 9.90±0.35 | 1046.16±106.70 |
| P25 | L-Methionine | 351.57 | 2.17±3.32 | 401.02±17.87 | 12.52±1.36 | 36.92±6.87 |
| P26 | Proline | 47.15±35.84 | 0.50±0.00 | 3484.27±348.48 | 11.09±0.44 | 214.89±12.40 |
| P27 | Leucine or L-Isoleucine | 39.21±14.92 | 6.00±5.29 | 4362.83±294.23 | 11.45±0.85 | 270.13±27.21 |
| P28 | Scandoside or Deacetyl asperulosidic acid | 2.23 | 8.67±3.06 | 208731.48±32504.77 | 8.65±0.48 | 23167.93±4947.45 |
| P29 | Danshenxinkun A or Tanshinone VI or Przewaquinone C | 7.45±10.10 | 2.83±2.02 | 9095.83±1796.53 | 5.22±0.93 | 1993.91±1192.27 |
| P30 | Miltionone I or Miltionone II | 4.45±0.20 | 4.08±3.88 | 521.43±158.54 | 9.88±0.52 | 45.62±16.77 |
| P31 | Tanshinone V | 7.21 | 5.33±2.31 | 269.12±44.99 | 5.85±0.84 | 44.36±4.60 |
| P32 | 1-(5-Hydroxym-ethyl-tetrahydro-furan-2-yl)-9*H*-*β*-carboline-3-carboxylic acid | 4.52±2.85 | 1.67±2.02 | 5222.12±741.24 | 5.02±0.68 | 1045.27±428.95 |
| P33 | 5-Oxo-pyrrolidine-2-carboxylic acid methyl ester | n.a. | 14.00±9.17 | 160424.78±16338.37 | 13.95±1.35 | 12565.22±634.77 |
| P34 | Polygonatine A | 3.76±1.03 | 1.67±2.02 | 117.75±6.93 | 3.96±0.32 | 35.51±8.93 |
| P35 | Betaine | 24.20±14.46 | 6.67±4.62 | 6286.82±800.59 | 10.79±0.64 | 389.87±46.95 |
| P36 | Cinnamic acid | 25.85±26.44 | 1.58±2.10 | 3280.21±316.50 | 5.15±0.25 | 600.60±104.12 |
| P37 | *p*-Coumaric acid | 3.23±0.95 | 1.58±2.10 | 127262.03±3099.06 | 3.97±0.59 | 39818.39±10320.78 |
| P38 | Scopoletin | 4.77±1.43 | 0.50±0.00 | 18.10±4.67 | 4.36±1.52 | 13.58±7.48 |
| P39 | Esculetin | 4.40±3.90 | 0.33±0.14 | 2.80±0.48 | 4.92±0.80 | 0.88±0.33 |
| P40 | Citric acid | 24.74±23.60 | 3.33±1.16 | 504519.83±33540.23 | 9.56±1.76 | 41107.85±12669.49 |
| P41 | *p*-Hydroxybenzoic acid | 19.43±2.90 | 2.75±2.17 | 231483.34±21437.88 | 9.15±0.95 | 23731.78±1990.36 |
| P42 | 4-Vinyl-2-methoxyphenol | n.a. | 10.67±2.31 | 49432.39±17484.37 | 10.20±0.64 | 3649.57±1387.48 |
| P43 | Uridine | n.a. | 8.00±3.46 | 73934.95±36803.09 | 11.50±3.62 | 5785.95±890.23 |
| P44 | 1-*O*-Hexadecyl-*sn*-glycero-3-phosphocholine | 33.10±13.26 | 0.58±0.38 | 7493.83±641.57 | 11.94±0.91 | 618.66±236.10 |
| P45 | 1-*O*-Octadecanoyl-*sn*-glycero-3-phosphocholine | 21.89±6.81 | 2.00±0.00 | 52614.90±1167.08 | 9.91±0.56 | 4124.44±162.46 |
| P46 | Indole-3-carbaldehyde | n.a. | 12.08±11.88 | 4088.93±199.28 | 12.72±0.30 | 203.79±14.24 |
| P47 | 2-Piperidone | n.a. | 12.00±0.00 | 4052.20±1002.08 | 11.79±0.21 | 470.02±123.49 |
| P48 | Stigmasta-5,22-diene-3β-7α-diol or Stigmasta-5,22-diene-3β-7β-diol | n.a. | 5.33±5.77 | 3352.76±2561.29 | 12.27±1.62 | 344.33±149.63 |
| P49 | Dibutyl phthalate | n.a. | 8.08±6.78 | 542.84±7.58 | 12.14±0.17 | 26.50±1.13 |
| P50 | 1-*O*-Hexadecanoyl-*sn*-glycero-3-phosphocholine | 21.93±12.84 | 1.00±0.87 | 149276.86±8351.53 | 10.19±0.58 | 11488.73±739.96 |
| P51 | (*E*)-4-[5-(Hydroxymethyl)-2-furanyl]-3-butene-2-one | n.a. | 4.67±1.16 | 25.83±4.47 | 4.20±0.25 | 5.71±2.54 |

n.a. the pharmacokinetic parameters are not available due to limited amounts of data points.

**Table S5** Pharmacokinetic parameters for metabolites of ZJTSD in serum (mean±SD, *n*=3).

| **No** | **Source** | **Reaction** | **t_1/2_** | **T_max_** | **AUC_(0→t)_** | **MRT_(0→t)_** | **C_max_** |
| --- | --- | --- | --- | --- | --- | --- | --- |
| M1 | Calycosin | Sulfate Conjugation [M-H]^-^ | 3.30±0.90 | 1.83±1.89 | 15844.89±4992.26 | 5.84±1.05 | 2666.13±1474.49 |
| M2 |  | Loss of CH_2_O and O [M-H]^-^ | 4.78±1.60 | 1.67±2.02 | 17373.26±1879.78 | 6.70±0.81 | 2379.54±261.38 |
| M3 |  | Loss of CH_2_O and O+Sulfate Conjugation [M-H]^-^ | 10.41±6.11 | 1.42±1.01 | 8974.15±1262.20 | 8.83±1.12 | 922.50±162.83 |
| M4 | Genistin | Loss of O and C_6_H_10_O_6_+Sulfate Conjugation [M-H]^-^ | 10.41±6.11 | 1.42±1.01 | 8974.15±1262.20 | 8.83±1.12 | 922.50±162.83 |
| M5 |  | Loss of C_15_H_8_O_5_ and O [M-H]^-^ | 10.66±10.81 | 1.67±2.02 | 6474.03±1921.75 | 7.30±1.87 | 982.96±195.81 |
| M6 |  | Ketone Formation [M-H]^-^ | 27.46±43.07 | 1.67±2.02 | 69472.32±15854.72 | 5.06±0.88 | 22100.97±11137.32 |
| M7 |  | Loss of O and C_6_H_10_O_6_ [M-H]^-^ | 3.07±0.05 | 5.33±2.31 | 55798.44±2666.82 | 6.71±0.73 | 8104.66±3784.77 |
| M8 | Isomucronulatol | Sulfate Conjugation [M-H]^-^ | 3.83±1.29 | 2.83±2.02 | 12088.34±1289.25 | 6.78±1.20 | 1657.59±762.60 |
| M9 |  | Glucuronidation [M-H]^-^ | 2.11±0.23 | 3.00±1.73 | 311670.19±23465.58 | 6.59±1.52 | 46364.19±21524.38 |
| M10 | Quercetin | Glucuronidation [M-H]^-^ | 9.07±2.88 | 2.67±1.16 | 25584.15±4484.87 | 8.41±0.47 | 2771.42±694.63 |
| M11 | Naringenin | Loss of H-2O [M-H]^-^ | 7.01±2.29 | 0.75±0.43 | 9713.17±2326.76 | 3.97±0.98 | 6257.87±2687.75 |
| M12 |  | Loss of O+Glucuronidation [M-H]^-^ | 39.74±33.44 | 2.08±1.88 | 9826.38±901.13 | 9.02±2.02 | 2085.17±1045.75 |
| M13 | Rutin | Loss of C6H10O5+Demethylation to Carboxylic Acid [M-H]^-^ | 9.07±2.88 | 2.67±1.16 | 25584.15±4484.87 | 8.41±0.47 | 2771.42±694.63 |
| M14 | Neoisoliquiritigenin | Loss of C_6_H_10_O_6_+Internal Hydrolysis [M+H]^+^ | 21.10±16.69 | 0.75±0.43 | 117.77±18.19 | 8.76±2.37 | 25.27±4.92 |
| M15 | Tectoridin | Loss of C_6_H_10_O_6_ [M+H]^+^ | 18.96±20.40 | 0.58±0.38 | 154.66±41.73 | 6.87±1.55 | 52.89±6.43 |
| M16 |  | Loss of C_6_H_10_O_6_ and CH_2_ [M+H]^+^ | 8.82±5.63 | 0.67±0.29 | 89.97±27.55 | 3.34±1.26 | 52.84±17.72 |
| M17 | Liquiritin | Loss of C_6_H_10_O_6_+Ketone Formation [M+H]^+^ | 16.61±13.75 | 0.67±0.29 | 683.65±170.19 | 7.71±1.64 | 190.98±45.56 |
| M18 |  | Loss of C_6_H_10_O_5_ [M+H]^+^ | 16.76±4.54 | 1.42±1.01 | 50.23±9.67 | 9.78±2.65 | 9.31±6.49 |
| M19 | Complanatuside | Loss of C_6_H_10_O_6_ and C_6_H_10_O_5_ [M+H]^+^ | 18.96±20.40 | 0.58±0.38 | 154.66±41.73 | 6.87±1.55 | 52.89±6.43 |
| M20 | Ononin | Loss of C_6_H_10_O_5_+Oxidation [M+H]^+^ | 18.96±20.40 | 0.58±0.38 | 154.66±41.73 | 6.87±1.55 | 52.89±6.43 |
| M21 | Glycitin | Loss of C_6_H_10_O_5_ [M+H]^+^ | 2.15±1.09 | 4.67±1.16 | 1145.69±417.72 | 5.95±1.67 | 169.10±57.59 |
| M22 | Apigenin | Loss of O and O+Glucuronidation [M+H]^+^ | 2.98±0.07 | 5.33±2.31 | 69.72±9.63 | 7.05±0.70 | 10.13±1.95 |
| M23 |  | Methylation [M+H]^+^ | 18.96±20.40 | 0.58±0.38 | 154.66±41.73 | 6.87±1.55 | 52.89±6.43 |
| M24 | Isoscutellarein | Loss of O+Methylation [M+H]^+^ | 18.96±20.40 | 0.58±0.38 | 154.66±41.73 | 6.87±1.55 | 52.89±6.43 |
| M25 | 2-(2-Hydroxypropyl)-5-methyl-7-hydroxychromone-7-O-β-D-glucoside | Loss of C_6_H_10_O_5_+Demethylation and Methylene to Ketone [M+H]^+^ | 3.11±0.91 | 1.67±2.02 | 28.01±2.43 | 5.27±0.35 | 5.31±0.43 |
| M26 | 2-Hydroxy-3-methoxy-7-methylanthraquinone | Loss of CH_2_O+Sulfate Conjugation [M-H]^-^ | 2.57±0.61 | 3.50±2.78 | 46541.47±4043.95 | 6.15±0.45 | 6993.28±1456.89 |
| M27 | 2-Methyl-3-hydroxyanthraquinone | Oxidation [M-H]^-^ | 2.33±0.69 | 1.67±2.02 | 133805.98±18638.78 | 5.62±1.30 | 26478.39±12536.14 |
| M28 |  | Demethylation to Carboxylic Acid [M-H]^-^ | 2.75±0.46 | 2.83±2.02 | 186100.53±25765.05 | 6.73±0.36 | 25613.92±6729.78 |
| M29 | 2-Hydroxy-3-methoxy-6-methylanthraquinone | Sulfate Conjugation [M-H]^-^ | 3.21±0.25 | 1.67±2.02 | 21077.84±1085.66 | 6.03±0.86 | 4164.94±1095.93 |
| M30 | Rhein | Methylation [M-H]^-^ | 4.11±0.39 | 4.17±3.75 | 74187.86±13099.08 | 7.07±0.64 | 9061.56±730.35 |
| M31 |  | Loss of O+Hydrogenation [M-H]^-^ | 30.31±37.85 | 2.83±2.02 | 12349.56±1232.48 | 4.82±0.35 | 2977.87±724.79 |
| M32 | Emodin | Sulfate Conjugation [M-H]^-^ | 3.07±0.75 | 1.83±1.89 | 23044.87±2777.87 | 7.01±1.02 | 2719.89±801.87 |
| M33 |  | Loss of O and O+Sulfate Conjugation [M-H]^-^ | 2.57±0.61 | 3.50±2.78 | 46541.47±4043.95 | 6.15±0.45 | 6993.28±1456.89 |
| M34 |  | Demethylation to Carboxylic Acid [M-H]^-^ | 7.15±3.51 | 1.00±0.87 | 3859.51±1915.55 | 5.06±1.61 | 1434.47±984.65 |
| M35 |  | Glucuronidation [M-H]^-^ | 3.77±1.02 | 2.83±2.02 | 84122.75±20637.67 | 6.64±1.00 | 12111.63±7451.05 |
| M36 |  | Loss of O+Sulfate Conjugation [M-H]^-^ | 5.73±1.27 | 2.83±2.02 | 18001.79±2118.7 | 7.32±0.15 | 2276.63±528.50 |
| M37 |  | Methylation [M-H]^-^ | 3.40±1.02 | 2.50±3.04 | 15340.33±4889.03 | 4.51±0.90 | 4131.99±286.40 |
| M38 |  | Loss of O [M-H]^-^ | 2.33±0.69 | 1.67±2.02 | 133805.98±18638.78 | 5.62±1.30 | 26478.39±12536.14 |
| M39 |  | Loss of O+Glucuronidation [M-H]^-^ | 4.24±0.22 | 4.17±3.75 | 219416.72±23209.45 | 7.19±0.28 | 26876.51±9662.97 |
| M40 | Chrysophanol | Loss of O+Sulfate Conjugation [M-H]^-^ | 2.57±0.61 | 3.50±2.78 | 46541.47±4043.95 | 6.15±0.45 | 6993.28±1456.89 |
| M41 | Physcion | Glucuronidation [M-H]^-^ | 4.19±1.51 | 4.67±1.16 | 9133.21±1453.09 | 6.43±0.80 | 1528.66±520.69 |
| M42 |  | Loss of O+Sulfate Conjugation [M-H]^-^ | 3.21±0.25 | 1.67±2.02 | 21077.84±1085.66 | 6.03±0.86 | 4164.94±1095.93 |
| M43 |  | Loss of CH_2_O and O [M-H]^-^ | 5.43±2.61 | 3.50±2.78 | 24366.32±2635.93 | 4.73±0.33 | 4650.07±1389.32 |
| M44 |  | Hydrogenation [M-H]^-^ | 5.19±1.73 | 3.50±2.78 | 3062.56±2087.23 | 7.21±0.80 | 739.74±886.95 |
| M45 | Laccaic acid D | Loss of O+Glucuronidation [M-H]^-^ | 3.64±1.36 | 1.67±2.02 | 14720.26±866.40 | 5.76±0.78 | 2726.08±619.12 |
| M46 |  | Loss of O and O [M-H]^-^ | 5.07±1.70 | 1.67±2.02 | 18893.45±1448.64 | 6.84±0.67 | 2600.84±235.93 |
| M47 | 8-Methyl chrysophanol | Sulfate Conjugation [M-H]^-^ | 2.56±0.72 | 0.83±0.29 | 51238.21±12602.74 | 5.98±0.55 | 11395.00±908.14 |
| M48 |  | Oxidation [M-H]^-^ | 3.40±1.02 | 2.50±3.04 | 15340.33±4889.03 | 4.51±0.90 | 4131.99±286.40 |
| M49 |  | Oxidation [M-H]^-^ | 3.40±1.02 | 2.50±3.04 | 15340.33±4889.03 | 4.51±0.90 | 4131.99±286.40 |
| M50 | Rhein-8-*O*-*β*-D-glucopyranoside | Loss of C_6_H_10_O_6_ [M-H]^-^ | 2.75±0.46 | 2.83±2.02 | 186100.53±25765.05 | 6.73±0.36 | 25613.92±6729.78 |
| M51 |  | Loss of C_15_H_6_O_6_ and O+Demethylation and Methylene to Ketone [M-H]^-^ | 18.36±18.06 | 1.00±0.00 | 15113.73±4092.97 | 8.20±0.93 | 3630.91±588.27 |
| M52 | Aloe-emodin-8-*O*-*β*-D-glucopyranoside | Loss of C_6_H_10_O_6_ and O+Loss of Hydroxymethylene [M-H]^-^ | 2.57±0.33 | 4.67±1.16 | 1651688.90±44406.27 | 7.59±0.08 | 148254.08±22243.20 |
| M53 |  | Loss of C_6_H_10_O_6_ [M-H]^-^ | 12.31±7.17 | 0.67±0.29 | 301805.55±73054.62 | 7.44±1.11 | 79528.27±9755.33 |
| M54 | Aloe-emodin-ω-*O*-*β*-D-glucopyranoside | Loss of C_6_H_10_O_5_+Methylation [M-H]^-^ | 3.40±1.02 | 2.50±3.04 | 15340.33±4889.03 | 4.51±0.90 | 4131.99±286.40 |
| M55 |  | Loss of C_6_H_10_O_5_+Methylation [M-H]^-^ | 9.77±4.51 | 0.67±0.29 | 22316.74±5173.83 | 6.19±0.85 | 7183.85±723.79 |
| M56 |  | Loss of C_6_H_10_O_6_ [M-H]^-^ | 12.31±7.17 | 0.67±0.29 | 301805.55±73054.62 | 7.44±1.11 | 79528.27±9755.33 |
| M57 |  | Loss of C_6_H_10_O_6_+Ketone Formation [M-H]^-^ | 2.75±0.46 | 2.83±2.02 | 186100.53±25765.05 | 6.73±0.36 | 25613.92±6729.78 |
| M58 |  | Loss of C_6_H_10_O_6_+Methylation [M-H]^-^ | 2.62±1.38 | 0.67±0.29 | 51390.02±7460.74 | 4.52±0.62 | 15105.85±4254.91 |
| M59 | 6-Methyl-rhein | Glucuronidation [M-H]^-^ | 2.89±1.31 | 4.00±2.00 | 327131.99±40426.01 | 6.78±0.39 | 52235.57±27793.77 |
| M60 |  | Loss of O [M-H]^-^ | 5.07±1.70 | 1.67±2.02 | 18893.45±1448.64 | 6.84±0.67 | 2600.84±235.93 |
| M61 | Emodin-8-*O*-*β*-D-(6-*O*-acetyl) glucopyranoside | Loss of C_8_H_12_O_7_ [M-H]^-^ | 2.33±0.69 | 1.67±2.02 | 133805.98±18638.78 | 5.62±1.30 | 26478.39±12536.14 |
| M62 |  | Loss of C_15_H_8_O_5_ and O [M-H]^-^ | 14.13±7.28 | 3.00±1.73 | 18403.45±1263.85 | 7.93±0.84 | 2419.46±160.88 |
| M63 | Chrysophanol-8-O-(6-O-malonyl)-glucoside | Loss of C_9_H_12_O_9_ [M-H]^-^ | 5.43±2.61 | 3.50±2.78 | 24366.32±2635.93 | 4.73±0.33 | 4650.07±1389.32 |
| M64 | Emodin-8-O-(6-O-malonyl)-glucoside | Loss of C_9_H_12_O_9_+Demethylation to Carboxylic Acid [M-H]^-^ | 4.71±1.74 | 1.67±2.02 | 9221.25±2089.18 | 5.35±0.03 | 3218.58±405.64 |
| M65 |  | Loss of C_9_H_12_O_8_+Methylation [M-H]^-^ | 3.40±1.02 | 2.50±3.04 | 15340.33±4889.03 | 4.51±0.90 | 4131.99±286.40 |
| M66 |  | Loss of C_15_H_8_O_4_ and C_3_H_2_O_4_+Demethylation to Carboxylic Acid [M-H]^-^ | 13.3±1.58 | 1.67±2.02 | 15838.89±1674.85 | 8.31±0.85 | 2225.21±133.63 |
| M67 | 2,3-Dimethoxy-6-methylanthraquinone | Loss of CH_2_+Sulfate Conjugation [M-H]^-^ | 3.21±0.25 | 1.67±2.02 | 21077.84±1085.66 | 6.03±0.86 | 4164.94±1095.93 |
| M68 | 1,3,8-Trihydroxy-6-hydroxymethylanthraquinone | Loss of O+Methylation [M+H]^+^ | 3.65±1.52 | 0.67±0.29 | 24.69±9.07 | 4.23±0.68 | 8.88±1.08 |
| M69 | Phenylalanine | Loss of NH [M-H]^-^ | n.a. | 10.67±2.31 | 49432.39±17484.37 | 10.20±0.64 | 3649.57±1387.48 |
| M70 |  | Glucose Conjugation [M-H]^-^ | 30.30±23.17 | 2.00±1.73 | 157963.09±22081.08 | 9.04±0.59 | 34326.18±5339.86 |
| M71 | (*E*)-6-*O*-*p*-Feruloyl scandoside methyl ester | Loss of CH_2_O and C_6_H_10_O_5_ [M-H]^-^ | 3.53±0.51 | 0.67±0.29 | 5648.59±1191.88 | 5.49±0.89 | 1223.54±391.34 |
| M72 |  | Loss of C_17_H_22_O_10_+Glucuronidation [M-H]^-^ | 3.52±0.19 | 3.50±2.78 | 21738.28±5250.47 | 5.62±0.85 | 3851.71±588.36 |
| M73 | (*E*)-6-*O*-Coumaroyl scandoside methyl ester-10-*O*-methyl ether | Loss of C_9_H_6_O_3_ and O+Loss of Hydroxymethylene [M-H]^-^ | 27.32±14.76 | 2.00±1.73 | 18985.39±1383.02 | 9.61±0.36 | 2640.27±714.60 |
| M74 | Asperulosidic acid | Loss of C_6_H_10_O_6_ and C_2_H_2_O_2_+Sulfate Conjugation [M-H]^-^ | 10.19±3.09 | 4.67±1.16 | 183128.32±64425.72 | 7.69±0.18 | 32400.11±16857.09 |
| M75 |  | Loss of O and O+Demethylation and Methylene to Ketone [M-H]^-^ | 6.64±1.68 | 1.67±2.02 | 5086.24±1082.52 | 6.44±0.76 | 875.85±304.21 |
| M76 | Danshenxinkun B | Hydrogenation [M-H]^-^ | 13.43±2.16 | 1.75±1.98 | 35487.65±4595.69 | 9.67±0.18 | 2764.36±1127.24 |
| M77 | Tormentic acid | Ketone Formation [M-H]^-^ | 10.82±4.71 | 3.00±1.73 | 4594.10±1134.83 | 8.52±2.13 | 821.60±548.15 |
| M78 |  | Loss of O and O+Glucuronidation [M-H]^-^ | 3.16±0.58 | 4.17±3.75 | 47567.55±13473.53 | 6.75±1.23 | 8160.33±5131.81 |
| M79 | Polygonoide C | Loss of C_42_H_66_O_15_ [M-H]^-^ | 15.13±17.47 | 0.50±0.00 | 7797.98±2178.35 | 7.92±3.33 | 1019.53±225.37 |
| M80 |  | Loss of C_36_H_56_O_10_ [M-H]^-^ | 189.51±139.31 | 1.00±0.00 | 26051.98±985.75 | 10.45±0.76 | 3723.80±1363.59 |
| M81 |  | Loss of C_42_H_66_O_15_ and O+Methylation [M-H]^-^ | 34.47±38.23 | 1.67±2.02 | 8084.84±397.08 | 8.42±0.52 | 1295.04±316.87 |
| M82 | Esculetin | Loss of O and O [M+H]^+^ | 3.33±0.55 | 1.58±2.10 | 197.67±26.97 | 4.60±0.84 | 71.81±29.93 |
| M83 |  | Loss of H-2O [M+H]^+^ | 4.02±0.96 | 1.67±2.02 | 37.62±4.49 | 4.68±1.08 | 13.30±7.91 |
| M84 | Lindleyin | Loss of C_10_H_10_O_2_ [M-H]^-^ | 9.77±8.22 | 1.00±0.00 | 16068.50±1520.97 | 6.46±1.36 | 2442.26±290.23 |
| M85 |  | Loss of C_13_H_14_O_9_+Sulfate Conjugation [M-H]^-^ | 2.21±0.17 | 3.50±2.78 | 272192.69±53900.27 | 5.83±0.75 | 44679.70±9983.80 |
| M86 | Isolindleyin | Loss of C_16_H_20_O_7_+Methylation [M-H]^-^ | 6.43±0.63 | 1.67±2.02 | 52295.53±10750.15 | 6.27±1.16 | 9778.38±2375.43 |
| M87 | Adenosine | Loss of C_5_H_3_N_5_+Loss of Hydroxymethylene [M-H]^-^ | 17.68±16.89 | 1.17±0.76 | 134008.46±59718.73 | 9.59±1.35 | 12571.84±1660.75 |
| M88 | *p*-Hydroxybenzoic acid | Glucuronidation [M-H]^-^ | 3.09±0.22 | 0.67±0.29 | 27750.56±6093.22 | 3.56±0.55 | 9582.29±4573.25 |
| M89 |  | Methylation [M-H]^-^ | 3.13±0.11 | 3.00±1.73 | 13911.70±2082.65 | 5.35±0.92 | 2504.45±167.60 |
| M90 | Citric acid | Loss of O [M-H]^-^ | 7.50±1.85 | 1.08±0.88 | 138906.16±24952.63 | 7.25±1.46 | 23282.63±5620.62 |
| M91 | Cinnamic acid | Oxidation [M-H]^-^ | 3.23±0.95 | 1.58±2.10 | 127257.37±3106.94 | 3.97±0.59 | 39818.39±10320.78 |
| M92 | Gallic acid | Loss of O and O+Methylation [M-H]^-^ | 3.13±0.11 | 3.00±1.73 | 13910.67±1995.90 | 5.35±0.92 | 2504.45±167.60 |
| M93 |  | Methylation [M-H]^-^ | 1.96±0.30 | 3.67±2.52 | 30331.09±2762.61 | 5.01±0.62 | 5246.01±847.10 |
| M94 | Protocatechualdehyde | Loss of CO [M-H]^-^ | 273.06±221.81 | 3.00±1.73 | 296712.82±21853.72 | 10.41±0.25 | 29493.74±3380.76 |
| M95 |  | Loss of O [M-H]^-^ | 39.63±19.99 | 4.17±3.75 | 21739.00±2738.37 | 10.78±0.55 | 2363.27±761.56 |
| M96 | Rosmarinic acid | Loss of C_9_H_6_O_4_ and O+Sulfate Conjugation [M-H]^-^ | 5.60±1.00 | 2.83±2.02 | 47648.40±4896.53 | 6.56±0.82 | 8191.94±656.45 |
| M97 |  | Loss of C_9_H_8_O_5_+Glucuronidation [M-H]^-^ | 4.37±0.56 | 1.58±2.10 | 5140.36±187.07 | 5.72±0.78 | 1070.86±242.30 |
| M98 | 4,4'-Dihydroy-*α*-truxillic acid | Loss of O+Methylation [M+H]^+^ | 8.80±3.63 | 4.75±6.34 | 104.46±24.02 | 9.48±1.12 | 9.12±1.19 |
| M99 | 1-*O*-Hexadecyl-*sn*-glycero-3-phosphocholine | Demethylation and Methylene to Ketone [M+H]^+^ | 8.01±4.75 | 1.42±1.01 | 4476.44±865.80 | 6.88±0.93 | 702.36±134.12 |
| M100 |  | Ketone Formation [M+H]^+^ | 16.86±12.51 | 1.50±0.87 | 183.91±37.31 | 8.11±1.73 | 29.79±4.67 |
| M101 | 1-*O*-Hexadecanoyl-*sn*-glycero-3-phosphocholine | Desaturation [M+H]^+^ | 6.61±3.09 | 0.92±0.95 | 296.71±79.79 | 5.86±0.86 | 62.83±16.73 |
| M102 |  | Desaturation [M+H]^+^ | 5.52±1.32 | 1.67±2.02 | 10552.21±1831.31 | 7.26±0.99 | 1798.72±683.11 |
| M103 | 1-*O*-Octadecanoyl-*sn*-glycero-3-phosphocholine | Ethyl to Alcohol [M+H]^+^ | 87.35±116.48 | 4.17±3.75 | 24.88±18.77 | 4.91±1.42 | 8.01±3.64 |
| M104 |  | Oxidation [M+H]^+^ | 7.01±3.99 | 1.58±2.10 | 2.62±1.89 | 5.61±0.67 | 1.39±1.09 |
| M105 |  | Desaturation [M+H]^+^ | 7.55±2.48 | 1.67±2.02 | 38.71±5.04 | 7.21±0.60 | 8.17±1.52 |

n.a. the pharmacokinetic parameters are not available due to limited amounts of data points.
